# Supplementary material for: Conflict violence reduction and pregnancy outcomes: A regression discontinuity design in Colombia
Source: PLoS Med. 2021 Jul 6;18(7):e1003684. doi: 10.1371/journal.pmed.1003684 (PMC8259980; doi:10.1371/journal.pmed.1003684)
Supplement: S1 Protocol — (PDF) [file pmed.1003684.s002.pdf]

EFFECTS OF ARMED CONFLICT DURING THE FIRST THREE MONTHS OF LIFE ON MORTALITY AND THE USE OF HEALTH SERVICES AMONG INFANTS UNDER 1 YEAR OF AGE

Giancarlo Buitrago MD PhD
Rodrigo Moreno Serra PhD

Bogota
2018

CONTENT

1. INTRODUCTION 3
2. QUESTION 4
3. OBJECTIVES 5
   3.1. Overall objective 5
   3.2. Specific objectives 5
4. METHODS 6
   4.1. Type of study 6
   4.2. Population 6
   4.3. Sample size 6
   4.4. Variables 6
      4.4.1. Exposure variable 6
      4.4.2. Outcome variables 8
      4.4.3. Control variables 9
   4.5. Analysis 9
      4.5.1. Regression Discontinuity 9
5. ETHICAL ASPECTS 11
   5.1. Research and Ethics Committee (CEI in Spanish) 11
   5.2. Ethical execution of the study 11
   5.3. Confidentiality 11
6. HUMAN RESOURCES 12
   6.1. Principal Investigator 12
   6.2. Co-researchers 12
7. TIMELINE 13
8. REFERENCES 14

1. INTRODUCTION

Decreasing infant mortality is one of the main goals of the global community. The Millennium Development Goals established the aim of reducing infant mortality by two-thirds by the year 2015, and although good progress has been made, this goal has not been reached. Current Sustainable Development Goals include decreasing neonatal mortality, and to very low figures by the year 2030 for children under 5 years of age. The majority of infant deaths is preventable. Their causes vary and are closely related with social determinants and the population's effective access to health services [1, 2].

Over recent years, the literatures on both economics and health have reported evidence of the negative effects of armed conflict on children's health [3–6].[1]

Colombia is a country that has experienced armed conflict for over 50 years, where an estimated 220,000 deaths have occurred as a result. Large geographic differences exist in the infant mortality rate in Colombia. The National Administrative Department of Statistics (DANE in Spanish) estimated a mortality rate among infants under 1 year of age of 17.23 per 1,000 live births (LB) for the year 2014.[2] Large differences have also been found among the country's municipalities, with municipal rates ranging from under 10 to over 70 infant deaths per 1,000 LB. Although the cause of these differences can be explained by the convergence of many factors, armed conflict and the variation in its intensity are among the reasons and can directly affect mortality in infants under 1 year of age.[3]

In the year 2012, the Colombian Government initiated talks in Havana with FARC[4] forces in order to end the conflict. In 2016, the cycle of talks with FARC concluded with the signing of the peace accords. It is very important to identify the possible consequences of the end of the conflict with FARC on infant health. To do so, the effect of the armed conflict on infant mortality needs to be separated from the effect of other determinants.[5] These results are highly relevant for Colombia as well as for other countries in situations involving armed conflict.

Performing an adequate diagnostic of the effect of exposure to armed conflict during pregnancy and early infancy on outcomes that are as important as mortality and the use of health services is the first step towards generating an overall picture that shows us what those consequences have been and provides the basis on which challenges and their possible solutions could begin to be proposed.

---

[1] The main outcomes of interest evaluated by these studies were anthropometric measurements.
[2] Source: DANE, see: http://dane.gov.co/files/investigaciones/poblacion/vitales/Bol_TMI_DCD_2014.pdf
[3] For example, it is clear that many of the municipalities with high infant mortality rates are also those where the intensity of armed conflict was high.
[4] FARC is the largest rebel group in Colombia.
[5] There is no empirical evidence of high quality worldwide, and there is no type of evidence in Colombia.

2. QUESTION

What is the effect of exposure to armed conflict during pregnancy, birth and the first three months of life on mortality and the use of health services among infants under 1 year of age in Colombia?

3. OBJECTIVES

3.1. Overall objective

To determine the effect of exposure to armed conflict during pregnancy, birth and the first three months of life on mortality and the use of health services among infants under 1 year of age in Colombia.

3.2. Specific objectives

1. To identify the municipalities exposed and not exposed to armed conflict and the intensity of the conflict during the Havana talks between the Colombian government and FARC forces.
2. To describe the rates of miscarriages, stillbirths, perinatal deaths, and death of infants under 1 year of age from the municipalities affected and not affected by the armed conflict during the Havana talks between the Colombian government and FARC forces.
3. To determine the rate of use of emergency and hospital services by infants under 1 year of age from municipalities affected and not affected by armed conflict during the Havana talks between the Colombian government and FARC forces.
4. To compare the rate of mortality and use of hospital services among infants under 1 year of age in municipalities affected and not affected by armed conflict during the Havana talks between the Colombian government and FARC forces.

4. METHODS

4.1. Type of study

Retrospective study of cohorts of all pregnant women and infants born between January 2013 and December 2017 in Colombia (includes a regression discontinuity design [7, 8]).

4.2. Population

Two cohorts will be generated:

- Exposed cohort: to include all pregnant women and infants born in the municipalities in Colombia during periods when FARC implemented partial ceasefires during the Havana talks.
- Unexposed cohort: to include all pregnant women and infants born in the municipalities in Colombia during periods when FARC did not implement partial ceasefires during the Havana talks.

4.3. Sample size

Partial analyses of the information show that during this observation period there were over 3 million pregnant women in the entire country. The entire universe of pregnant women and live births recorded in the vital statistics records will be used.

4.4. Variables

As will be presented next, for each pregnancy and infant included in the cohort, this study will identify exposure to armed conflict during pregnancy, birth and at least the first month of life, whether there is a death and effective consumption of health services during the first year of life.[6]

4.4.1. Exposure variable

The exposure to armed conflict that will be evaluated will be exposure that affects the infant at the time of birth and continuously until the first month of life. The following information will be used to identify exposure.

4.4.1.1. Ceasefires during the Havana talks (exogenous treatment)

During the Havana peace talks, FARC declared a unilateral ceasefire seven times as a sign to the Colombian government of "good faith." These primarily correspond to dates related

[6] All the sources of information presented in this document are anonymized under the responsibility of Giancarlo Buitrago, and exclusively for use in research.

with national holidays, elections and when the talks were making progress. A ceasefire declared during the Havana talks can be interpreted to be exogenous treatment since they occurred during political negotiations and were nationwide. This study will use only the ceasefires having over three months of continuity, which correspond to the following periods: [7,8]

1. Fifth unilateral and indefinite "ceasefire," December 20, 2014, to May 22, 2015, suspended after bombing by the Colombian military.
2. Sixth unilateral and indefinite "ceasefire" from July 20, 2015, until the signing of the peace accord.
3. Seventh unilateral and indefinite "ceasefire," August 29, 2015, declared by the Colombian government.

These periods of ceasefire did not affect all the municipalities, only those that historically had been under FARC control. The following map represents these municipalities in the year 2012.[9]

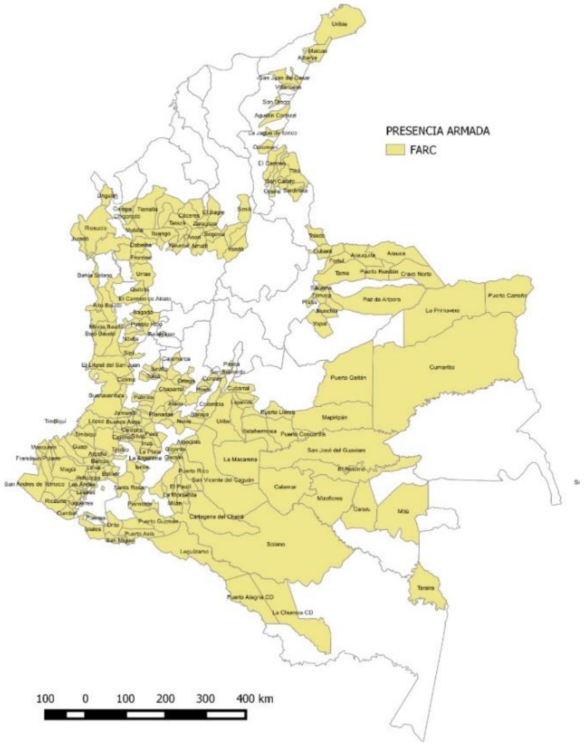

Source: *Fundación Paz y Reconciliación*

---

[7] Source: https://www.farc-ep.co/unilateral.html

[8] Other ceasefires were declared by FARC, but do not meet the minimum time period of three months: i) first unilateral and temporary "ceasefire" from November 20, 2012 to January 20, 2013; ii) second unilateral and temporary "ceasefire" from December 15, 2013 to January 15, 2014; iii) third unilateral and temporary "ceasefire" from May 20, 2014 to May 28, 2014; and iv) fourth unilateral and temporary "ceasefire" from June 9, 2014 to June 30, 2014.

[9] Source: http://www.pares.com.co/paz-y-posconflicto/grupos-armados-ilegales/farc/los-mapas-del-conflicto/

4.4.1.2. Intensity of exposure

To identify the intensity of the exposure to the armed conflict during the observation period (2012 – 2017), a monthly municipal panel will be designed, which will include information from reports of victims associated with conflict, combat, attacks and extortion claims associated with FARC and other armed groups. The following sources of information will be used:

1. CERAC database: http://www.cerac.org.co/es/recursos/datosconflictoscolombia/
2. Centro Nacional de Memoria Histórica (National Historical Memory Center): http://www.centrodememoriahistorica.gov.co/micrositios/informeGeneral/estadisticas.html
3. Municipal CEDE panel (violence section): https://datoscede.uniandes.edu.co/es/
4. Colombian Longitudinal Survey of the Andes (ELCA in Spanish) : based on the measurements obtained by ELCA, the intensity of the conflict at the municipal level will be identified as a measure of exposure to conflict. See https://encuestalongitudinal.uniandes.edu.co/es/

4.4.2. Outcome variables

The following outcome variables will be used: i) mortality from any cause during pregnancy and until the first year of life and ii) effective use of health services during the pregnancy and until the first year of life. The following sources will be used to identify each of these.

4.4.2.1. Mortality of pregnant women and infants during pregnancy and until the first year of life

Data from birth and death certificates will be used to identify pregnant women and infants who died during pregnancy and during the first year of life.

4.4.2.2. Consumption of health care resources during the pregnancy and until first year of life

Colombia's National Social Security Health System covers approximately 97% of the population. Based on national sources of health system data, the study will identify the resources consumed by all the pregnant women and their infants in the cohort during the first year of life. In particular, the following services will be identified:

i) Prenatal controls
ii) Outpatients visits for general and specialized medicine
iii) Hospitalizations
iv) Emergency department visits
v) Intensive care unit admissions

The following sources of information will be used:

1. Capitation Payment Unit (UPC in Spanish) sufficiency database (years 2012 – 2015). This database of information contains all the services that the Health Care Provider Companies (EPS in Spanish) paid under the UPC. [10]
2. Individual Registry of Health Services Provided (RIPS in Spanish). Information from RIPS will be used, which contains all the health care provided by each of the health providers in the country.

4.4.3. Control variables

Based on the information from the birth certificates, death certificates and the UPS and RIPS datasets, the characteristics of the mother, the pregnancy and the family will be identified to serve as control for the impact analysis. In particular, preexisting comorbidities, ages and the educational levels reported will be identified.

4.5. Analysis

The main analytical approach will be a regression discontinuity design. Nevertheless, if the assumptions of the regression discontinuity are not met, other strategies will be used to accomplish the study's objective.

4.5.1. Regression Discontinuity

We will use a regression discontinuity (RD), a quasi-experimental study design, to permit causal inference in the absence of treatment randomization. In this design, the conflict violence exposure ("treatment") status of each individual is determined by a threshold of a continuous variable. This assignment rule generates a discontinuity in the probability of exposure, and under certain conditions this assignment is as good as random for individuals near to the threshold. Therefore, this analytical design can identify the causal effect of exposure for these individuals. Exposure assignment following such a rule may be either deterministic (there is a deterministic change of the probability of being exposed) or nondeterministic (the probability of being exposed is higher on one side of the threshold than on the other side). The approach in the first case is called sharp RD and, in the second, fuzzy RD.

The ceasefires declared by FARC generated a discontinuous decrease in the number of conflict events to which women who started their pregnancy or infants who born just after the ceasefire were exposed. Thus, we used the ceasefires as thresholds of the RD design to

[10] The main researcher (Giancarlo Buitrago) currently has the information from the UPC database only for the contributory system. Nevertheless, a request will be sent to the Ministry of Health to obtain the information from the subsidized system.

estimate the causal effect of a decrease in exposure to armed conflict events during pregnancy on the risks of miscarriage, stillbirth, perinatal mortality, and mortality during the first year of life.

The strategy proposed by Calonico, Cattaneo and Titiunik [9] will be used for this estimation, in which the effect will be estimated based on differences in the expected values of the outcome variable when the score variable (in this case the date of conception or date of birth) approaches the cut-off, from the right or the left. This expected value will be obtained using a first-order polynomial regression. Standard errors will be adjusted with a higher-order polynomial [10]. Since the ceasefires were broken at times, a fuzzy regression discontinuity will be used based on the violent events registered in the municipal panel from the various sources of information mentioned previously. Graphs will be generated and standard statistical tests will be performed to ensure non-manipulation of the assumptions and the continuity of baseline variables [7].

4.5.2 Instrumental variables

As mentioned before, if the assumptions of the regression discontinuity are met, then two additional methodological approaches will be used. The first proposal will be to analyze the instrumental variables, in which the violent events in the municipalities in which the pregnant women and infants in the cohort were born will be compared with the ceasefires declared by FARC [11]. Two-stage least squares will be performed. In the first stage, a regression will be run between the number of monthly violent events per municipality and the ceasefires declared by FARC, and the predicted values of this first model will be estimated. The second stage will consist of running regressions between the outcome variables and the predicted values from the first model. For an instrument to be adequate, it must meet at least two assumptions: exogeneity and relevance. As mentioned, a ceasefire is considered to be an exogenous intervention, and therefore, it will only be related with the outcome variables through the exposure to violent events during pregnancy and after birth during the first month of life. The relevance of the instrument is clear, the ceasefire decreased violent events by FARC.

4.5.3. Differences in differences

Lastly, an analysis of differences in differences will be conducted [12]. A control group of all municipalities without the presence of FARC will be used, which will be divided into municipalities with armed conflict (ELN and others) and those without the presence of armed groups. The treatment group will be municipalities that have been under FARC control. There will be pre- and post-treatment groups corresponding to the periods of the ceasefires. As mentioned in the regression discontinuity analysis, only pregnant women and infants who are guaranteed to have at least one month of exposure will be included. The assumption of parallel trends will be evaluated, and in their absence, paired methods will be used with propensity indices, based on the control variables presented previously.

5. ETHICAL ASPECTS

This study will not perform any interventions whatsoever that involve the observation of patients or individuals. The research will not influence the individual treatment of each case. This is considered to be a NO RISK study for the individuals, in accordance with Article 11 of 1993 Ministry of Health Resolution 8430, and this study fully respects all provisions in Article 8 of that resolution.

5.1. Research and Ethics Committee (CEI in Spanish)

The study protocol and all its revisions will be reviewed and approved by a CEI. This protocol may be revised at any time. Any systematic change in the activity of the investigation will require approval by the CEI before implementing.

5.2. Ethical execution of the study

This study will be performed in accordance with the protocol, good clinical practices, and other pertinent privacy regulations, as well the INVIMA regulations that govern the execution of clinical studies and ethical principles, which are based on the Helsinki Declaration.

5.3. Confidentiality

The confidentiality of the information obtained will be ensured. The researchers have the technical skills required to perform the study. Only during the preparation of an oral presentation, poster or manuscript can the unidentifiable data be accessed. Identifiable information will not be shared in any publications.

6. HUMAN RESOURCES

This project will be conducted with the help of the following human resources.

6.1. Principal Investigator

Giancarlo Buitrago, MD, MSc, PhD. Physician. Master in Clinical Epidemiology. Master in Economics. Doctor of Economics.

6.2. Co-researchers

1. Rodrigo Moreno-Serra. Co-researcher. Economist. Doctor in Economics

7. TIMELINE

Time in months: 12

Activities	1	2	3	4	5	6	7	8	9	10	11	12
Presentation to the Research and Ethics Committee												
Literature search												
Request for Ministry of Health and Social Protection database												
Requests for databases from other entities												
Design of the municipal panel												
Identification of cohorts based on ceasefires												
Analysis of information (York)												
Writing of articles												
Presentations at conferences												

8. REFERENCES

1. Bairoliya N, Fink G (2018) Causes of death and infant mortality rates among full-term births in the United States between 2010 and 2012: An observational study. PLoS Med 15:e1002531 . doi: 10.1371/journal.pmed.1002531
2. Ely DM, Hoyert DL (2018) Differences Between Rural and Urban Areas in Mortality Rates for the Leading Causes of Infant Death: United States, 2013-2015. NCHS Data Brief 1–8
3. Akresh R, Lucchetti L, Thirumurthy H (2012) Wars and child health: Evidence from the Eritrean–Ethiopian conflict. J Dev Econ 99:330–340 . doi: 10.1016/j.jdeveco.2012.04.001
4. Bundervoet T, Verwimp P, Akresh R (2009) Health and Civil War in Rural Burundi. J Hum Resour 44:536–563 . doi: 10.1353/jhr.2009.0000
5. Joshi M (2015) Comprehensive peace agreement implementation and reduction in neonatal, infant and under-5 mortality rates in post-armed conflict states, 1989–2012. BMC Int Health Hum Rights 15: . doi: 10.1186/s12914-015-0066-7
6. Minoiu C, Shemyakina ON (2014) Armed conflict, household victimization, and child health in Côte d'Ivoire. J Dev Econ 108:237–255 . doi: 10.1016/j.jdeveco.2014.03.003
7. Bor J, Moscoe E, Mutevedzi P, et al (2014) Regression discontinuity designs in epidemiology: causal inference without randomized trials. Epidemiol Camb Mass 25:729–737 . doi: 10.1097/EDE.0000000000000138
8. Moscoe E, Bor J, Bärnighausen T (2015) Regression discontinuity designs are underutilized in medicine, epidemiology, and public health: a review of current and best practice. J Clin Epidemiol 68:122–133 . doi: 10.1016/j.jclinepi.2014.06.021
9. Calonico S, Cattaneo MD, Titiunik R (2014) Robust Nonparametric Confidence Intervals for Regression-Discontinuity Designs: Robust Nonparametric Confidence Intervals. Econometrica 82:2295–2326 . doi: 10.3982/ECTA11757
10. Calonico S, Cattaneo MD, Farrell MH (2017) On the Effect of Bias Estimation on Coverage Accuracy in Nonparametric Inference. J Am Stat Assoc 1–13 . doi: 10.1080/01621459.2017.1285776
11. Baiocchi M, Cheng J, Small DS (2014) Instrumental variable methods for causal inference. Stat Med 33:2297–2340 . doi: 10.1002/sim.6128
12. Grabich SC, Robinson WR, Engel SM, et al (2015) County-level hurricane exposure and birth rates: application of difference-in-differences analysis for confounding control. Emerg Themes Epidemiol 12:19 . doi: 10.1186/s12982-015-0042-7

28 de junio de 2018 FM-CIE-0283-18

Doctor
GIANCARLO BUITRAGO
Investigador Principal
Pontificia Universidad Javeriana
Bogotá

Ref. "Efectos del conflicto armado durante los primeros tres meses de vida en la mortalidad y la utilización de servicios de salud en niños menores de 1 año de edad".
2018/109

Apreciado doctor Buitrago.
El Comité de Investigaciones y Ética Institucional de la Facultad de Medicina de la Pontificia Universidad Javeriana declara su total adherencia a los principios éticos y científicos citados en la declaración de Helsinki, versión 64ª Asamblea General, Fortaleza, Brasil, octubre 2013. Además, certifica que se rige para la conducción de estudios por las buenas prácticas clínicas aceptadas internacionalmente y las normas vigentes de la legislación colombiana.

Por medio de la presente le comunico que en la sesión ordinaria del 14/06/2018 **Nº de Acta (10/2018)**, el Comité revisó el proyecto y **lo aprobó** por consenso. Se presentó toda la documentación necesaria para evaluar el estudio. Los miembros de equipo de investigación son idóneos para participar. El proyecto es pertinente y cumple con el rigor metodológico para este tipo de diseños. Las consideraciones éticas se adhieren a las normas nacionales e internacionales, permiten determinar que se realizará una selección justa de los sujetos y que se respetarán sus derechos y se tomarán las medidas que garanticen su seguridad y bienestar. La relación riesgo beneficio es favorable para los sujetos.

Durante la reunión correspondiente a esta comunicación se encontraron presentes 10 de los 15 miembros del Comité.

CARLOS GÓMEZ-RESTREPO.
Médico, Especialista en Psiquiatría, Magíster en Epidemiología Clínica. Presidente

VICENTE DURÁN CASAS S. J.
Miembro de la Compañía de Jesús. Doctorado en Filosofía. Vocal.

JUAN GUILLERMO CATAÑO CATAÑO.
Médico, Especialista en Urología. Vocal.

FERNANDO SUÁREZ OBANDO
Médico, Especialista en Genética Clínica, Magister en Epidemiología Clínica, Especialista en Bioética. Vocal.

ÁNGEL ALBERTO GARCÍA PEÑA.
Médico, Especialista en Medicina Interna y Cardiología, Magíster en Epidemiología Clínica. Vocal.

SAÚL JAVIER RUGELES
Médico, Especialista en Cirugía Gastrointestinal y Endoscopia Digestiva, Especialista en Metabolismo y Soporte Nutricional. Vocal.

ÁNDRES DUARTE OSORIO
Médico, Especialista en Medicina Familiar, Magister en Epidemiología Clínica. Vocal.

DIANA LUCÍA MATALLANA ESLAVA
Psicóloga. Vocal.

FM-CIE-0283-18

PABLO ASCHNER MONTOYA
Médico, Especialista en Endocrinología, Magister en Epidemiología clínica. Vocal.

ÁLVARO DÁVILA LADRÓN DE GUEVARA
Economista. Representante de la Comunidad. Vocal.

"*La aprobación de este proyecto por parte del CIEI no implica que la Pontifica Universidad Javeriana o el Hospital Universitario San Ignacio estén en la obligación de financiar los rubros descritos en el presupuesto*".

Atentamente,

PONTIFICIA UNIVERSIDAD JAVERIANA
FACULTAD DE MEDICINA
COMITÉ DE INVESTIGACIONES Y ÉTICA
Bogotá Colombia

CARLOS GÓMEZ-RESTREPO MD
Presidente Comité de Investigaciones y Ética

Copia: Archivo de Comité de Investigaciones y Ética
