# Supplementary material for: Conflict violence reduction and pregnancy outcomes: A regression discontinuity design in Colombia
Source: PLoS Med. 2021 Jul 6;18(7):e1003684. doi: 10.1371/journal.pmed.1003684 (PMC8259980; doi:10.1371/journal.pmed.1003684)
Supplement: S1 Text — Fig A: Flow diagram of the data linkage process. Fig B: Cease-fires declared during the Havana talks. Fig C: Historic presence of armed groups in Colombia (2000 to 2017). Fig D: Trend in the number of conflict events to which pregnant women were exposed during pregnancy in Colombia between January 2013 and December 2017. Fig E: Effects of the July 20, 2015 and August 28, 2016 cease-fires on the exposure to FARC-related conflict events during pregnancy: Colombia and categories of municipalities (RD plots). Fig F: Tests of the RD no-manipulation assumption around the July 20, 2015 cease-fire threshold. Fig G: Tests of balance in baseline characteristics around the July 20, 2015 cease-fire for women in M-p90 municipalities. Fig H: Tests of balance in baseline characteristics around the July 20, 2015 cease-fire for women in M-p75 municipalities. Table A: Effects of the July 20, 2015 cease-fire on fetal deaths and perinatal mortality. Table B: Effects of the July 20, 2015 cease-fire on fetal deaths and perinatal mortality. LLR and parametric regressions by order of polynomial for M-p90 municipalities. Table C: Effects of the July 20, 2015 cease-fire on fetal deaths and perinatal mortality. LLR and parametric regressions by order of polynomial for M-p75 municipalities. FARC, Fuerzas Armadas Revolucionarias de Colombia; LLR, local linear regression; RD, regression discontinuity. (DOCX) [file pmed.1003684.s003.docx]

**Table of contents**

[**A.** **Information sources and databases** 1](#_Toc74160992)

[A.1. Description of sources and quality of information 2](#_Toc74160993)

[A.2. Permissions to use the information sources 3](#_Toc74160994)

[A.3. Construction of the database 3](#_Toc74160995)

[Figure A. Flow diagram of the data linkage process 5](#_Toc74160996)

[**B.** **Context of the armed conflict in Colombia and peace process** 6](#_Toc74160997)

[Figure B. Ceasefires declared during the Havana talks 6](#_Toc74160998)

[Figure C. Historic presence of armed groups in Colombia (2000-2017) 7](#_Toc74160999)

[Figure D. Trend in the number of conflict events to which pregnant women were exposed during pregnancy in Colombia between Jan-2013 and Dec-2017 8](#_Toc74161000)

[B.1. Definitions of types of armed conflict events 8](#_Toc74161001)

[**C.** **Regression discontinuity design** 11](#_Toc74161002)

[**D.** **The effects of ceasefires on exposure to conflict events** 12](#_Toc74161003)

[Figure E. Effects of the July 20th, 2015 and August 28th, 2016 ceasefires on the exposure to FARC-related conflict events during pregnancy: Colombia and categories of municipalities (RD plots) 12](#_Toc74161004)

[**E.** **Analyses of the validity of RD assumptions** 14](#_Toc74161005)

[E.1. No-manipulation assumption 14](#_Toc74161006)

[Figure F. Tests of the RD no-manipulation assumption around the July 20th, 2015 ceasefire threshold 15](#_Toc74161007)

[E.2. Continuity of baseline characteristics assumption 16](#_Toc74161008)

[Figure G. Tests of balance in baseline characteristics around the July 20^th^, 2015 ceasefire for women in M-p90 municipalities 16](#_Toc74161009)

[Figure H. Tests of balance in baseline characteristics around the July 20^th^, 2015 ceasefire for women in M-p75 municipalities 18](#_Toc74161010)

[**F.** **Robustness checks** 20](#_Toc74161011)

[Table A. Effects of the July 20th, 2015 ceasefire on fetal deaths and perinatal mortality 20](#_Toc74161012)

[F.1. RD specification changes to estimate the effect of the July 20th, 2015 ceasefire on fetal deaths and perinatal mortality 21](#_Toc74161013)

[Table B. Effects of the July 20th, 2015 ceasefire on fetal deaths and perinatal mortality. LLR and parametric regressions by order of polynomial for M-p90 municipalities 21](#_Toc74161014)

[Table C. Effects of the July 20th, 2015 ceasefire on fetal deaths and perinatal mortality. LLR and parametric regressions by order of polynomial for M-p75 municipalities 22](#_Toc74161015)

[**G.** **Effect of the July 20th, 2015 ceasefire on prenatal care utilization** 23](#_Toc74161016)

[Table D. Effects of the July 20th, 2015 ceasefire on prenatal care visits per month 23](#_Toc74161017)

[**H.** **References** 24](#_Toc74161018)

1. **Information sources and databases**
   1. Description of sources and quality of information

We included in our study all women who began their pregnancies between January 1st, 2013, and December 31st, 2017, and who were followed up until their pregnancy ended in either a live birth or fetal death. Subsequently, their live-born children were followed until day 7 of life. Each woman included in the study was assigned a level of exposure to armed conflict during pregnancy. To identify this population of pregnant women, some individual characteristics, and the levels of conflict to which they were exposed, we used the following databases:

1. Our main source of information was the **Single Registry of Enrollees, Module ND** (RUAF, from Spanish name), which is administered by the Ministry of Health and Social Protection. This is the main source that the National Administrative Department of Statistics (*Departamento Administrativo Nacional de Estadística,* or DANE) uses to generate the country's vital statistics. RUAF was created in 2007 through the *Circular Externa Conjunta* No. 0081 of November 13rd, 2007. RUAF contents and its operation have been assessed by international institutions, which have concluded that the system has made great progress since its establishment in terms of coverage, completeness, and timeliness.^[[1]](#footnote-1)^ 99% of the births reported in Colombia between 2012 and 2016 were reported in the ND module and for 2016, 91% of the deaths were reported in the ND module. The main reason for the latter gap was that not all deaths verified by the National Institute of Legal Medicine and Forensic Sciences were registered in RUAF’s Module ND (these deaths are related to external causes, namely homicides and traffic accidents).^[[2]](#footnote-2)^
2. The source of information about armed conflict events was the **National Center for Historical Memory** (*Centro Nacional de Memoria Histórica,* or CNMH) dataset, which is a leading data source for research about the Colombian conflict due to its reliability and completeness. The CNMH database is a leading data source for research about the Colombian conflict due to its reliability and completeness. It contains comprehensive, publicly available information on all conflict-related violence events by location (terrorist attacks, war actions, attacks on populations, selective murders, kidnappings, child recruitment, massacres, enforced disappearance, damage to property, sexual violence, and landmines). Each event has an associated calendar date and reported number of victims. The data is based on reports from 592 different sources (e.g. military forces, national police, governmental sources, non-governmental organizations, media reports, reports by victims), with a timeframe going back to 1958 and the municipality as the lowest geographic unit. We downloaded the publicly available CNMH dataset from <http://centrodememoriahistorica.gov.co> on December 8^th^, 2018. A full description of the methodology for construction of the CNMH dataset can be found (in Spanish) at: <http://centrodememoriahistorica.gov.co/observatorio/metodologia/guia-metodologica/>

Other secondary sources of information were:

1. **Unique Affiliation Database** (*Base de Datos Única de Afiliación*, or BDUA). BDUA is the official government registry tool for tracking and recording individual enrollee status in the Colombian health system, i.e. individual registration into one of the alternative health insurance schemes (subsidized, contributory and others). This database also includes basic socio-demographic characteristics of enrollees.
2. **Calculation Study of the Capitation Unit Database** (*Base del Estudio de Suficiencia de la Unidad Por Capitación*, or UPC). The UPC database contains detailed records of each episode of healthcare service use by each Colombian registered into the country’s health system, i.e. enrolled into one of the health insurances schemes. The Ministry of Health shares exclusively the UPC base of the Contributory scheme (it does not share that of the subsidized scheme due to quality problems of the information source). This database includes personal identification information, location of service use, date of service, specific type of service, any diagnostic information, identity (and type) of health professional who provided the service, and payments/reimbursements for the service. The UPC is the database used by the Ministry of Health and Social Protection for the calculation of risk-adjusted capitated payments added to the insurance premiums paid to health insurers.
   1. Permissions to use the information sources

The Clinical Research Institute of the School of Medicine at Universidad Nacional de Colombia made a formal request to the Office of Information Technology and Communication of the Ministry of Health and Social Protection to obtain the sources of information mentioned previously (with the exception of the CNMH database, which is publicly available), with the stated reason for this request being to use such data sources in several research projects (File No. 201842301411402 from September 14th, 2018). The Ministry of Health granted our request and provided the databases in question to the Clinical Research Institute (including an anonymous identifier that allowed the different databases to be linked), through communications from March 5th, March 21st, and May 27th, 2019. In these communications, the Ministry of Health authorizes the Clinical Research Institute to carry out academic research with these databases, under the condition that researchers share the research results with the Ministry.

Our study received IRB approval from the Research and Institutional Ethics Committee of the School of Medicine at the Pontificia Universidad Javeriana in the ordinary session of June 14th, 2018 (Minutes No. 10/2018), while Buitrago (first author) was a full-time professor at Pontificia Universidad Javeriana.

- 1. Construction of the database

We constructed the database used for all the analyses in our study through the following steps:

1. Both the birth and death certificates for children under one year of age contain an identifier of the mother, which corresponds to the national identification number (NUMIDENMAD variable in both certificates). This identifier is anonymized by the Ministry of Health but allows the linkage between our data sources. We used all the information from the birth certificates between 2013 and 2018 (3 661 022 records). We also used the records of fetal deaths and deaths up to day 7 of life from the death certificates between 2013 and 2018 (335 340 records). We included information from birth and death certificates up to 2018 (inclusive) because pregnancies that began at the end of 2017 could have ended in 2018.
2. For all 3 996 362 records, the probable conception date was calculated from the gestational age information (TIEMGESTAC variable in the birth certificate; TIEGESSEMMAD variable in the death certificate). A total of 682 409 records (17.08%) were excluded because they were outside the study period (i.e. probable date of conception before January 1st, 2013, or after December 31st, 2017). 59 257 records (1.48%) associated with multiple pregnancies were also excluded. At the end, the total population included in the study was 3 254 696 women with singleton pregnancies.
3. 11 different databases in Excel format were downloaded from CNMH (<http://centrodememoriahistorica.gov.co>) on December 8^th^, 2018. Each database contains information on one type of conflict event: terrorist attack, act of war, attack on populations, selective murders, kidnapping, child recruitment, massacres, forced disappearance, damage to property, sexual violence, and landmine. The 11 databases were merged into a single database that contained 280 241 records of conflict events between 1958 and 2018. 1.11% (n = 3 102) did not have information on the date of occurrence of the event and 1.87% (n = 5 241) did not have information on the place of occurrence. A total of 8 397 conflict events were recorded between 2013 and 2018. Conflict events were categorized into FARC-related events (from information provided in the CNMH database about armed actors involved in each event) and total events.
4. We matched the pregnancy and conflict databases described above by using the municipality of residence code of each of the 3 254 696 women included in the study population, and the municipality of occurrence code of each conflict event. This enabled us to generate the conflict exposure variable for each pregnant woman. Individual conflict exposure was defined as the total number of conflict events that occurred in the municipality of residence during the duration of the pregnancy.
5. Finally, based on the CNMH information, we determined the distribution of the total number of conflict events related to the FARC between 2000 and 2017 for all 1 122 Colombian municipalities. With this information we then constructed the different municipality categories (M-p90, M-p75, M-zero and M-other, as described in the Methods section of our paper).
6. The UPC database was used to confirm that the municipality in which a woman resided during her pregnancy was indeed the municipality identified in the birth or death certificate. We could only cross-check such information for 35.41% of the pregnancies unfortunately, because the UPC information available only covers healthcare use by individuals affiliated to the contributory health insurance scheme (which covers 42.52% of the pregnant women included in our study). We found that in most of these cases (88.18%) the residence information in the birth/death certificates was corroborated by the UPC information. Finally, the BDUA database was used to complete missing information about health insurance status and age, but exclusively when this information was not present in the birth or death certificates (<3% of observations).

Figure A shows the flow diagram for the construction of our database from primary sources (blue boxes) to the final database with full information (red box).

Figure A. Flow diagram of the data linkage process


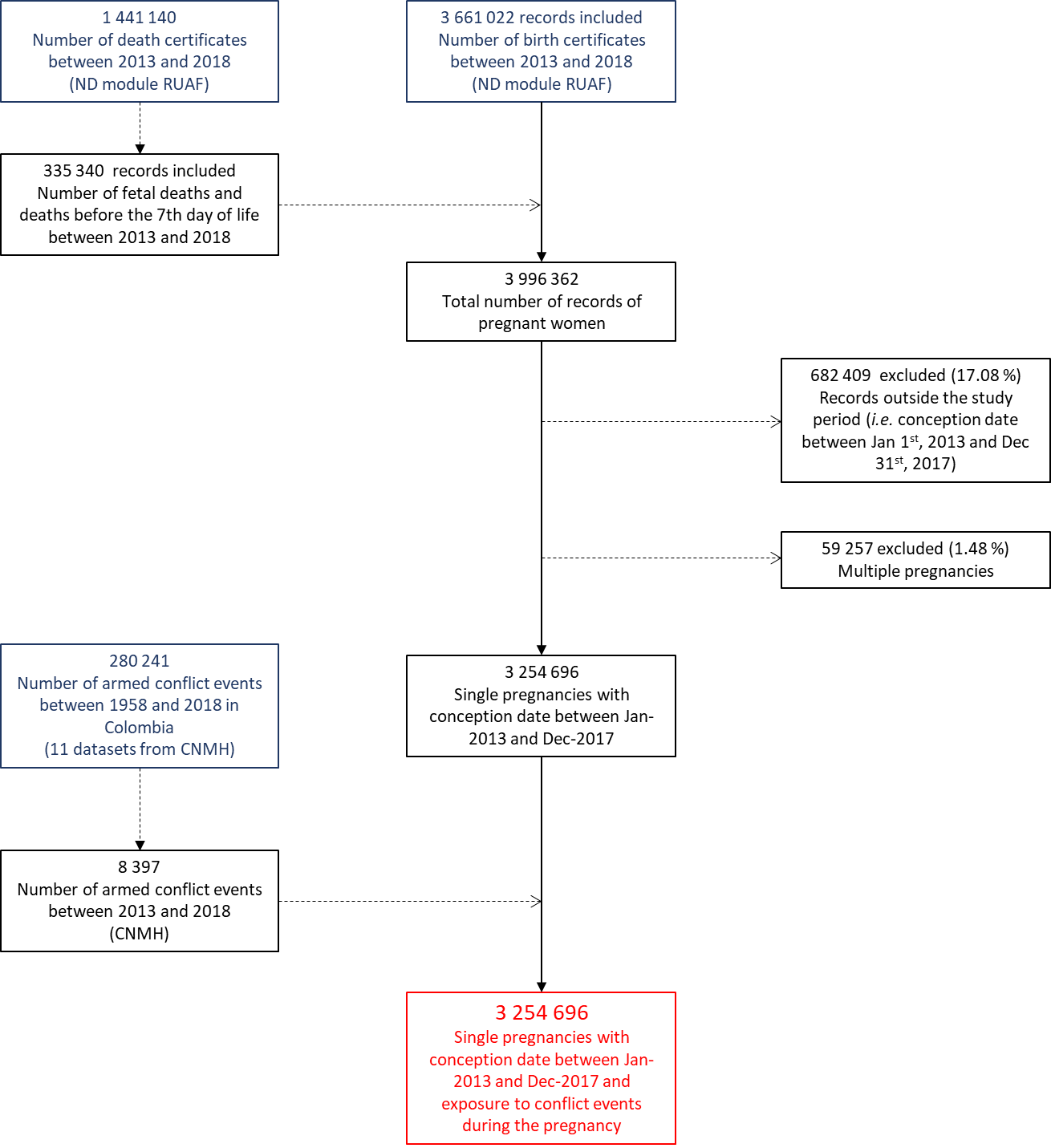


1. **Context of the armed conflict in Colombia and peace process**

Figure B shows a timeline of ceasefires declared during the Havana talks between the Colombian Government and FARC. Each letter identifies a ceasefire. In our analyses we evaluated the effects of ceasefires F (July 20^th^, 2015) and G (August 29^th^, 2016) on the number of conflict events. After estimating the effect of ceasefires on exposure to conflict events, in our main analysis we evaluated the effect of ceasefire F on our main outcomes (stillbirths and perinatal mortality), because we did not observe any significant effect of ceasefire G on the number of conflict events (see below).

The characteristics of each ceasefire were:

1. First ceasefire declared by FARC, which was temporary and unilateral, between November 20, 2012 and January 20, 2013.
2. Second ceasefire declared by FARC, which was temporary and unilateral, between December 15, 2013 and January 15, 2014.
3. Third ceasefire declared by FARC, which was temporary and unilateral, between May 20, 2014 and May 28, 2014.
4. Fourth ceasefire declared by FARC, which was temporary and unilateral between June 9, 2014 and June 30, 2014.
5. Fifth ceasefire declared by FARC, which was declared as indefinite and unilateral, between December 20, 2014 and May 22, 2015. It was suspended after a bombing by the Colombian army.
6. Sixth ceasefire declared by FARC, which was indefinite and unilateral since July 20, 2015 lasting until the signing of the peace accord.
7. Seventh ceasefire, declared by the Colombian Government, which was indefinite and bilateral since August 29, 2016 lasting until the signing of the peace accord.

Figure B. Ceasefires declared during the Havana talks


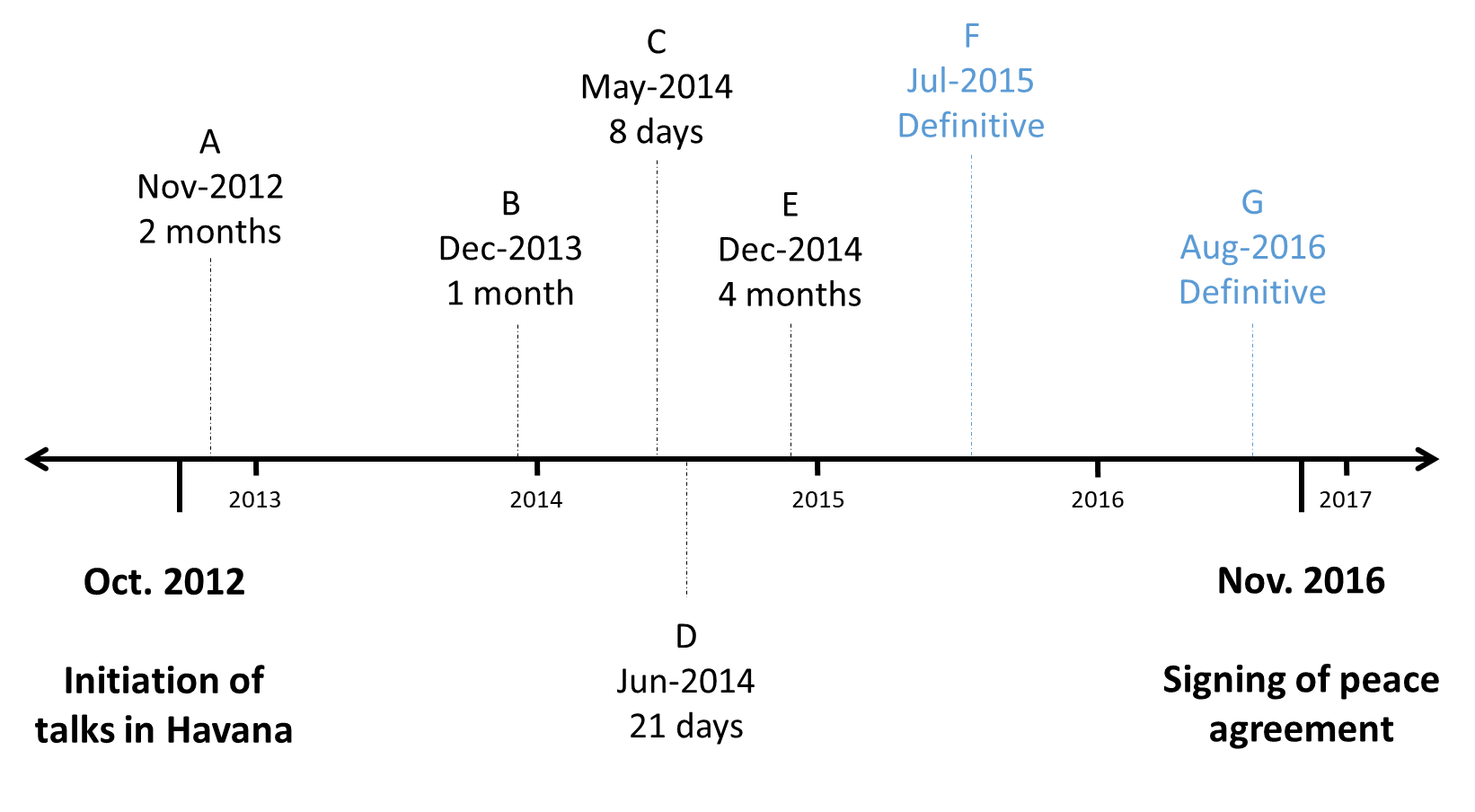


Figure C shows the geographical areas of operation of armed groups in Colombia between 2000 and 2017. FARC*: Fuerzas Armadas Revolucionarias de Colombia* (guerrilla group). ELN: *Ejército de Liberación Nacional* (guerrilla group). AUC: *Autodefensas Unidas de Colombia* (paramilitary group). Colors indicate ranges of the number of conflict events involving each of these armed groups, reported in municipalities during the period between 2000 and 2017.

Figure C. Historic presence of armed groups in Colombia (2000-2017)


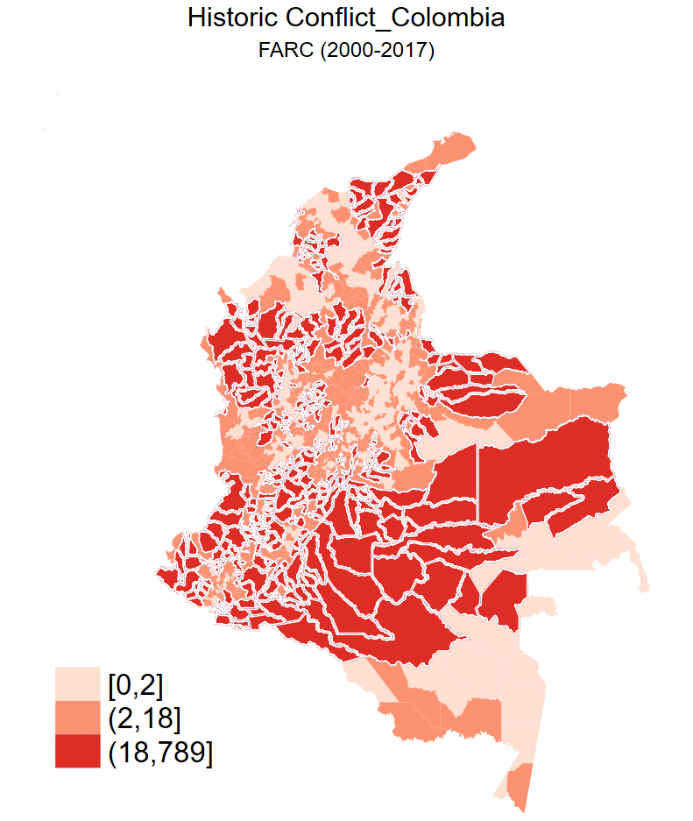

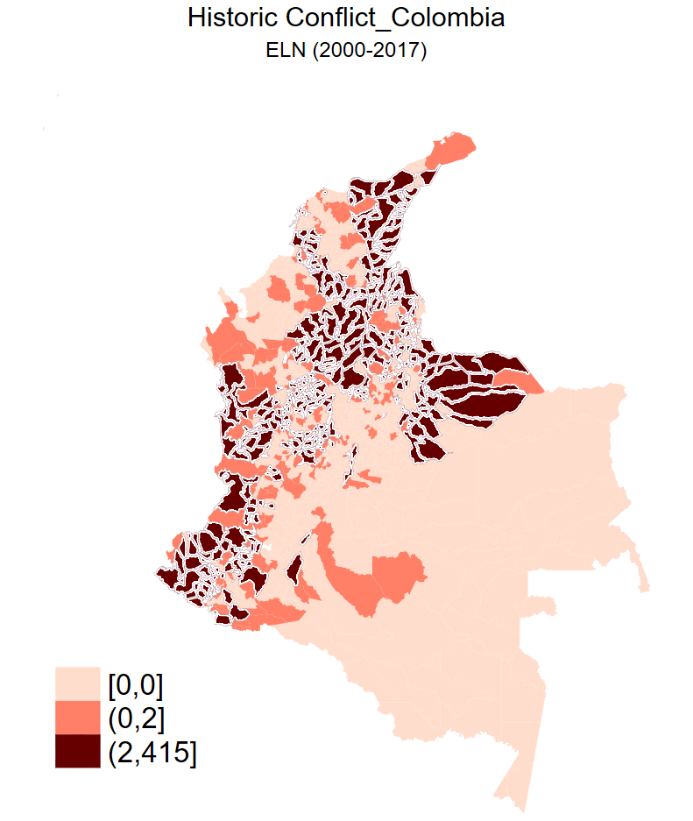

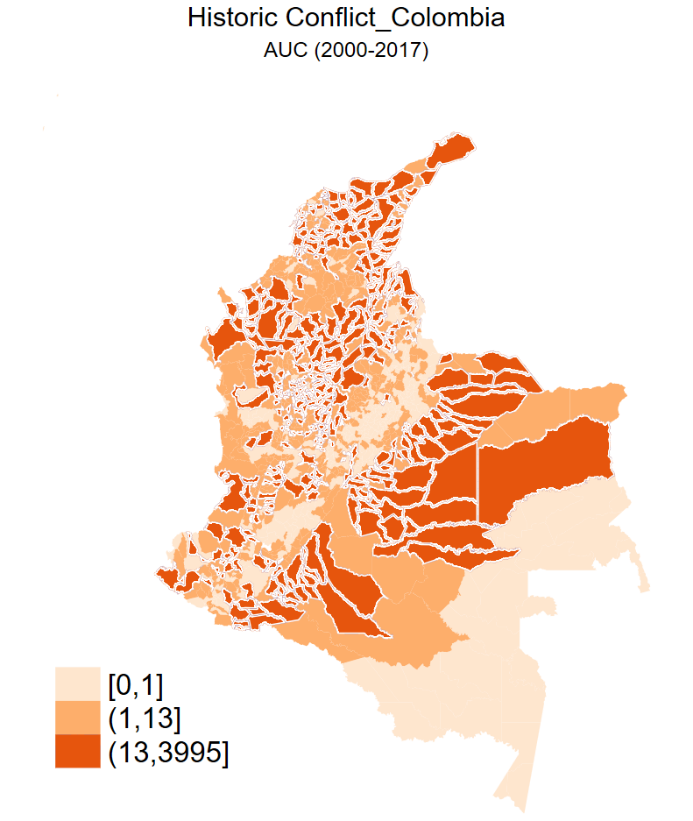


Figure D shows the trend of conflict armed events to which pregnant women who started their pregnancy between January 1^st^, 2013 and December 31^st^, 2017 were exposed during pregnancy. Each dot represents a cohort of pregnancies defined by conception dates in the same month. The dot value is the average of conflict events to which the cohort of women was exposed during pregnancy. The red line represents the July 20^th^, 2015 ceasefire. The blue line represents the 29^th^, August 2016 ceasefire.

Figure D. Trend in the number of conflict events to which pregnant women were exposed during pregnancy in Colombia between Jan-2013 and Dec-2017


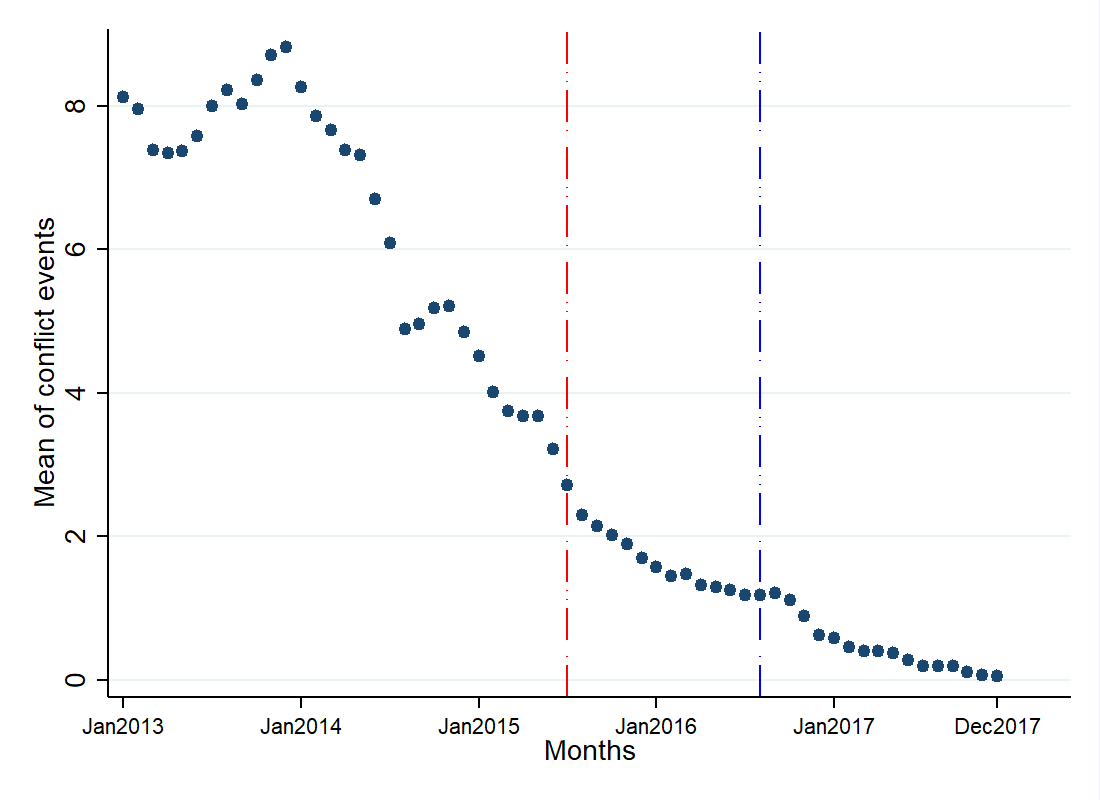


- 1. Definitions of types of armed conflict events

Below we present the definitions of all types of armed conflict events provided in the National Center for Historical Memory - CNMH dataset (see <http://micrositios.centrodememoriahistorica.gov.co/observatorio/sievcac/categorias/>).

Terrorist attack

Any attack perpetrated through the use of explosives, which occur in densely populated areas and in which there is a plurality of effects on people or civilian targets, regardless of whether the objective of the action is civil or military.

Act of war

Acts that are carried out as legitimate actions within a war context, in that these respond to a defined military objective and make use of lawful combat means and weapons. At least two parties are involved in the military actions, be them the government or state armed forces and organized armed groups, which under the direction of a higher command are those directly conducting hostilities or organized armed activities.

Attack on populations

An incursion by an armed group that implies the temporary occupation of a territory and a continuous military action directed towards the razing of a military objective within an urban area or population center, and that affects civilian populations.

Selective murder

The intentional homicide of three or fewer people in a defenseless state and in the same circumstances of manner, time, and place, perpetrated by the actors of the armed conflict or with their participation.

Kidnapping

The seizure, subtraction, retention, or concealment of a person, against their will, through intimidation, violence, or deception, by the actors of the armed conflict or with their participation. This can be ‘simple’ when it does not have a manifest purpose, or ‘extortionary’ when it is carried out with the purpose of demanding a profit or any reward in exchange for the captive’s freedom, or for something to be done or omitted, or for publicity or political purposes (Congress of the Republic, 2000).

Child recruitment

In the context and course of the armed conflict, an episode whereby children and adolescents (under 18 years of age) are recruited, used, or forced to participate directly or indirectly in hostilities or in armed actions (Article 162 of Law 599 of 2000).

Massacre

The intentional homicide of four or more people in a defenseless state and in the same circumstances of manner, time, and place, and that is distinctive by the public exposure of the violence and the asymmetric relationship between the armed actor and the civilian victims, without interaction with other groups of armed actors.

Forced disappearance

The subjection of a person to deprivation of his liberty, against his will, through any manner including arrest, detention, kidnapping or hostage-taking, by agents of the State, members of illegal armed groups that take part in the armed conflict, or with their authorization, support, or acquiescence, followed by the person’s concealment and/or the refusal to acknowledge said deprivation or to give information about their whereabouts, removing the person from the protection of the law.

Damage to property

Damages, total and partial, caused to material goods that are not military objectives and that should not be the object of attack or reprisal by the actors of the armed conflict.

Sexual violence

All acts of a sexual nature perpetrated by one or more of the actors in the armed conflict, on people put in a defenseless state and whose will is subdued, not only through physical force but also by various forms of coercion and deception. These acts may be carried out, among other reasons, with the intention of educating communities, controlling women's bodies, punishing other members of armed groups directly or through family networks, legitimizing particular ways of exercising sexuality, and punishing those identities and practices that violate the order established by the members of the armed groups.

Landmine

It refers to events involving Antipersonnel Mines (MAP), Unexploded Munitions (Muse), and/or Improvised Explosive Devices (AEI), hereinafter mine events. The set of attacks, damages, and incidents that occurred and that caused damage or that had the potential to do so. Attacks are events caused by MAP and AEI, which caused physical and/or psychological harm to one or more people; while the effects are the events that occurred by Muse. The incidents (or hazards) are mine-related events (MAP, Muse, and AEI), which have the potential to materialize as attacks and cause damage, including seizures and the deactivation of minefields. MAP is understood to be any mine designed to explode due to the presence, proximity, or contact of a person, and in the event that this occurs, it has the potential to incapacitate, injure and/or kill one or more persons. A Muse is an explosive ammunition that has been loaded, its fuse placed, armed or, on the contrary, prepared for its use or already used, and that although it may have been fired, thrown, thrown, or projected, it remains unexploded due to malfunction, for the type of design or other reason. AEIs are handcrafted antipersonnel mines.

1. **Regression discontinuity design**

We used regression discontinuity (RD), a quasi-experimental study design, to permit causal inference in the absence of treatment randomization. In this design, the conflict violence exposure (“treatment”) status of each individual is determined by a threshold of a continuous variable. This assignment rule generates a discontinuity in the probability of exposure, and under certain conditions this assignment is as good as random for individuals near to the threshold. Therefore, this analytical design can identify the causal effect of exposure for these individuals.^1,2^ Exposure assignment following such a rule may be either deterministic (there is a deterministic change of the probability of being exposed) or nondeterministic (the probability of being exposed is higher on one side of the threshold than on the other side). The approach in the first case is called sharp RD and, in the second, fuzzy RD.

In our study, the July 20^th^, 2015 ceasefire declared by FARC generated a discontinuous decrease in the number of conflict events to which women who started their pregnancy just after the ceasefire were exposed. Thus, we used the July 20^th^, 2015 ceasefire as a threshold of the RD design to estimate the causal effect of a decrease in exposure to armed conflict events during pregnancy on the risks of miscarriage, stillbirth, and perinatal mortality.

Following Moscoe et al (2015) and the potential outcome framework,^3^ the average causal effect (ACE) in the sharp RD (SRD) design is defined as:

$${ACE}_{SRD}=\lim_{z\uparrow c} E\left[ Y_{i}\left( 1 \right)|Z_{i}=z \right]-\lim_{z\downarrow c} E\left[ Y_{i}\left( 0 \right)|Z_{i}=z \right],$$

(1)

where $Y_{i}\left( 1 \right)$ is the outcome (miscarriage, stillbirth, or perinatal mortality) for individual $i$, when individual $i$ is exposed (i.e. the woman began her pregnancy after the ceasefire); $Y_{i}\left( 0 \right)$ is the outcome for individual $i$ when she is unexposed (i.e. the woman began her pregnancy before the ceasefire); and $Z$ is the continuous variable (i.e. conception date) that represents the threshold or cut-off point $c$ from which the probability of being exposed changes discontinuously for individuals. In our study, similarly to a randomized experiment with imperfect adherence, exposure to conflict took a nondeterministic form (women exposed and not exposed to conflict were on both sides of the threshold); therefore, our study approach was a fuzzy RD. In this case, the ${ACE}_{SRD}$ is equivalent to the intention-to-treat effect (ITT) in a fuzzy RD. This was our parameter of interest.

To estimate the ITT effect, we used the following equation:

$Y=\beta_{0}+\beta_{1}T+f\left( Z-c \right)+\varepsilon$,

(2)

where $c-h\leq Z\leq c+h$;

where $h$ is a window on both sides of the threshold (in our study, $bandwidth=2h$); $T=1$ if the woman’s conception date $Z$ was after threshold $c$ (i.e. the ceasefire), or $T=0$ otherwise; $\beta_{1}$is the ITT effect; and $f\left( . \right)$ is a chosen functional form for $Z$. As in a randomized experiment, if baseline characteristics exhibit a continuous distribution around the RD threshold (that is, there are no imbalances in the distribution of these characteristics for observations at either side of the bandwidth around the threshold), it is not necessary to include baseline covariates in the RD regressions to obtain consistent estimates of the treatment effect.^1,4,6^

As demonstrated by Hahn, Todd, and van der Klaauw (2001), if $f\left( . \right)$ is a nonparametric function of first order (i.e. a local linear regression), then (2) will be a consistent estimator of the ITT parameter.^4^ Our main results are based on local linear regression with optimal bandwidths proposed by Imbens and Kalyaraman.^5^ Additionally, we used several alternative bandwidths and parametric regression with first-, second-, and third-order polynomial specifications to assess the robustness of our main results.^6^

1. **The effects of ceasefires on exposure to conflict events**

Figure E shows regression discontinuity plots with the intention-to-treat (ITT) effects of the July 20th, 2015 and August 28th, 2016 ceasefires on exposure to FARC-related conflict events during pregnancy. The ITT effect was estimated by regression discontinuity analysis using local linear regression (LLR) and bandwidths of 56 days. A, B, C and D show the effects of the July 20th, 2015 ceasefire (red lines). E, F, G and H show the effects of the August 28th, 2016 ceasefire (blue lines). A and E include women from all municipalities in Colombia. B and F only include women in M-p90 municipalities. C and G only include women in M-p75 municipalities. D and F only include women in M-other municipalities. We do not show graphs for women in M-zero municipalities because these women were not exposed to conflict events. The results indicate that the July 20th, 2015 ceasefire produced a significant discontinuity in the number of FARC-related conflict events only for women in M-p90 and M-p75 municipalities, while the August 28th, 2016 ceasefire did not result in any statistically significant changes in exposure to conflict events for pregnant women. We use different scales on the Y-axis for more clear visualization of the estimated effects of the ceasefires, as the number of conflict events close to the August 2016 ceasefire was much lower than for the previous ceasefire.

Figure E. Effects of the July 20th, 2015 and August 28th, 2016 ceasefires on the exposure to FARC-related conflict events during pregnancy: Colombia and categories of municipalities (RD plots)

A E


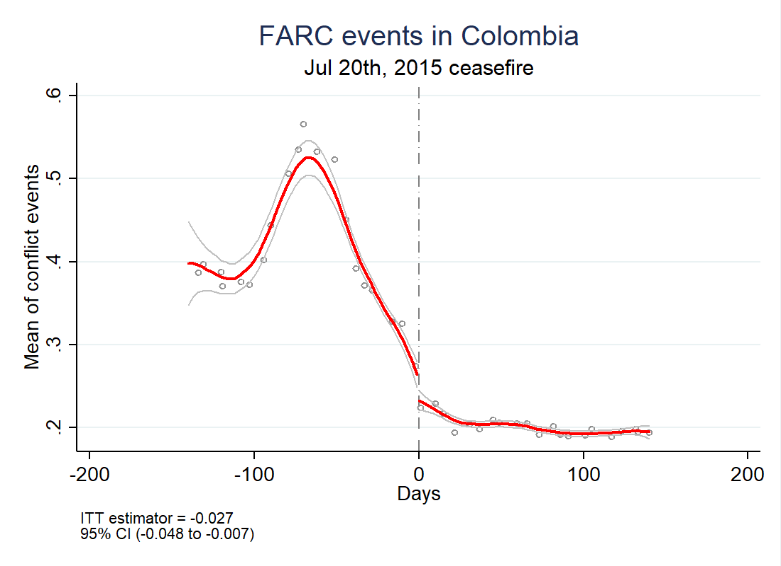

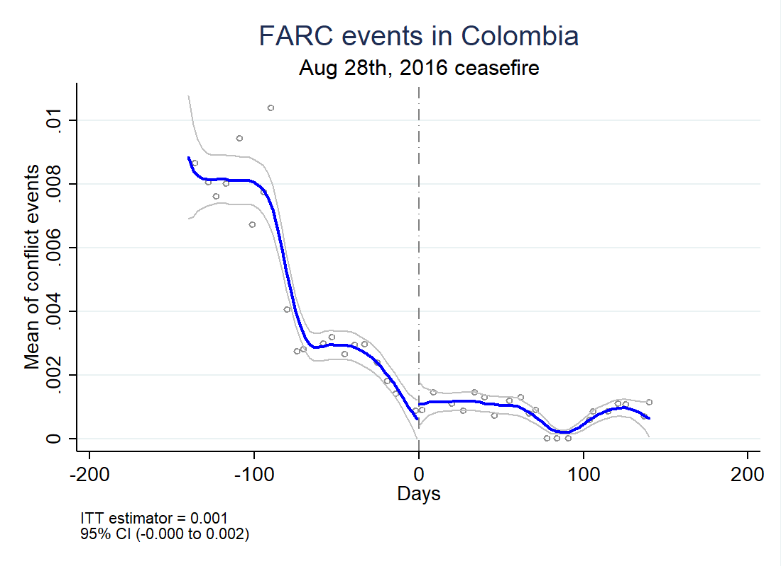


B F


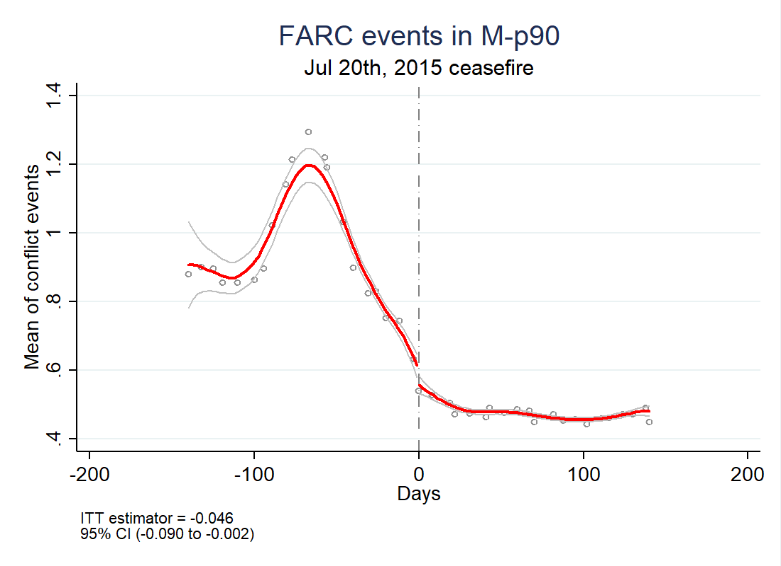

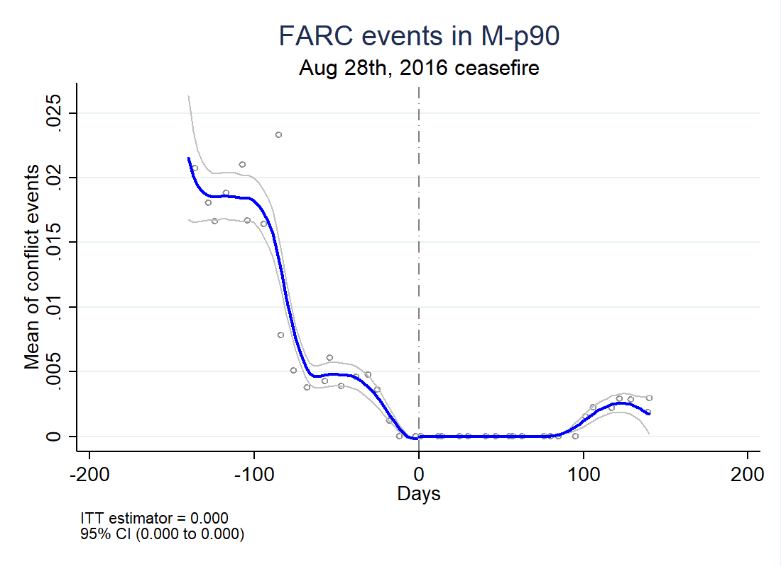


Figure E (Continued)

C G


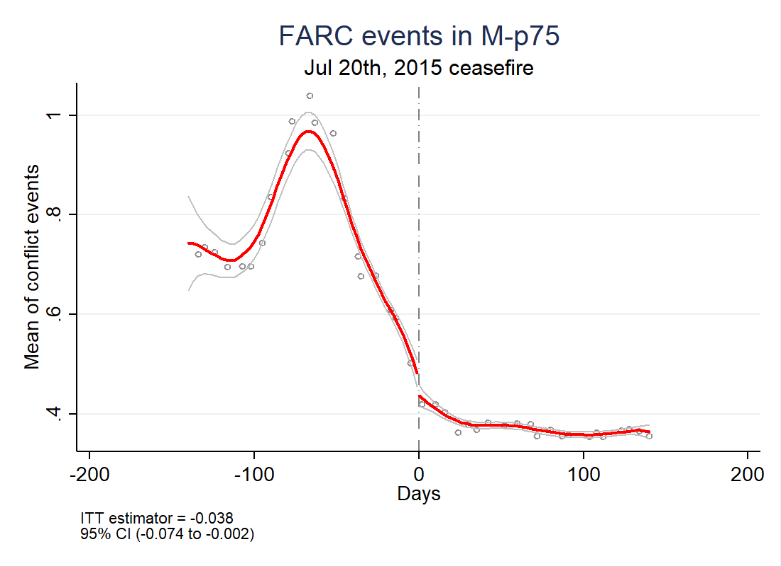

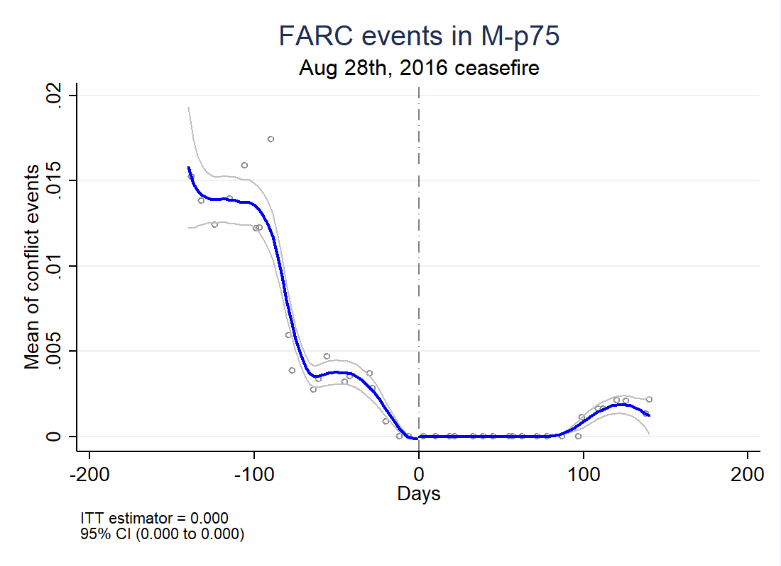


D H


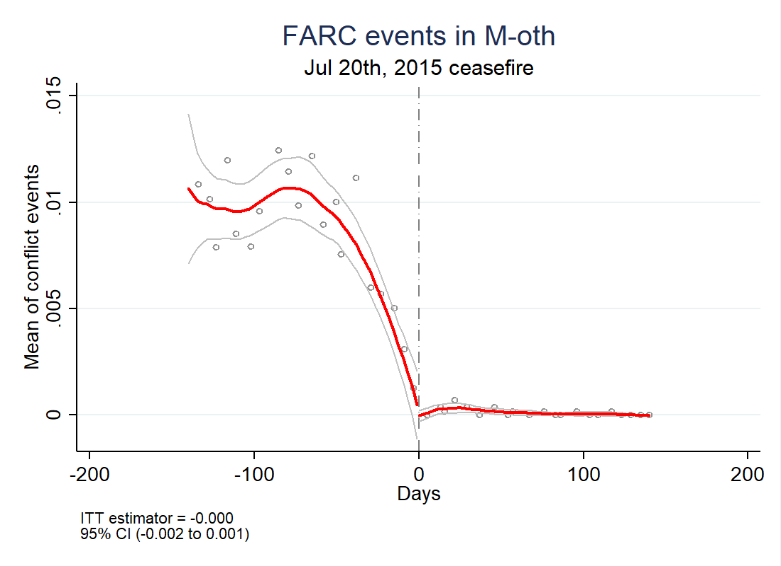

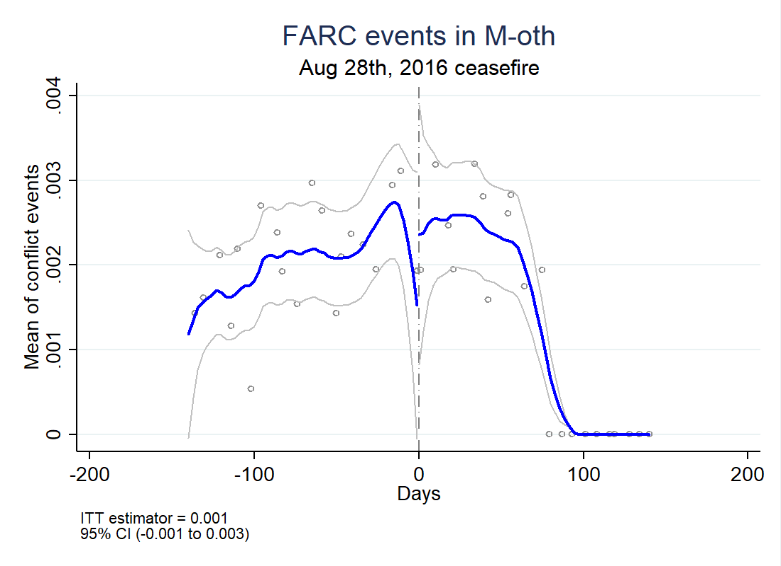


1. **Analyses of the validity of RD assumptions**

We assessed the validity of our causal RD estimates by formally testing the two main RD design assumptions: no-manipulation and continuity of baseline variables around the threshold. In an RD setting, manipulation refers to the possibility of individuals determining the continuous running variable – in our case, the conception date – to purposely alter their individual probability of being exposed to the treatment (conflict events). As we explain in the paper, FARC were involved in other five ceasefires during the Havana talks prior to the July 20th, 2015 ceasefire, all of which ended up being very limited in duration (lasting no more than four months) with violence returning to the municipalities soon after these ceasefires ended. There was no reason for Colombians to trust that the July 2015 ceasefire was going to be any more long-lived than previous ceasefires, including due to a long list of other unsuccessful, similarly short-lived FARC ceasefires that took place before the Havana talks. Therefore, it does not seem plausible that women would decide to delay their conception date to wait for the declaration of the July 2015 ceasefire. Despite this implausibility, we can still test for the possibility of manipulation of conception dates around the July 2015 ceasefire threshold with our data, since the no-manipulation assumption implies that the distribution of pregnant women must be continuous in the neighborhood of the threshold. In the event, as we expected due to the history of the Colombian conflict and peace processes, we find no evidence of manipulation. The results from the no-manipulation tests are summarized in the paper and presented in detail below.

- 1. No-manipulation assumption

Figure F shows the tests of the no-manipulation assumption, i.e. that the conception date is continuous near the

July 20th, 2015 ceasefire (threshold). Panel A shows the histograms of the distribution of women according to their conception dates, around the July 20th, 2015 ceasefire, for women in M-p90 and M-p75 municipalities. Panel B shows graphs for the test of the density of conception date observations proposed by McCrary (2008), to assess the no-manipulation assumption.^7^ Both panels indicate no evidence of manipulation of conception dates: there is no systematic discontinuity in the distribution of pregnant women around the July 20th, 2015 ceasefire, in either M-p90 (Discontinuity estimate= -0.030 95% CI [-0.072 to 0.013]) or M-p75 municipalities (Discontinuity estimate= -0.030 95% CI [-0.067 to 0.007]).

Figure F. Tests of the RD no-manipulation assumption around the July 20th, 2015 ceasefire threshold

A.


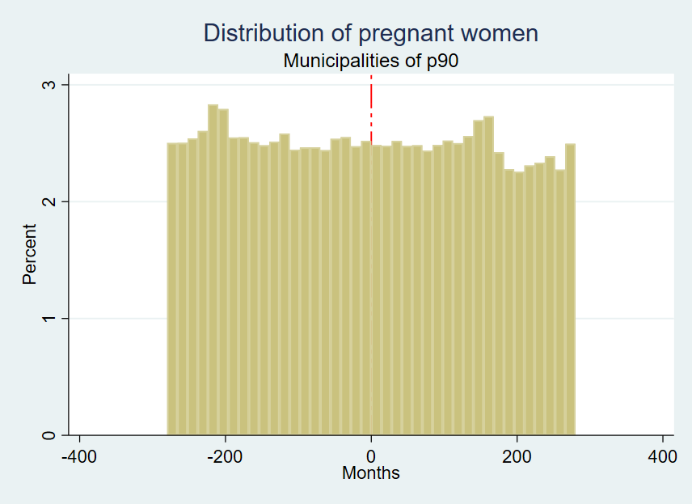

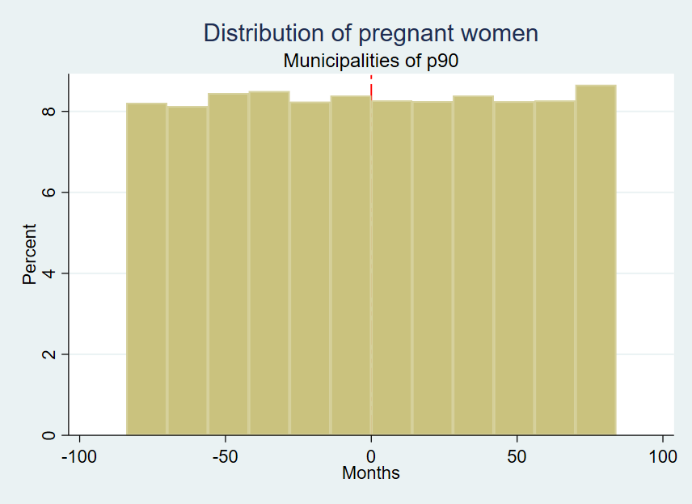

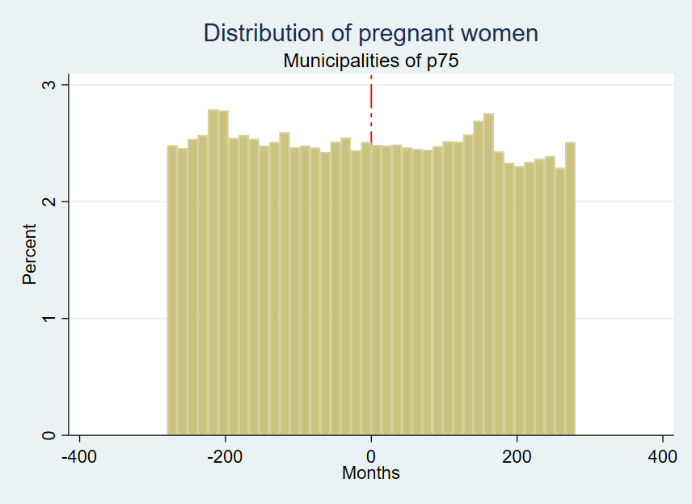

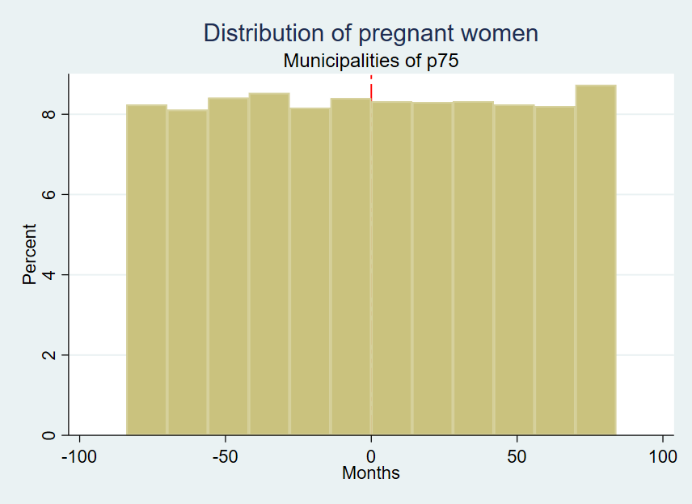


B.


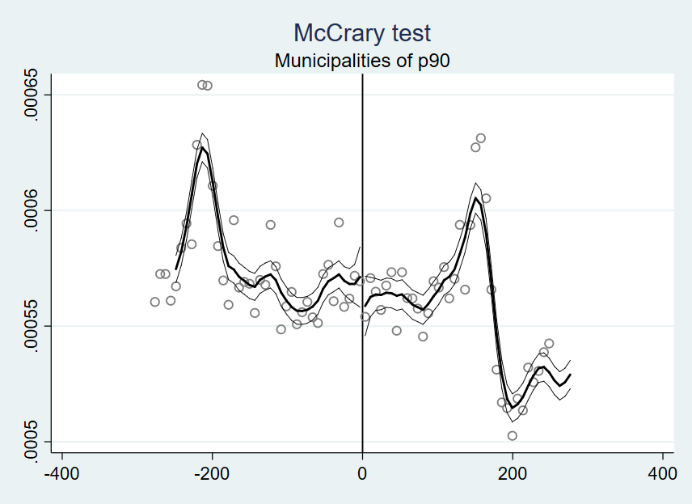

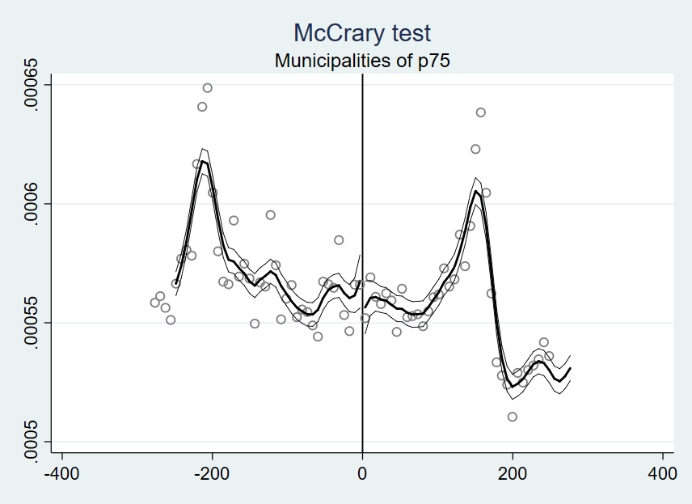


- 1. Continuity of baseline characteristics assumption

The identification of causal effects in our study hinges on the assumption that the pregnant women with conception dates just to left and right of the July 20^th^, 2015 ceasefire are similar in their characteristics. This can be assessed by checking statistically for differences in observable baseline characteristics (i.e. possible confounders). Figures G and H (for women in M-p90 and M-p75 municipalities, respectively) show plots with the results of regression discontinuity analyses for each variable presented in Table 1 in the main text. For each baseline characteristic as a separate outcome, we estimated the intention-to-treat (ITT) effect using local linear regression (LLR) and 28 days’ bandwidth, as in the case of our main study outcomes (Fig 3, main text). The estimation results show no evidence of discontinuity in any of the baseline characteristics.

Figure G. Tests of balance in baseline characteristics around the July 20^th^, 2015 ceasefire for women in M-p90 municipalities


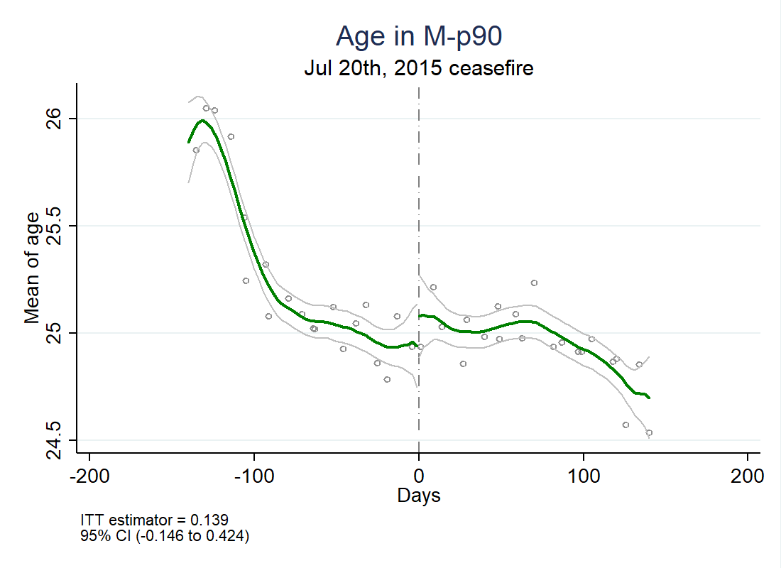

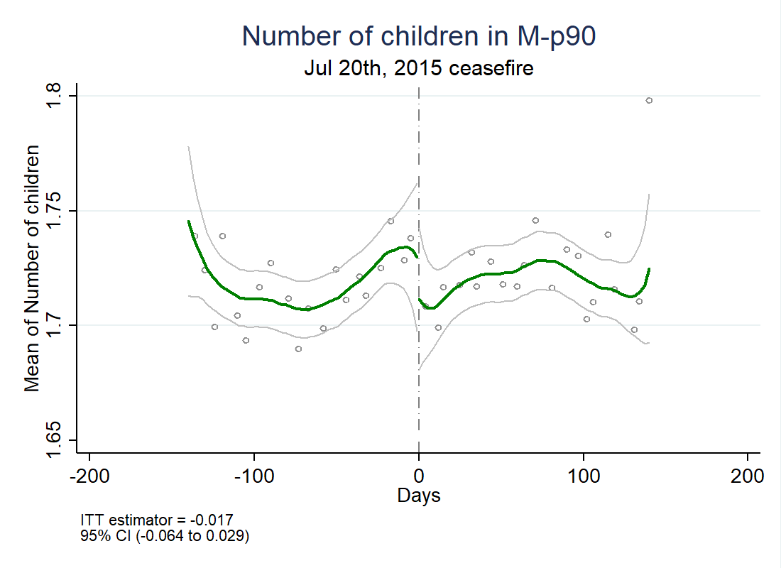

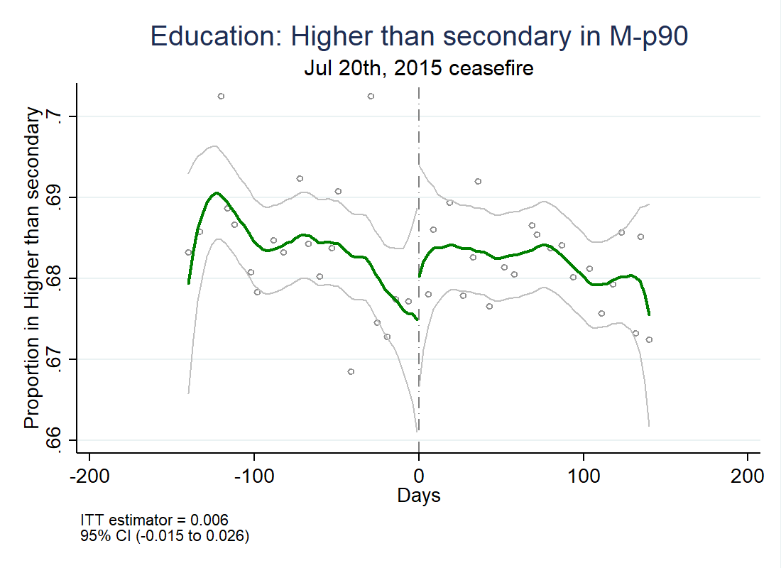

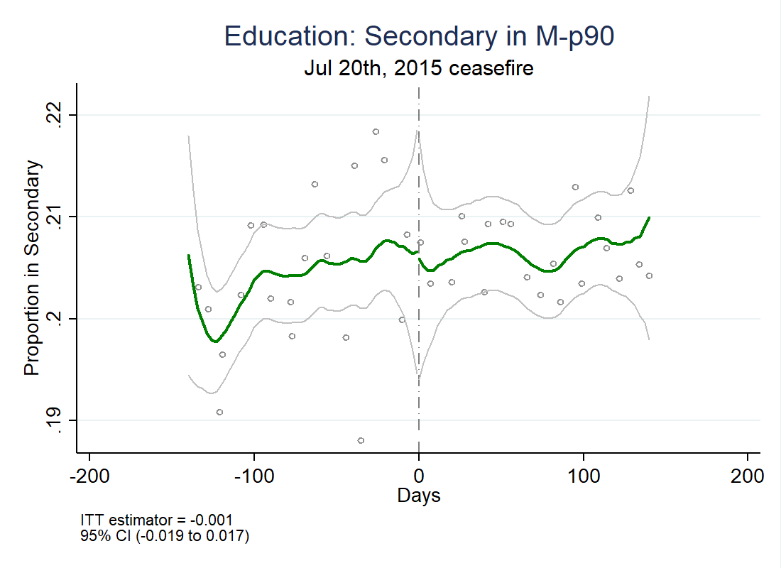


Figure G (Continued)


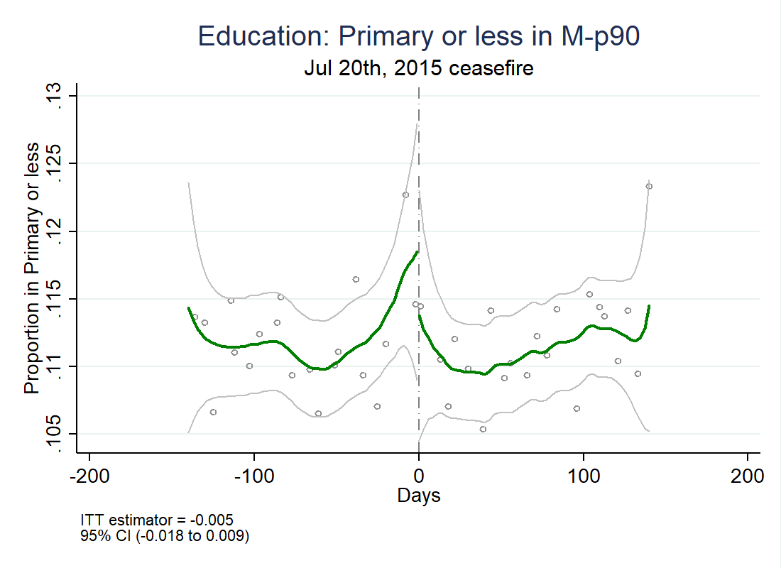

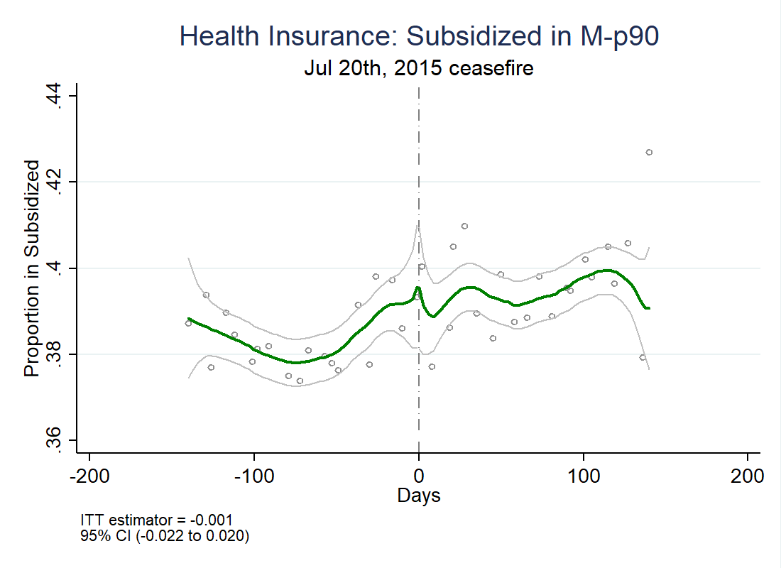

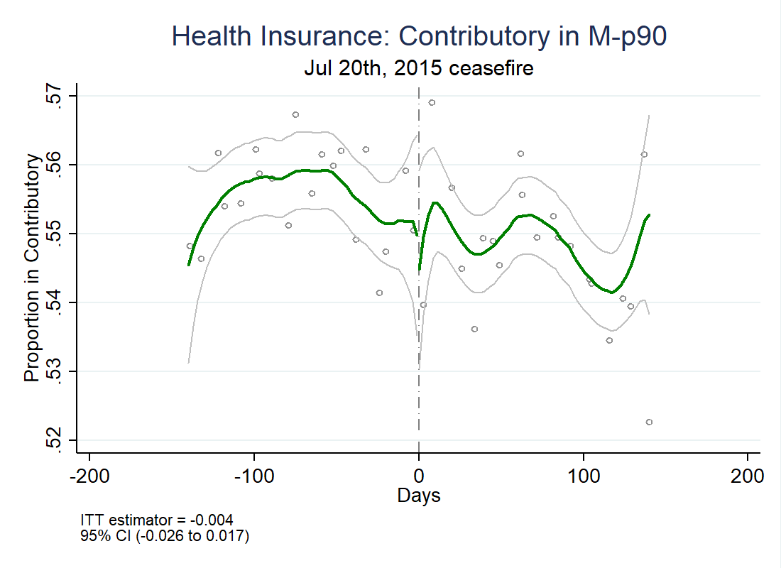

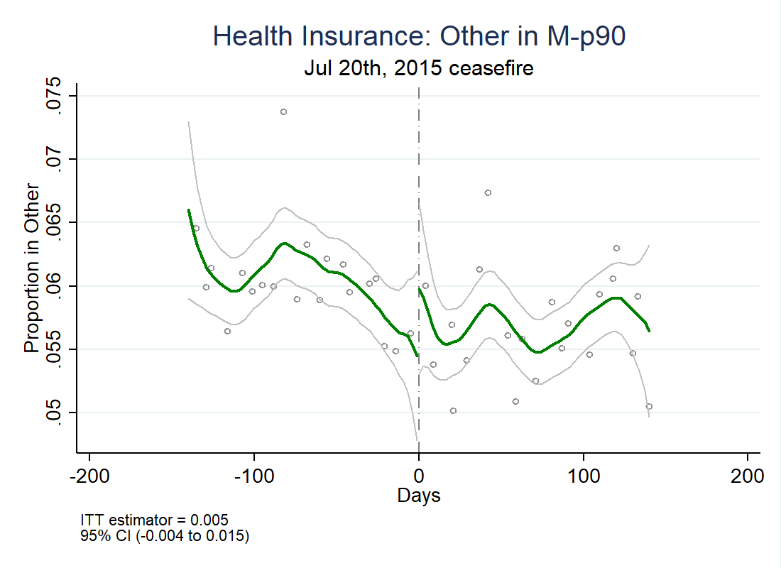

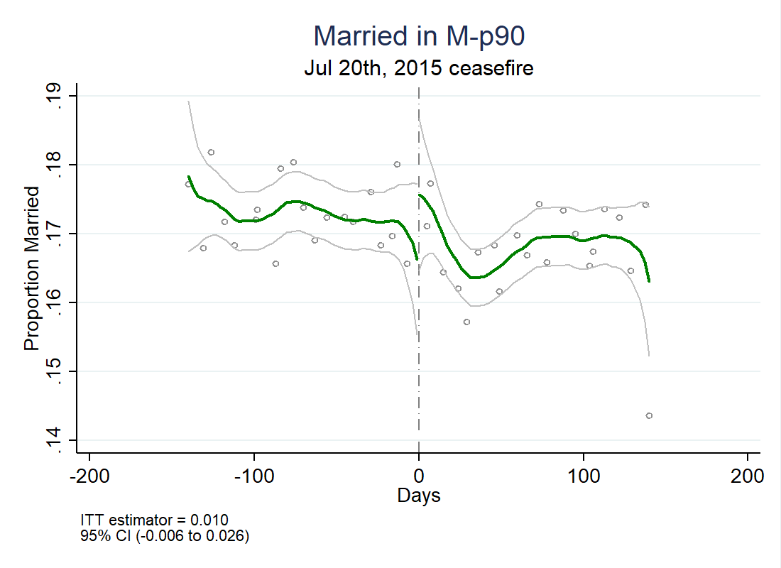


Figure H. Tests of balance in baseline characteristics around the July 20^th^, 2015 ceasefire for women in M-p75 municipalities


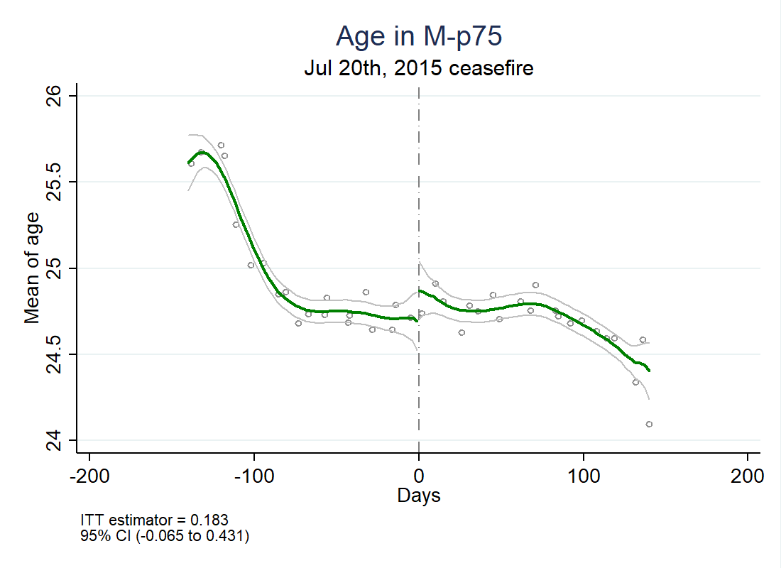

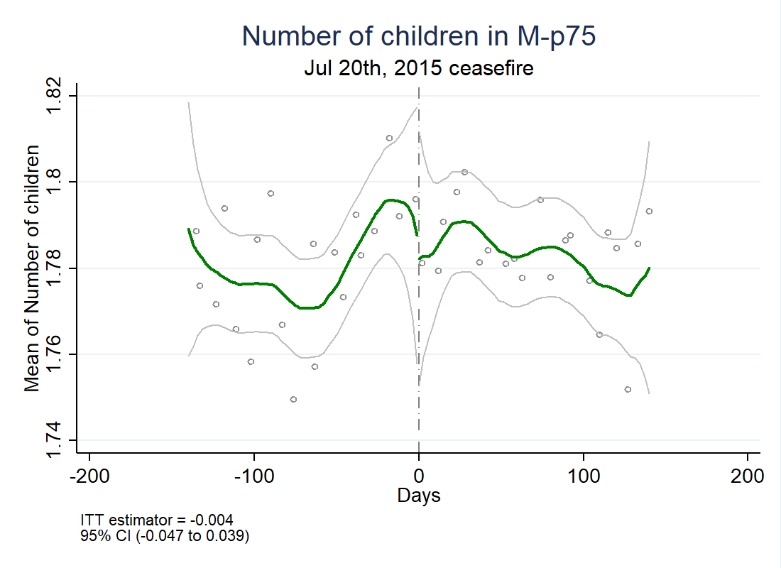

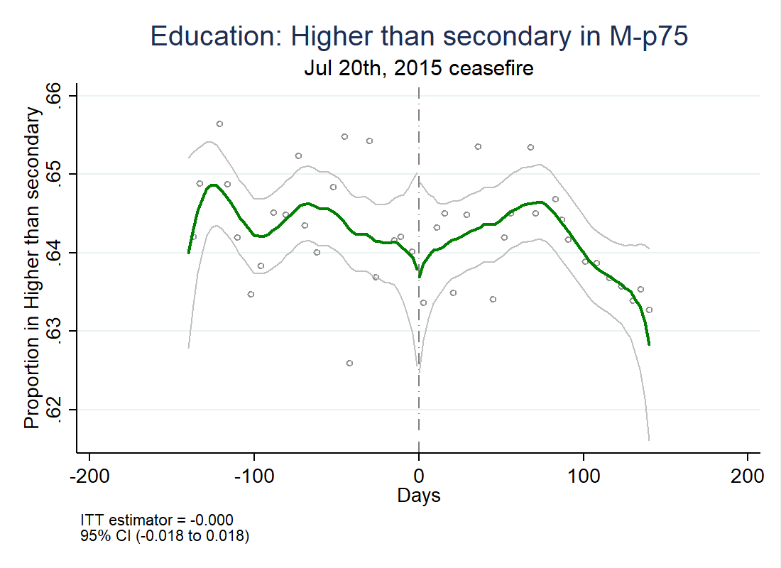

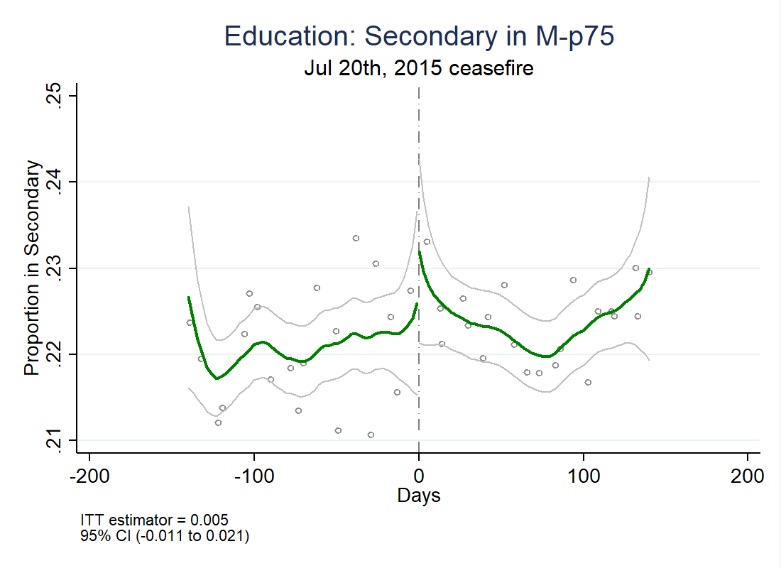

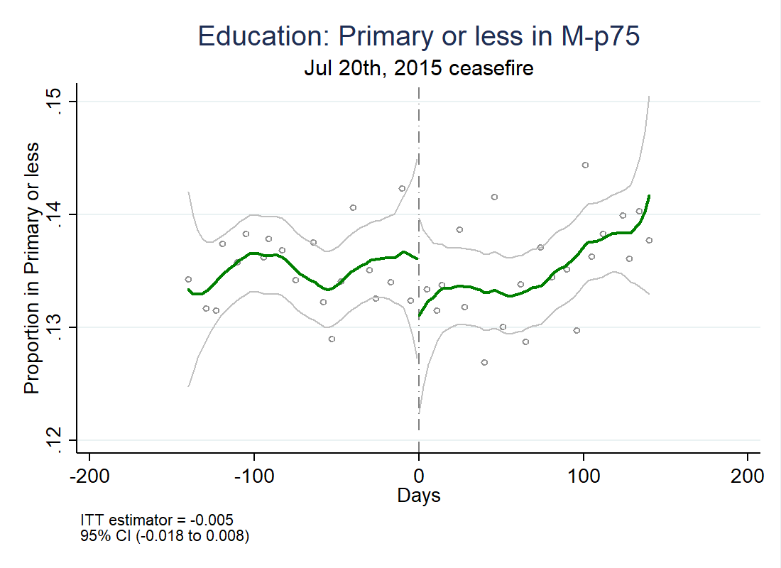

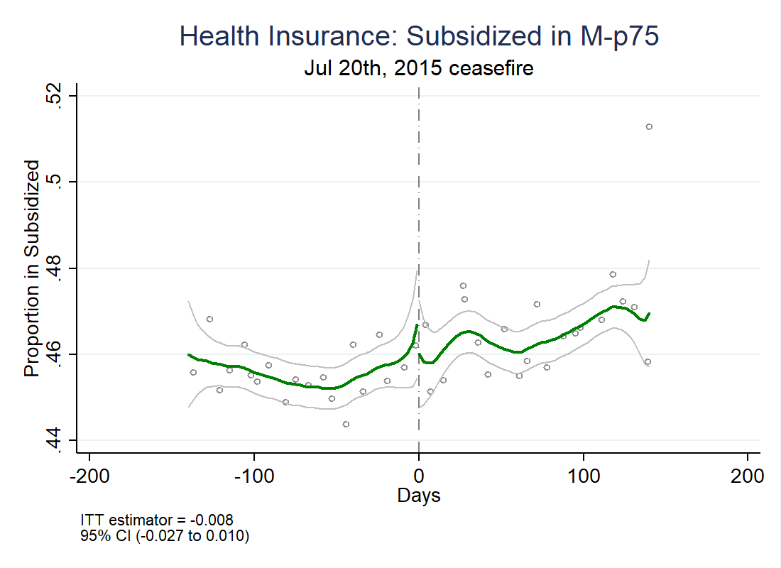


Figure H (Continued)


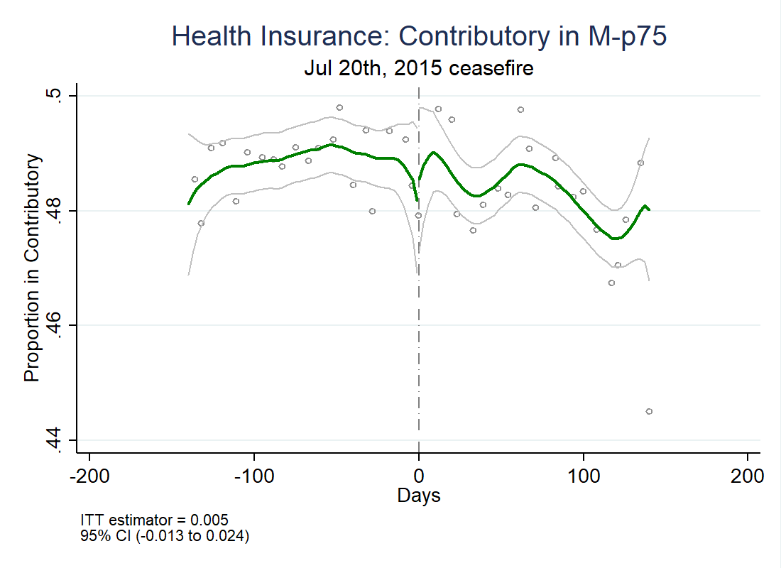

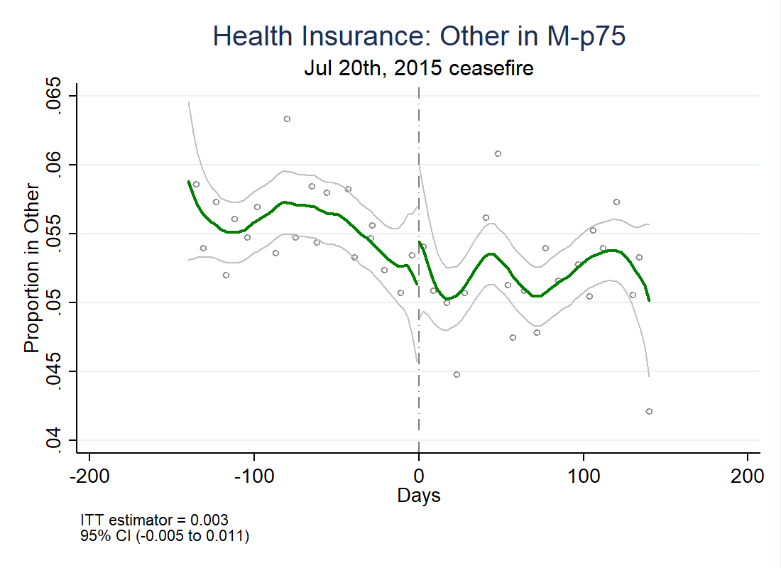

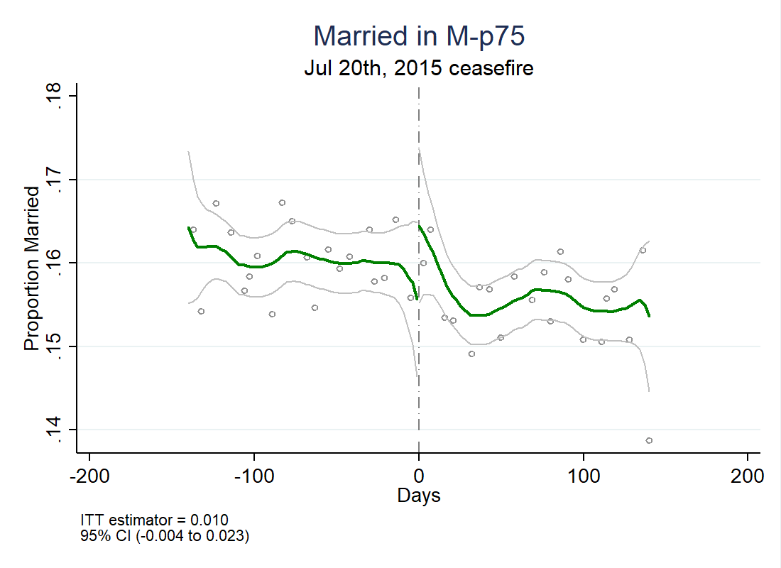


1. **Robustness checks**

We conducted a battery of robustness analyses and falsification tests for our main results. Our main results (Fig 3 in the main text) are presented in Table A. We used regression discontinuity analysis to determine the effect of the July 20th, 2015 ceasefire on fetal deaths and perinatal mortality. This analysis used local linear regression (LLR) and bandwidth of 28 days. Column *Left mean* shows the estimated mortality rate per 1000 pregnancies just before the ceasefire date (i.e. left side of the threshold). The intention-to-treat (ITT) effect measures the absolute difference in mortality risk.

Table A. Effects of the July 20th, 2015 ceasefire on fetal deaths and perinatal mortality

|  | Left mean | ITT effect | 95% CI | p value | Functional form | Bandwidth | Observations in bandwidth |
| --- | --- | --- | --- | --- | --- | --- | --- |
|  |  |  |  |  |  |  |  |
| M-p90 municipalities |  |  |  |  |  |  |  |
|  |  |  |  |  |  |  |  |
| Miscarriage | 72.11 | 8.52 | -7.47 to 24.52 | 0.30 | LLR | 28 | 19 679 |
| Stillbirth | 15.29 | -9.53** | -16.13 to -2.93 | 0.00 | LLR | 28 | 18 195 |
| Perinatal mortality | 19.91 | -10.69** | -18.32 to -3.05 | 0.01 | LLR | 28 | 18 195 |
|  |  |  |  |  |  |  |  |
|  |  |  |  |  |  |  |  |
| M-p75 municipalities |  |  |  |  |  |  |  |
|  |  |  |  |  |  |  |  |
| Miscarriage | 65.61 | 3.27 | -9.78 to 16.32 | 0.62 | LLR | 28 | 26 281 |
| Stillbirth | 13.74 | -7.57** | -13.14 to -2.00 | 0.01 | LLR | 28 | 24 578 |
| Perinatal mortality | 17.07 | -6.86* | -13.24 to -0.48 | 0.04 | LLR | 28 | 24 578 |
|  |  |  |  |  |  |  |  |

** p<0.01, * p<0.05

ITT: Intention-to-treat

95% CI: 95% Confidence Interval

Bandwidth: number of days between upper and lower limit of the bandwidth

- 1. RD specification changes to estimate the effect of the July 20th, 2015 ceasefire on fetal deaths and perinatal mortality

We re-ran all our RD estimations using alternative bandwidths, nonparametric (local linear regression, LLR) and parametric approaches with first-, second-, and third-order polynomial specifications. Tables B and C show RD estimation results for women in M-p90 and M-p75 municipalities, respectively.

Table B. Effects of the July 20th, 2015 ceasefire on fetal deaths and perinatal mortality. LLR and parametric regressions by order of polynomial for M-p90 municipalities

|  | ITT effect | 95% CI | p value | Functional form | Bandwidth | Observations in bandwidth |
| --- | --- | --- | --- | --- | --- | --- |
|  |  |  |  |  |  |  |
| Stillbirth | -15.83** | -26.62 to -5.04 | 0.00 | LLR | 14 | 8 652 |
| Stillbirth | -14.21** | -23.92 to -4.50 | 0.00 | Linear | 14 | 8 652 |
| Stillbirth | -20.76* | -38.99 to -2.53 | 0.03 | Quadratic | 14 | 8 652 |
| Stillbirth | -31.40 | -67.16 to 4.36 | 0.09 | Cubic | 14 | 8 652 |
| Stillbirth | -9.53** | -16.13 to -2.93 | 0.00 | LLR | 28 | 18 195 |
| Stillbirth | -7.85** | -13.73 to -1.96 | 0.01 | Linear | 28 | 18 195 |
| Stillbirth | -13.00* | -23.02 to -2.97 | 0.01 | Quadratic | 28 | 18 195 |
| Stillbirth | -21.32** | -37.06 to -5.58 | 0.01 | Cubic | 28 | 18 195 |
| Stillbirth | -6.52* | -11.70 to -1.33 | 0.01 | LLR | 42 | 27 698 |
| Stillbirth | -4.31 | -9.03 to 0.41 | 0.07 | Linear | 42 | 27 698 |
| Stillbirth | -10.73** | -18.24 to -3.21 | 0.01 | Quadratic | 42 | 27 698 |
| Stillbirth | -15.69** | -26.99 to -4.38 | 0.01 | Cubic | 42 | 27 698 |
| Stillbirth | -4.88* | -9.28 to -0.48 | 0.03 | LLR | 56 | 36 911 |
| Stillbirth | -3.59 | -7.55 to 0.36 | 0.08 | Linear | 56 | 36 911 |
| Stillbirth | -7.07* | -13.34 to -0.80 | 0.03 | Quadratic | 56 | 36 911 |
| Stillbirth | -13.93** | -22.92 to -4.93 | 0.00 | Cubic | 56 | 36 911 |
|  |  |  |  |  |  |  |
| Perinatal mortality | -15.99** | -28.02 to -3.95 | 0.01 | LLR | 14 | 8 652 |
| Perinatal mortality | -14.13* | -25.19 to -3.06 | 0.01 | Linear | 14 | 8 652 |
| Perinatal mortality | -20.29 | -40.70 to 0.12 | 0.05 | Quadratic | 14 | 8 652 |
| Perinatal mortality | -36.29 | -76.96 to 4.37 | 0.08 | Cubic | 14 | 8 652 |
| Perinatal mortality | -10.69** | -18.32 to -3.05 | 0.01 | LLR | 28 | 18 195 |
| Perinatal mortality | -8.27* | -15.24 to -1.29 | 0.02 | Linear | 28 | 18 195 |
| Perinatal mortality | -15.50** | -26.95 to -4.06 | 0.01 | Quadratic | 28 | 18 195 |
| Perinatal mortality | -19.75* | -37.44 to -2.05 | 0.03 | Cubic | 28 | 18 195 |
| Perinatal mortality | -7.31* | -13.40 to -1.22 | 0.02 | LLR | 42 | 27 698 |
| Perinatal mortality | -5.66* | -11.23 to -0.09 | 0.05 | Linear | 42 | 27 698 |
| Perinatal mortality | -10.46* | -19.21 to -1.72 | 0.02 | Quadratic | 42 | 27 698 |
| Perinatal mortality | -17.74** | -30.48 to -5.01 | 0.01 | Cubic | 42 | 27 698 |
| Perinatal mortality | -5.85* | -11.06 to -0.63 | 0.03 | LLR | 56 | 36 911 |
| Perinatal mortality | -4.55 | -9.30 to 0.19 | 0.06 | Linear | 56 | 36 911 |
| Perinatal mortality | -8.17* | -15.52 to -0.81 | 0.03 | Quadratic | 56 | 36 911 |
| Perinatal mortality | -13.94** | -24.29 to -3.60 | 0.01 | Cubic | 56 | 36 911 |
|  |  |  |  |  |  |  |
| ** p<0.01, * p<0.05 |  |  |  |  |  |  |

ITT: Intention-to-treat

95% CI: 95% Confidence Interval

Bandwidth: number of days between upper and lower limit of the bandwidth

Table C. Effects of the July 20th, 2015 ceasefire on fetal deaths and perinatal mortality. LLR and parametric regressions by order of polynomial for M-p75 municipalities

|  | ITT effect | 95% CI | p value | Functional form | Bandwidth | Observations in bandwidth |
| --- | --- | --- | --- | --- | --- | --- |
|  |  |  |  |  |  |  |
| Stillbirth | -14.45** | -23.67 to -5.23 | 0.00 | LLR | 14 | 11 723 |
| Stillbirth | -12.80** | -20.97 to -4.64 | 0.00 | Linear | 14 | 11 723 |
| Stillbirth | -19.85* | -35.41 to -4.29 | 0.01 | Quadratic | 14 | 11 723 |
| Stillbirth | -32.34* | -62.23 to -2.44 | 0.03 | Cubic | 14 | 11 723 |
| Stillbirth | -7.57** | -13.14 to -2.00 | 0.01 | LLR | 28 | 24 578 |
| Stillbirth | -5.69* | -10.55 to -0.82 | 0.02 | Linear | 28 | 24 578 |
| Stillbirth | -11.64** | -20.10 to -3.17 | 0.01 | Quadratic | 28 | 24 578 |
| Stillbirth | -20.84** | -34.19 to -7.48 | 0.00 | Cubic | 28 | 24 578 |
| Stillbirth | -4.85* | -9.17 to -0.53 | 0.03 | LLR | 42 | 37 292 |
| Stillbirth | -3.00 | -6.89 to 0.90 | 0.13 | Linear | 42 | 37 292 |
| Stillbirth | -8.54** | -14.86 to -2.22 | 0.01 | Quadratic | 42 | 37 292 |
| Stillbirth | -14.20** | -23.79 to -4.61 | 0.00 | Cubic | 42 | 37 292 |
| Stillbirth | -3.38 | -7.02 to 0.26 | 0.07 | LLR | 56 | 49 773 |
| Stillbirth | -2.05 | -5.29 to 1.20 | 0.22 | Linear | 56 | 49 773 |
| Stillbirth | -5.67* | -10.92 to -0.43 | 0.03 | Quadratic | 56 | 49 773 |
| Stillbirth | -11.32** | -18.95 to -3.69 | 0.00 | Cubic | 56 | 49 773 |
|  |  |  |  |  |  |  |
| Perinatal mortality | -13.36** | -23.49 to -3.22 | 0.01 | LLR | 14 | 11 723 |
| Perinatal mortality | -11.49* | -20.68 to -2.30 | 0.01 | Linear | 14 | 11 723 |
| Perinatal mortality | -18.20* | -35.24 to -1.16 | 0.04 | Quadratic | 14 | 11 723 |
| Perinatal mortality | -36.38* | -69.64 to -3.11 | 0.03 | Cubic | 14 | 11 723 |
| Perinatal mortality | -6.86* | -13.24 to -0.48 | 0.04 | LLR | 28 | 24 578 |
| Perinatal mortality | -4.53 | -10.27 to 1.22 | 0.12 | Linear | 28 | 24 578 |
| Perinatal mortality | -11.64* | -21.18 to -2.10 | 0.02 | Quadratic | 28 | 24 578 |
| Perinatal mortality | -19.16* | -33.90 to -4.41 | 0.01 | Cubic | 28 | 24 578 |
| Perinatal mortality | -3.92 | -8.98 to 1.13 | 0.13 | LLR | 42 | 37 292 |
| Perinatal mortality | -2.42 | -7.03 to 2.19 | 0.30 | Linear | 42 | 37 292 |
| Perinatal mortality | -6.91 | -14.20 to 0.37 | 0.06 | Quadratic | 42 | 37 292 |
| Perinatal mortality | -14.11** | -24.78 to -3.44 | 0.01 | Cubic | 42 | 37 292 |
| Perinatal mortality | -2.67 | -6.98 to 1.64 | 0.22 | LLR | 56 | 49 773 |
| Perinatal mortality | -1.48 | -5.39 to 2.42 | 0.46 | Linear | 56 | 49 773 |
| Perinatal mortality | -4.81 | -10.93 to 1.31 | 0.12 | Quadratic | 56 | 49 773 |
| Perinatal mortality | -9.90* | -18.56 to -1.23 | 0.03 | Cubic | 56 | 49 773 |
|  |  |  |  |  |  |  |
| ** p<0.01, * p<0.05 |  |  |  |  |  |  |

ITT: Intention-to-treat

95% CI: 95% Confidence Interval

Bandwidth: number of days between upper and lower limit of the bandwidth

1. **Effect of the July 20th, 2015 ceasefire on prenatal care utilization**

As an exploration of a possible mechanism to explain our main results, we estimated the effect of the July 20th, 2015 ceasefire on prenatal care visits. This was the only healthcare access proxy variable for pregnant women in our data. We standardized the number of prenatal care visits by the duration of pregnancy in months. Table D shows the results of regression discontinuity analysis with local linear regression (LLR) and alternative bandwidths (28, 42 and 56 days). We do not find any statistically significant effects of the July 20th, 2015 ceasefire on prenatal care visits.

Table D. Effects of the July 20th, 2015 ceasefire on prenatal care visits per month

|  | ITT effect | 95% CI | p value | Functional form | Bandwidth | Observations in bandwidth |
| --- | --- | --- | --- | --- | --- | --- |
|  |  |  |  |  |  |  |
| M-p90 municipalities | 0.00 | -0.02 to 0.02 | 0.93 | LLR | 28 | 18 030 |
| M-p90 municipalities | 0.00 | -0.01 to 0.02 | 0.92 | LLR | 42 | 27 434 |
| M-p90 municipalities | 0.00 | -0.01 to 0.02 | 0.48 | LLR | 56 | 36 566 |
|  |  |  |  |  |  |  |
| M-p75 municipalities | -0.00 | -0.02 to 0.01 | 0.64 | LLR | 28 | 24 378 |
| M-p75 municipalities | -0.00 | -0.02 to 0.01 | 0.57 | LLR | 42 | 36 963 |
| M-p75 municipalities | 0.00 | -0.01 to 0.01 | 0.86 | LLR | 56 | 49 333 |
|  |  |  |  |  |  |  |

** p<0.01, * p<0.05

ITT: Intention-to-treat

95% CI: 95% Confidence Interval

Bandwidth: number of days between upper and lower limit of the bandwidth

1. **References**

1 Bor J, Moscoe E, Mutevedzi P, Newell M-L, Bärnighausen T. Regression discontinuity designs in epidemiology: causal inference without randomized trials. *Epidemiology* 2014; **25**: 729–37.

2 Venkataramani AS, Bor J, Jena AB. Regression discontinuity designs in healthcare research. *BMJ* 2016; **352**: i1216.

3 Moscoe E, Bor J, Bärnighausen T. Regression discontinuity designs are underutilized in medicine, epidemiology, and public health: a review of current and best practice. *J Clin Epidemiol* 2015; **68**: 122–33.

4 Hahn J, Todd P, Van der Klaauw W. Identification and Estimation of Treatment Effects with a Regression-Discontinuity Design. *Econometrica* 2001; **69**: 201–9.

5 Imbens G, Kalyanaraman K. Optimal Bandwidth Choice for the Regression Discontinuity Estimator. *Rev Econ Stud* 2012; **79**: 933–59.

6 Lee DS, Lemieux T. Regression Discontinuity Designs in Economics. *Journal of Economic Literature* 2010; **48**: 281–355.

7 McCrary J. Manipulation of the running variable in the regression discontinuity design: A density test. *Journal of Econometrics* 2008; **142**: 698–714.

1. Colombia Implementation Working Group. Colombia: A strategy to improve the registration and certification of vital events in rural and ethnic communities. CRVS country perspectives. Melbourne, Australia: Bloomberg Philanthropies Data for Health Initiative, Civil Registration and Vital Statistics Improvement, the University of Melbourne; 2018 [↑](#footnote-ref-1)
2. Toro Roa, Juan Pablo; Iunes, Roberto F.; Mills, Samuel. 2019. Achieving Health Outcomes in Colombia: Civil Registration and Vital Statistics System, Unique Personal Identification Number, and Unified Beneficiary Registry System for Births and Deaths. Health, Nutrition, and Population Discussion Paper; World Bank, Washington, DC. World Bank. https://openknowledge.worldbank.org/handle/10986/32538 License: CC BY 3.0 IGO. [↑](#footnote-ref-2)
