# Supplementary material for: Conflict violence reduction and pregnancy outcomes: A regression discontinuity design in Colombia
Source: PLoS Med. 2021 Jul 6;18(7):e1003684. doi: 10.1371/journal.pmed.1003684 (PMC8259980; doi:10.1371/journal.pmed.1003684)
Supplement: Attachment — Submitted filename: buitrago.pdf [file pmed.1003684.s004.pdf]

This paper uses various sources of data to examine the relationship between violence and pregnancy outcomes in Colombia.

I was asked for a statistical report and I interpret that to include all aspects of the design and conduct of the study.

Points of detail

Page 3 Strictly speaking the percentile is the value at the category boundary but it is hard to find a suitable alternative word here.

Page 8 If I understand this correctly M–p90 contains regions with many events attributed to FARC irrespective of the activities of the other two organisations shown in Figure A3: ELM and AUC. Perhaps that could be clarified? More importantly we do not seem to have a justification for the choice of those particular percentages.

Page 10 I think the term local linear may need an extra sentence or two of explanation. The notion here may be more familiar to economists than people in health. People may, for instance, be surprised at seeming a linear model which involves estimating coefficients described as non–parametric. A bit more explanation of the linear, quadratic and cubic models used as sensitivity analyses would not be a bad idea either. I assume they are separate global fits to all the data before the discontinuity and to all the after data

Page 12 The miscarriage results are important so I think the numerical results should be shown here.

Page 15 This gives the bandwidth as one month but Figure 2 in the caption states 56 days. I can guess how these could be reconciled but perhaps a clarification might be in order.

Page 24, Table 2 I found this hard to grasp. As I understand it FARC was the dominant force during the armed struggle. I do not follow Colombia that closely in our media but it was the only organisation which was reported on here so I accept it was the major one. If the events here are all conflict related then how does the FARC value form such a small part of the total? If the bulk of these relate to the Colombian army then that could be mentioned.

Figure A7 and A8 Is there any explanation for the consistent fall in maternal age during the before ceasefire period? Is this a possible confounding variable?

Table A2 Although using different bandwidths is a useful supplementary analysis I would question whether fitting a global polynomial model is worth doing. The regression discontinuity design, if I understand it correctly, places most emphasis on the time period closest to the discontinuity. Following the arguments in Magee (1998) a polynomial is not the function which has that characteristic as it is influenced by the observations throughout the range.

Summary

Mostly for clarification. The justification of the choice of 90 and 75 is important.

Michael Dewey

References

L Magee. Nonlocal behavior in polynomial regressions. *American Statistician*, 52:20–22, 1998.
