# Supplementary material for: Conflict violence reduction and pregnancy outcomes: A regression discontinuity design in Colombia
Source: PLoS Med. 2021 Jul 6;18(7):e1003684. doi: 10.1371/journal.pmed.1003684 (PMC8259980; doi:10.1371/journal.pmed.1003684)
Supplement: Attachment — Submitted filename: PMEDICINE-D-21-00816_reviewerannotated.pdf [file pmed.1003684.s005.pdf]

PLOS Medicine

The Effects of Conflict Violence Reduction on Pregnancy Outcomes: Evidence from a Regression Discontinuity Design in Colombia

--Manuscript Draft--

**Manuscript Number:**	PMEDICINE-D-21-00816
**Full Title:**	The Effects of Conflict Violence Reduction on Pregnancy Outcomes: Evidence from a Regression Discontinuity Design in Colombia
**Short Title:**	Conflict Violence Reduction and Pregnancy Outcomes: a Regression Discontinuity Design in Colombia
**Article Type:**	Research Article
**Section/Category:**	Global Health
**Abstract:**	BACKGROUND<br>The impacts of exposure to conflict violence during pregnancy on the risks of miscarriage, stillbirth and perinatal mortality have not been rigorously studied. The signing of the 2016 peace accord in Colombia between the government and FARC guerrillas was preceded by a definitive ceasefire in July 2015 that introduced differential changes in the levels of conflict violence across municipalities. This setting generates a natural experiment that we exploit to credibly assess the causal effects of reduced exposure to conflict violence during pregnancy on the risks of adverse pregnancy outcomes.<br>METHODS AND FINDINGS<br>We adopted a regression discontinuity design using the July 2015 ceasefire declared during the Colombian peace process as an exogenous discontinuous change in exposure to conflict events during pregnancy, comparing women with conception dates before and after the ceasefire date. We constructed the cohorts of all pregnant women in Colombia for each day between January 1, 2013 and December 31, 2017 using birth and death certificates. A total of 3 254 696 women were followed until the end of pregnancy (fetal death or live birth). We measured conflict exposure as the total number of conflict events that occurred in the municipality where a pregnant woman lived during her pregnancy. We first assessed whether the ceasefire did induce a discontinuous fall in conflict exposure for women with conception dates after the ceasefire, to then estimate the effect of this reduced exposure to violence on the risks of miscarriage, stillbirth, and perinatal mortality. We found that the July 2015 ceasefire reduced the average number of conflict events to which women were exposed during pregnancy in their municipalities of residence (absolute effect -0·20; 95% CI -0·33 to -0·08). This effect was greater in municipalities where FARC had a greater presence historically. The reduction in average exposure to conflict violence resulted in an absolute decrease of 9·53 stillbirths per 1 000 pregnancies (95% CI -16·13 to -2·93) for municipalities in the 90th percentile of the distribution of FARC-related conflict events, and a decrease of 7·57 (95% CI -13·14 to -2·00) for municipalities in the 75th percentile of FARC-related events. For perinatal mortality, we estimated reductions of 10·69 (95% CI -18·32 to -3·05) and 6·86 (95% CI -13·24 to -0·48) deaths per 1 000 pregnancies for the two types of municipalities, respectively. We found no effects for miscarriages. Formal tests support the validity of the key regression discontinuity assumptions in our data, while a battery of sensitivity analyses and falsification tests confirm the robustness of our empirical conclusions.<br>CONCLUSIONS<br>Our study offers rigorous evidence that reduced exposure to conflict violence during pregnancy can cause important (previously unmeasured) benefits in terms of reducing the risk of stillbirth and perinatal deaths. The findings are consistent with such beneficial effects manifesting themselves mainly through reduced violence exposure during the early stages of pregnancy. Beyond the relevance of this evidence for other countries beset by chronic armed conflicts, our results support the view that the fledgling Colombian peace process has already contributed to better population health.
**Additional Information:**	
**Question**	**Response**

**Financial Disclosure**<br><br>Enter a financial disclosure statement that describes the sources of funding for the work included in this submission. Review the submission guidelines for detailed requirements. View published research articles from *PLOS Medicine* for specific examples.<br><br>This statement is required for submission and **will appear in the published article** if the submission is accepted. Please make sure it is accurate.<br><br>**Unfunded studies**<br>Enter: *The author(s) received no specific funding for this work.*<br><br>**Funded studies**<br>Enter a statement with the following details:<br>• Initials of the authors who received each award<br>• Grant numbers awarded to each author<br>• The full name of each funder<br>• URL of each funder website<br>• Did the sponsors or funders play any role in the study design, data collection and analysis, decision to publish, or preparation of the manuscript?<br>• **NO** - Include this sentence at the end of your statement: *The funders had no role in study design, data collection and analysis, decision to publish, or preparation of the manuscript.*<br>• **YES** - Specify the role(s) played.<br><br>* typeset	GB was supported by the School of Medicine at Universidad Nacional de Colombia and University of York (CHE Research Fellowship). RMS was supported by a research grant from the UK Medical Research Council (MR/R013667/1). he funders had no role in study design, data collection and analysis, decision to publish, or preparation of the manuscript.
**Competing Interests**<br><br>Use the instructions below to enter a competing interest statement for this submission. On behalf of all authors, disclose any competing interests that could be perceived to bias this work—acknowledging all financial supportand any other relevant financial or non-financial competing interests.	The authors have declared that no competing interests exist.

This statement **will appear in the published article** if the submission is accepted. Please make sure it is accurate. View published research articles from *PLOS Medicine* for specific examples.<br><br>**NO authors have competing interests**<br><br>Enter: *The authors have declared that no competing interests exist.*<br><br>**Authors with competing interests**<br>Enter competing interest details beginning with this statement:<br><br>*I have read the journal's policy and the authors of this manuscript have the following competing interests: [insert competing interests here]*<br><br>* typeset<br><br>This statement is required for submission and **will appear in the published article** if the submission is accepted. Please make sure it is accurate and that any funding sources listed in your Funding Information later in the submission form are also declared in your Financial Disclosure statement.	

Full paper Click here to access/download;Manuscript;Paper_final.docx

Title: The Effects of Conflict Violence Reduction on Pregnancy Outcomes: Evidence from a Regression Discontinuity Design in Colombia

Authors: Giancarlo Buitrago[1,2], Rodrigo Moreno-Serra[3]

1. Clinical Research Institute. School of Medicine. Universidad Nacional de Colombia. 11321 Bogota, Colombia.

2. Hospital Universitario Nacional de Colombia. 11321 Bogota, Colombia.

3. Centre for Health Economics. University of York (UK). Heslington, York, YO10 5DD, UK

Correspondence to:

Dr Giancarlo Buitrago. Clinical Research Institute. School of Medicine. Universidad Nacional de Colombia. Cl. 44 No. 45-99. 11323 Bogotá, Colombia. gbuitragog@unal.edu.co

ABSTRACT

BACKGROUND

The impacts of exposure to conflict violence during pregnancy on the risks of miscarriage, stillbirth and perinatal mortality have not been rigorously studied. The signing of the 2016 peace accord in Colombia between the government and FARC guerrillas was preceded by a definitive ceasefire in July 2015 that introduced differential changes in the levels of conflict violence across municipalities. This setting generates a natural experiment that we exploit to credibly assess the causal effects of reduced exposure to conflict violence during pregnancy on the risks of adverse pregnancy outcomes.

METHODS AND FINDINGS

We adopted a regression discontinuity design using the July 2015 ceasefire declared during the Colombian peace process as an exogenous discontinuous change in exposure to conflict events during pregnancy, comparing women with conception dates before and after the ceasefire date. We constructed the cohorts of all pregnant women in Colombia for each day between January 1, 2013 and December 31, 2017 using birth and death certificates. A total of 3 254 696 women were followed until the end of pregnancy (fetal death or live birth). We measured conflict exposure as the total number of conflict events that occurred in the municipality where a pregnant woman lived during her pregnancy. We first assessed whether the ceasefire did induce a discontinuous fall in conflict exposure for women with conception dates after the ceasefire, to then estimate the effect of this reduced exposure to violence on the risks of miscarriage, stillbirth, and perinatal mortality. We found that the July 2015 ceasefire reduced the average number of conflict events to which women were exposed during pregnancy in their municipalities of residence (absolute effect -0·20; 95% CI -0·33 to -0·08). This effect was greater in municipalities where FARC had a greater presence historically. The reduction in average exposure to conflict violence resulted in an

absolute decrease of 9·53 stillbirths per 1 000 pregnancies (95% CI -16·13 to -2·93) for municipalities in the 90th percentile of the distribution of FARC-related conflict events, and a decrease of 7·57 (95% CI -13·14 to -2·00) for municipalities in the 75th percentile of FARC-related events. For perinatal mortality, we estimated reductions of 10·69 (95% CI -18·32 to -3·05) and 6·86 (95% CI -13·24 to -0·48) deaths per 1 000 pregnancies for the two types of municipalities, respectively. We found no effects for miscarriages. Formal tests support the validity of the key regression discontinuity assumptions in our data, while a battery of sensitivity analyses and falsification tests confirm the robustness of our empirical conclusions.

CONCLUSIONS

Our study offers rigorous evidence that reduced exposure to conflict violence during pregnancy can cause important (previously unmeasured) benefits in terms of reducing the risk of stillbirth and perinatal deaths. The findings are consistent with such beneficial effects manifesting themselves mainly through reduced violence exposure during the early stages of pregnancy. Beyond the relevance of this evidence for other countries beset by chronic armed conflicts, our results support the view that the fledgling Colombian peace process has already contributed to better population health.

INTRODUCTION

Over 135 million people today need humanitarian assistance, the vast majority due to armed conflicts.[1] These conflicts have disproportionately affected low- and middle-income countries (LMICs) since the mid-20th century, with devastating consequences for health and development.[2,3] There has been much interest in recent years in understanding how conflicts affect different health outcomes. Pregnant women and children have received special attention for the study of these effects, due to their high vulnerability to conflict violence[2,4,5], which in LMICs is often compounded by pervasive healthcare inequities that contribute to higher rates of maternal and infant mortality than in richer countries.[6,7] Indeed, several studies have found that exposure to conflict during pregnancy is associated with short- and long-term adverse outcomes for mothers and their children, both in terms of health and human capital, particularly for poor individuals.[8–13]

Obtaining causal evidence about the relationship between exposure to conflict violence and pregnancy outcomes poses great methodological challenges, since it is impossible to randomize conflict exposure, there are many possible confounders, and good quality information from contexts of armed conflict is scarce. This includes often patchy information about pregnancy histories in most countries, leading research to focus on pregnancy outcomes related to live births (hence relying on selected samples). A recent systematic review could find only a few analyses of the associations between conflict exposure and miscarriages (two studies), stillbirth risk (five studies) and perinatal mortality (two studies).[14–20] All these studies are observational andsuffer from data and/or methodological limitations that make causal inference unwarranted.[13]

Our study provides causal evidence of the impacts of conflict exposure on pregnancy outcomes, taking advantage of a natural experiment created by specific features of the conflict and peace

process in Colombia. Colombia has endured one of the longest civil conflicts in history, with over 200 000 direct fatalities and millions of non-fatal victims (mainly civilians) during more than five decades.[21] In 2012, the government began a period of peace talks with the country's largest rebel guerrilla, the FARC (*Fuerzas Armadas Revolucionarias de Colombia*). These conversations, known as the Havana Talks, concluded in November 2016 with the signing of a definitive peace agreementbetween FARC and the government. During the Havana talks, FARC declared unilateral ceasefires on five occasions before a final, bilateral ceasefire was agreed. Because these ceasefires mandateda cessation of violent activities by FARC specifically, but not by other rebel groups, the actual reduction in violence should have occurred primarily – if not only – in the geographic areas where FARC used to be active. For our empirical purposes, a ceasefire introduces a plausibly exogenous change in exposure to violence for pregnant women. We are then able to exploit this exogenous change, combined with rich information about pregnancy histories, to determine how the risks of miscarriage, stillbirth and perinatal death are causally affected by a reduction in the intensity of conflict violence to which a pregnant woman is exposed in her municipality of residence.

METHODS

Pregnancy cohorts and outcomes

This study included all pregnant women in Colombia between Jan/01/2013 and Dec/31/2017. We constructed daily single pregnancy cohorts for each of the 1 826 days in the study period. We defined cohorts by the probable conception date. All women were followed until pregnancy ended in either a live birth or fetal death. Newborns were followed since birth until their 7th day of life. We constructed each pregnancy cohort based on birth and death certificates from the Ministry of Health's Single Registry of Enrollees. All live births were identified from birth certificates, and all fetal deaths and deaths before the 7th day of life were identified from death certificates. We

merged these two databases using an anonymized identifier. The conception date was estimated based on the gestational age recorded in both certificates. These data sources were provided by the Colombian Ministry of Health to the Clinical Research Institute of *Universidad Nacional de Colombia* for use in our research (see Supplementary material, Section 1. Information sources and databases, for a full description of each information source and the construction of the final database).

Since competing risks of loss in early pregnancy may affect pregnancy outcomes, we followed recent recommendations and used perinatal mortality as our main outcome, defined as death at any point between week 22 of pregnancy (day 154) and the 7th day after birth.[22] We were able to examine fetal deaths also in terms of stillbirth (fetal death after 22 weeks of pregnancy) and miscarriage (fetal death before week 22 of pregnancy).[23]

Exposure to conflict violence

We measured individual exposure to conflict violence as the total number of conflict events that occurred in a woman's municipality of residence during her pregnancy. Conflict data was obtained from the Memory and Conflict Observatory database (National Center for Historical Memory, CNMH). This database combines information on conflict-related events from several independent sources, being a leading data source for research about the Colombian conflict due to its reliability and completeness.[24] We downloaded the publicly available CNMH dataset from http://centrodememoriahistorica.gov.co in December 2018, including information about all types of conflict-related events (e.g. murders, kidnappings, sexual violence, landmines and others) by location. We identified all conflict events that occurred on each day, in each Colombian

municipality, between 2013 and 2018 (we included 2018 because women whose pregnancy began at the end of 2017 were also potentially exposed to events in 2018).[1]

We determined exposure to conflict violence for each pregnant woman included in our final dataset, based on the occurrence of conflict events in the municipality where the woman resided during her pregnancy. With the daily information about pregnancy cohorts, we could identify the total number of conflict-related events to which each woman was exposed from the beginning to end of each pregnancy. A woman's municipality of residence was presumed to be the one reported on the birth certificate for live births, or death certificate for fetal deaths. Wherever possible, we also cross-verified the municipality of residence using official information from health insurance enrolment and healthcare provision databases, to identify whether, during pregnancy, the woman made contact with the health system in the municipality that was assigned as that of residence.

Ceasefires during the Havana talks

FARC were involved in seven ceasefires during the Havana talks. Only the last two ceasefires before the peace accord, on Jul/20/2015 and Aug/29/2016, were declared for an indefinite period and lasted more than just a few months, holding until the date of the final peace accord itself (Supplementary material, Figure A2). The Jul/20/2015 ceasefire was declared unilaterally by FARC, followed in Aug/29/2016 by a definitive ceasefire by the Colombian government, close to the signing of the peace accord. The other five ceasefires were limited in duration (lasting no more than four months) and violence returned to the municipalities soon after these ended.

[1] We excluded from the analysis the events for which it was impossible to determine the date or place of occurrence, representing only 1.11% of all events reported by CNMH.

Therefore, we focused on the Jul/20/2015 ceasefire for our empirical analyses. This is the first ceasefire that can be expected to have introduced a sudden and permanent reduction in the levels of FARC violence across municipalities, since it was the definitive ceasefire declared and adhered to by FARC. Nonetheless, we also assessed, using regression discontinuity analysis (see Results), whether the Aug/29/2016 ceasefire that involved both government and FARC, which in principle could have generated another sharp and lasting reduction in violence, did in fact lead to such reduction.

FARC presence across municipalities

Several groups other than FARC were responsible for armed violence in certain areas during the study period (Supplementary material, Figure A3). The ceasefires involving FARC could be expected to have affected violence levels mainly in the areas where FARC historically had more presence. To identify those areas, we grouped all municipalities into categories of FARC-related conflict intensity, based on the distribution of the total number of conflict events involving FARC for the years 2000 through to 2017, thus identifying those municipalities with persistent FARC presence over the past 17 years. We created four categories of municipalities: 1) M-p90: municipalities with total number of FARC-related violent events above the 90th percentile of the distribution of these events; 2) M-p75: municipalities with total number of FARC-related events above the 75th percentile; 3) M-zero: municipalities with no conflict event recorded during the 2000-2017 period (whether involving FARC or not); and 4) M-other: municipalities that did not belong to the other three groups (Figure 1).

Estimation methodology

We adopted a quasi-experimental regression discontinuity (RD) approach, a methodological design that is well suited to permit causal inference in study settings like ours.[25,26] The ceasefires involving FARC can be regarded as introducing exogenous changes – discontinuities – in the number of conflict events in the municipalities where women lived during their pregnancies. This setting creates a natural experiment allowing the comparison of outcomes between women with conception dates before and after a ceasefire date (the threshold). The sudden, discontinuous change in exposure to conflict violence implies that assignment to different levels of conflict violence is as good as random for women close to either side of the threshold.[25,26] If the underlying assumptions of the RD design are met (which we can test with the data available), we can use the discontinuous change induced by a ceasefire to estimate the causal effect of exposure to violence on pregnancy outcomes.[27–29] We used the conception date for each cohort as the running variable, and the date of the Jul/20/2015 ceasefire as the discontinuity threshold (Supplementary material, Section 3).

We implemented our RD approach through a sequence of estimations steps, each of these subject to formal tests to assess result robustness. First, we assessed whether the Jul/20/2015 ceasefire actually produced a discontinuity in the total number of conflict events to which women were exposed during pregnancy, separately for events involving a) FARC and b) any armed group (not necessarily FARC). We did so for all municipalities and for each of the categories that we created based on FARC-related events, anticipating that municipalities with greater FARC presence (M-p90 and M-p75) would have seen larger changes in violence induced by the Jul/20/2015 ceasefire, than

municipalities where FARC had less/no presence or where there was no conflict violence prior to the ceasefire (M-Zero and M-other). We repeated this process for the Aug/29/2016 ceasefire. Second, for the categories of municipalities where we identified a discontinuity in violence, measured by either FARC-related events or total conflict events, we estimated the RD effects of the ceasefire on the risks of perinatal mortality, stillbirth, and miscarriage. We performed these estimations using a non-parametric approach (local linear regression) with optimal bandwidth.[30,31] Similarly to a randomized experiment with imperfect adherence, exposure to conflict took a nondeterministic form (women exposed and not exposed to conflict were on both sides of the threshold).[25] The parameter of interest was the intention-to-treat estimate.

Third, we tested the validity of the basic RD assumptions. The RD estimates could be biased if the key assumptions of no-manipulation and continuity of baseline variables do not hold in our data. We evaluated the no-manipulation assumption graphically and statistically (McCrary tests).[27] We also tested statistically for the presence of any discontinuities in several observable baseline characteristics between women with conception dates before and after the ceasefire. Lastly, we conducted a battery of sensitivity analyses and falsification tests for our main results. We re-ran all our RD estimations using alternative bandwidths and a parametric approach with first-, second-, and third-order polynomial specifications. We also estimated placebo effects by re-estimating our models using solely the samples of women living in the municipalities where no discontinuities in conflict events were found (M-zero and M-other), and hence where no effects on pregnancy outcomes should be expected.

For all estimations we report 95% confidence intervals. The analyses were performed using Stata 15®.

RESULTS

A total of 3 661 022 birth certificates and 1 441 140 death certificates were issued between Jan/2013 and Dec/2017 in Colombia (Supplementary material, Figure A1). After identifying single pregnancies with conception dates between Jan/01/2013 and Dec/31/2017, the total cohort included 3 254 696 pregnant women. Table 1 presents the characteristics of the cohort for the full sample and separately for women in M-p90 and M-p75 municipalities. Women in M-p90 and M-p75 municipalities had higher pregnancy-related mortality rates than the national average.

Exposure to conflict violence

On average, the women in our study were exposed to 3.76 (95% CI 3.75 to 3.77) conflict events of any type during pregnancy, with higher exposure to events of selective murder (1.53 events) and sexual violence (0.98 events) (Table 2). Women in M-p90 municipalities were exposed to a higher number of total conflict events on average during pregnancy (7.64; 95% CI 7.62 to 7.66) than women in M-p75 municipalities (6.31; 95% CI 6.29 to 6.32).

Exposure to conflict events during pregnancy showed a sustained falling trend throughout the study period, despite a few spikes (Supplementary material, Figure A4). Instead of relying on visual inspection, we use RD estimation to identify the presence of discontinuities in violence intensity around the ceasefires declared during the study period. We found that the Jul/20/2015 ceasefire significantly affected exposure to conflict events, both FARC-related and total (i.e. not necessarily FARC-related) (Figure 2 and A5, Supplementary material). It resulted in a decrease in exposure to violence during pregnancy of 0.20 total events and 0.02 FARC-related events on average, for women with conception dates after the ceasefire (Figure 2). We also found that this ceasefire only reduced conflict exposure for women in M-p90 and M-p75 municipalities. By contrast, repeating

these analyses for the Aug/29/2016 ceasefire reveals no statistically significant changes in exposure to conflict events during pregnancy (Figure 2).

Effects of reduced conflict exposure on miscarriages, stillbirths, and perinatal mortality

Figure 3 shows that the decreased exposure to conflict events during pregnancy, induced by the Jul/20/2015 ceasefire, reduced stillbirths by 9.53 (95% CI -16.13 to -2.93) and perinatal deaths by 10.69 (95% CI -18.32 to -3.05) per 1 000 pregnancies for women in M-p90 municipalities. These figures represent relative reductions of 62.32% in stillbirths and 53.69% in perinatal mortality (Supplementary material, Table A1). For pregnant women in M-p75 municipalities, we estimated reductions of 7.57 (95% CI -13.14 to -2.00) in stillbirths and 6.86 (95% CI -13.24 to -0.48) in perinatal deaths per 1 000 pregnancies, corresponding to relative reductions of 55.09% in stillbirths and 40.19% in perinatal mortality. We did not find statistically significant effects for miscarriages.

Validity of the RD assumptions

Formal tests support the validity of the underlying RD assumptions in our data, and hence of causal inference based on RD estimation (Supplementary material, Figure A6). Both the histograms of the distribution of women according to conception dates (Panel A) and the McCrary tests (Panel B) showed no evidence of manipulation, i.e. there are no discontinuities in the density function of conception dates around the ceasefire (threshold). We also conducted graphical and statistical analyses to assess balance in baseline characteristics between pregnant women on each side of the threshold, and again found no evidence of discontinuity for any of these variables (Supplementary material, Figures A7 and A8). Taken together, the results above offer statistical

evidence that exposure to different levels of conflict violence during pregnancy is as good as random for women on both sides of the Jul/20/2015 ceasefire.

Robustness checks

We tested the sensitivity of our main results to using several different combinations of functional forms and bandwidths. We found that our results of statistically significant reductions in both stillbirths and perinatal mortality due to the reduction in violence after the Jul/20/2015 ceasefire are robust to all these specification changes (Supplementary material, Tables A2-A3).

We also estimated the effects of the Jul/20/2015 ceasefire for pregnant women in M-zero and M-other municipalities as placebo tests (as we did not find any discontinuities in exposure to conflict events in those municipalities). We found no evidence of effects on either stillbirths or perinatal mortality (Table 3). All these results provide further reassurance about the validity of our causal estimates.

DISCUSSION

The effects of conflict violence on the health outcomes of non-combatant populations have been widely studied.[32,33] Nevertheless, the causal effects of conflict on perinatal mortality and the risk of stillbirth have not been evaluated previously by studies with low risk of bias.[13] Our study employed a RD approach, using ceasefires dates as thresholds that could introduce exogenous discontinuities in conflict intensity in Colombia, to estimate the causal effect of reduced exposure to conflict events during pregnancy on the risks of miscarriage, stillbirth and perinatal mortality. We found that the Jul/20/2015 ceasefire decreased the total number of conflict events to which

women were exposed during pregnancy, which in turn led to reduced risks of stillbirth and perinatal mortality.

To our knowledge, our study is the first to show a causal relationship between exposure to conflict during pregnancy and the risks of stillbirth and perinatal mortality. In addition to methodological shortcomings, data limitations (particularly around pregnancy records) have prevented previous studies from assessing the aforementioned relationships. For example, Wagner *et al*. found that exposure to conflict increased mortality for 1 year-old, 5 year-olds and for reproductive-aged women, and the risk of children becoming orphans.[5,32] Further studies have found associations between exposure to conflict during pregnancy and other indicators including birth weight, height-for-age and cognitive outcomes.[8,10,34–36] By contrast, for our analysis we were able to reconstruct all pregnancy cohorts in Colombia, since the beginning of each pregnancy, using rich administrative data. This enabled us to measure conflict exposure during pregnancy for all women whose pregnancies began between Jan/01/2013 and Dec/31/2017, following them until the pregnancy ended in fetal death or a live birth. We therefore mitigated the possibility of survival bias influencing our results.

Strengths and limitations of this study

Our findings are consistent with features of the Colombian peace process, offering support to the validity of our RD approach. First, while the number of conflict events decreased during the entire period of the Havana talks, we found that only the Jul/20/2015 ceasefire induced a discontinuity in conflict exposure. This is in line with the fact that for the previous FARC-declared ceasefires the start/end dates were known and close, and/or the Colombian army continued attacking FARC guerrillas, leading to minimal and unsustained changes in violence levels and subsequent rises in

these levels (see spikes in violence between 50 and 100 days before the Jul/20/2015 ceasefire, Figure A5). However, the Havana talks intensified markedly in June-July 2015, leading to FARC's definitive ceasefire and an accompanying reduction in attacks by the Colombian army against FARC, to support the possibility of a definitive peace agreement. This sharp reduction in violent activities involving the key conflict actors after the July 2015 ceasefire was sustained, resulting in violence levels between FARC and the government that were already very low at the time of the Aug/29/2016 bilateral ceasefire.

Second, we found that the Jul/20/2015 ceasefire reduced exposure to violence more in municipalities where historically the FARC had greater presence (M-p90) than in municipalities with less FARC presence (M-p75), while no effect on violence levels was found in municipalities where FARC were not present or where there was no armed conflict. These (expected) patterns enabled us to identify a dose-response relationship between conflict exposure and pregnancy outcomes: the Jul/20/2015 ceasefire caused greater reductions in stillbirths and perinatal mortality in M-p90 than in M-p75 municipalities, with no changes in outcomes in municipalities with no FARC presence or where there were no conflict-related events.

The numerous sensitivity tests undertaken indicate that our estimates are robust, also providing insights for the interpretation of findings. The main results are based on an optimal RD bandwidth of one month.[31] We tested various alternative bandwidth sizes of up to two months, with no material changes in our conclusions, suggesting that exposure to violence very early in the pregnancy is a key driver of the risks of stillbirth and perinatal mortality.

Whilst with the data available we were unable to undertake an in-depth investigation of possible mechanisms linking reductions in conflict exposure to reduced risks of stillbirth and perinatal mortality, evidence from other studies suggest that maternal stress could be a crucial channel.[20,37–40] Simple descriptive analysis of our data offers some support to this possibility. The data on the types of conflict violence to which pregnant women were most vulnerable indicates that the highest exposure rates were related to very stressful and traumatic events (including events showing a direct relationship with sexual and reproductive health), particularly sexual violence and murder. In addition to reduced risk of suffering traumatic episodes themselves, pregnant women may have also benefitted indirectly from the decline in conflict violence following the July 2015 ceasefire, through decreased rates of violent events occurring in the communities where these women lived (likely contributing further to reduced maternal stress), as such "neighborhood effects" have been found to be associated with pregnancy outcomes.[41,42] Changes in healthcare access due to reduced conflict violence may have played a role for improved pregnancy outcomes as well. However, due to data constraints, we were unable to determine the plausibility of a competing "care access" channel vis-à-vis maternal stress. Although in supplementary analyses we did not find evidence that the reduction in conflict exposure following the July 2015 ceasefire led to any changes in prenatal care utilization rates for pregnant women in M-p90 and M-p75 municipalities (Supplementary material, Table A4), we do not have suitable data to investigate any other indicators of access to care during pregnancy which could prove more relevant in the early stages of pregnancy than simple prenatal care utilization (e.g. prenatal care quality, access to essential drugs or diagnostic tests). Finally, the absence of conflict reduction effects on miscarriages may be explained by the fact that such fetal losses occur very early in the pregnancy, and are mainly caused by chromosomal abnormalities or other factors unrelated to violence exposure during pregnancy.[43,44]

We must note some limitations of this study. First, Colombia's administrative records have improved substantially in the last decade, but some data quality issues may remain. One example relevant to our analyses is that the number of miscarriages reported in our data may well be an underestimation of the true number of miscarriages during the study period, since miscarriages may occur without formal healthcare intervention. Unfortunately, there is no information available on the patterns of underreporting of miscarriages across Colombian municipalities, thus precluding an assessment a priori of whether this phenomenon is likely to lead to underestimation of our RD effects of reduced conflict exposure on the risk of miscarriage. Yet it does not seem plausible to expect that the July 2015 ceasefire induced sudden changes (e.g. within one month) in the patterns of underreporting of miscarriages in M-p90 or M-p75 municipalities, which would be the type of change necessary to influence our estimates (note that a systematic but unchanged pattern of underreporting of miscarriages before and after the ceasefire in a given municipality would not affect the validity of our RD estimates). Data constraints limit our ability to scrutinize this possibility further, however. Second, we measured exposure to conflict based on information about the woman's place of residence taken primarily from birth and death certificates. Although we also conducted cross-checks using other administrative databases wherever feasible, we cannot rule out the possibility that some women migrated, at some point of their pregnancy periods, between municipalities with different intensities of conflict violence. Unfortunately, we do not have detailed geographic information that would allow us to construct finer measures of heterogeneity of conflict exposure for women during their pregnancies. Third, in-depth examination of access and quality of care indicators could have provided valuable insights about pathways leading from reduced conflict violence exposure to improved pregnancy outcomes, yet as previously noted we do not have data on indicators beyond prenatal care use that could be

linked up with our dataset of individual pregnancies (such as quality of prenatal care and access to quality care during labor and delivery, or postnatal follow up for mother and child), and therefore we must leave such analyses for future research.

Conclusion

Our study offers rigorous evidence that reduced exposure to conflict violence during pregnancy can cause important (yet previously unmeasured) benefits in terms of reducing the risk of stillbirth and perinatal mortality. Beyond the relevance of this evidence for other countries beset by chronic armed conflicts, our results support the view that the fledgling Colombian peace process has already contributed to better population health.

Acknowledgments

We thank the Office of Information and Communication Technology of the Colombian Ministry of Health and Social Protection (Dolly Ovalle and Luz Emilse Rincon) for providing the anonymized data for this study. Finally, we would like to thank Alexander Buritica for his excellent research assistance in the early phases of this project.

Contributors

GB and RMS conceived and designed the study. GB took the lead in collecting the data and analysis. GB and RMS contributed to the interpretation and writing of the manuscript. GB and RMS had access to all the data and were responsible for the decision to submit the manuscript. All authors critically revised the manuscript and approved the final version.

Funding

GB was supported by the School of Medicine at Universidad Nacional de Colombia and University of York (CHE Research Fellowship). RMS was supported by a research grant from the UK Medical Research Council (MR/R013667/1).

Ethical approval

Our study was granted IRB ethical approval by the Research and Institutional Ethics Committee of the School of Medicine at the Pontificia Universidad Javeriana (Minutes No. 10/2018).

Data sharing

The data of death and birth were provided by the Ministry of Health and Social Protection of Colombia to the National University of Colombia. To access these data, requests should be submitted to the Ministry of Health and Social Protection of Colombia. Conflict data was obtained from the Memory and Conflict Observatory database (National Center for Historical Memory, CNMH), we downloaded the publicly available CNMH dataset from http://centrodememoriahistorica.gov.co in December 2018.

REFERENCES

1 Global Humanitarian Overview 2018 [EN/AR/ES/FR/ZH] - World. ReliefWeb. https://reliefweb.int/report/world/global-humanitarian-overview-2018-enaresfrzh (accessed July 25, 2020).

2 Blattman C, Miguel E. Civil War. *Journal of Economic Literature* 2010; **48**: 3–57.

3 ICRC. Protracted conflict and humanitarian action: some recent ICRC experiences. Geneva: International Committee of the Red Cross, 2016.

4 Akresh R, de Walque D. Armed conflict and schooling : evidence from the 1994 Rwandan genocide. The World Bank, 2008.

5 Wagner Z, Heft-Neal S, Wise PH, *et al.* Women and children living in areas of armed conflict in Africa: a geospatial analysis of mortality and orphanhood. *Lancet Glob Health* 2019; **7**: e1622–31.

6 PAHO. PAHO/WHO | EquiLAC II Project. Pan American Health Organization / World Health Organization. https://www.paho.org/hq/index.php?option=com_content&view=article&id=2674:2010-equilac-ii-project&Itemid=2080&lang=en (accessed Sept 28, 2020).

7 Etienne CF. Equity in health systems. *Rev Panam Salud Publica* 2013; **33**: 79–82.

8 Duque V. Early-life conditions and child development: Evidence from a violent conflict. *SSM Popul Health* 2017; **3**: 121–31.

9 Camacho A. Stress and Birth Weight: Evidence from Terrorist Attacks. *Am Econ Rev* 2008; **98**: 511–5.

10 Mansour H, Rees DI. Armed conflict and birth weight: Evidence from the al-Aqsa Intifada. *Journal of Development Economics* 2012; **99**: 190–9.

11 Chamarbagwala R, Morán HE. The human capital consequences of civil war: Evidence from Guatemala. *Journal of Development Economics* 2011; **94**: 41–61.

12 León G. Civil Conflict and Human Capital Accumulation The Long-term Effects of Political Violence in Perú. *J Human Resources* 2012; **47**: 991–1022.

13 Keasley J, Blickwedel J, Quenby S. Adverse effects of exposure to armed conflict on pregnancy: a systematic review. *BMJ Glob Health* 2017; **2**: e000377.

14 Skokić F, Muratović S, Radoja G. Perinatal and maternal outcomes in Tuzla Canton during 1992-1995 war in Bosnia and Herzegovina. *Croat Med J* 2006; **47**: 714–21.

15 Arnetz B, Drutchas A, Sokol R, Kruger M, Jamil H. 1991 Gulf War exposures and adverse birth outcomes. *US Army Med Dep J* 2013; : 58–65.

16 Makhseed M, el-Tomi N, Moussa MA, Musini VM. Post-war changes in the outcome of pregnancy in Maternity Hospital, Kuwait. *Med Confl Surviv* 1996; **12**: 154–67.

17 Pavlinac I, Marin A, Valić M, *et al.* Influence of the wars in Croatia and Bosnia and Herzegovina on the incidence and outcome of singleton premature births in the Split University Hospital. *cent.eur.j.med* 2008; **3**: 187–93.

18 Valente C. Civil conflict, gender-specific fetal loss, and selection: a new test of the Trivers-Willard hypothesis. *J Health Econ* 2015; **39**: 31–50.

19 Skokić F, Bačaj D, Selimović A, Hasanović E, Muratović S, Halilbašić A. Association of Low Birth Weight Infants and Maternal Sociodemographic Status in Tuzla Canton during 1992-1995 War Period in Bosnia and Herzegovina. *Int J Pediatr* 2010; **2010**: 789183.

20 Wainstock T, Lerner-Geva L, Glasser S, Shoham-Vardi I, Anteby EY. Prenatal stress and risk of spontaneous abortion. *Psychosom Med* 2013; **75**: 228–35.

21 Comisión Nacional de Reparación y Reconciliación (Colombia), editor. ¡Basta ya! Colombia, memorias de guerra y dignidad: informe general, Segunda edición corregida. Bogotá: Centro Nacional de Memoria Histórica, 2013.

22 Kramer MS, Zhang X, Platt RW. Analyzing risks of adverse pregnancy outcomes. *Am J Epidemiol* 2014; **179**: 361–7.

23 WHO | Maternal and perinatal health. WHO. https://www.who.int/maternal_child_adolescent/topics/maternal/maternal_perinatal/en/ (accessed June 11, 2020).

24 Botero JD, Guo W, Mosquera G, *et al.* Gang confrontation: The case of Medellin (Colombia). *PLoS ONE* 2019; **14**: e0225689.

25 Bor J, Moscoe E, Mutevedzi P, Newell M-L, Bärnighausen T. Regression discontinuity designs in epidemiology: causal inference without randomized trials. *Epidemiology* 2014; **25**: 729–37.

26 Oldenburg CE, Moscoe E, Bärnighausen T. Regression Discontinuity for Causal Effect Estimation in Epidemiology. *Curr Epidemiol Rep* 2016; **3**: 233–41.

27 Lee DS, Lemieux T. Regression Discontinuity Designs in Economics. *Journal of Economic Literature* 2010; **48**: 281–355.

28 Maas IL, Nolte S, Walter OB, *et al.* The regression discontinuity design showed to be a valid alternative to a randomized controlled trial for estimating treatment effects. *J Clin Epidemiol* 2017; **82**: 94–102.

29 Venkataramani AS, Bor J, Jena AB. Regression discontinuity designs in healthcare research. *BMJ* 2016; **352**: i1216.

30 Calonico S, Cattaneo MD, Titiunik R. Robust Nonparametric Confidence Intervals for Regression-Discontinuity Designs. *Econometrica* 2014; **82**: 2295–326.

31 Imbens G, Kalyanaraman K. Optimal Bandwidth Choice for the Regression Discontinuity Estimator. *Rev Econ Stud* 2012; **79**: 933–59.

32 Wagner Z, Heft-Neal S, Bhutta ZA, Black RE, Burke M, Bendavid E. Armed conflict and child mortality in Africa: a geospatial analysis. *Lancet* 2018; **392**: 857–65.

33 Wise PH. The Epidemiologic Challenge to the Conduct of Just War: Confronting Indirect Civilian Casualties of War. *Daedalus* 2017; **146**: 139–54.

34 Sharkey PT, Tirado-Strayer N, Papachristos AV, Raver CC. The effect of local violence on children's attention and impulse control. *Am J Public Health* 2012; **102**: 2287–93.

35 Akresh R, Lucchetti L, Thirumurthy H. Wars and child health: Evidence from the Eritrean–Ethiopian conflict. *Journal of Development Economics* 2012; **99**: 330–40.

36 Minoiu C, Shemyakina O. Child Health and Conflict in Côte d'Ivoire. *Am Econ Rev* 2012; **102**: 294–9.

37 Ae-Ngibise KA, Wylie BJ, Boamah-Kaali E, *et al.* Prenatal maternal stress and birth outcomes in rural Ghana: sex-specific associations. *BMC Pregnancy Childbirth* 2019; **19**: 391.

38 Hobel CJ, Dunkel-Schetter C, Roesch SC, Castro LC, Arora CP. Maternal plasma corticotropin-releasing hormone associated with stress at 20 weeks' gestation in pregnancies ending in preterm delivery. *Am J Obstet Gynecol* 1999; **180**: S257-263.

39 Hobel CJ, Goldstein A, Barrett ES. Psychosocial stress and pregnancy outcome. *Clin Obstet Gynecol* 2008; **51**: 333–48.

40 Copper RL, Goldenberg RL, Das A, *et al.* The preterm prediction study: maternal stress is associated with spontaneous preterm birth at less than thirty-five weeks' gestation. National Institute of Child Health and Human Development Maternal-Fetal Medicine Units Network. *Am J Obstet Gynecol* 1996; **175**: 1286–92.

41 Ncube CN, Enquobahrie DA, Albert SM, Herrick AL, Burke JG. Association of neighborhood context with offspring risk of preterm birth and low birthweight: A systematic review and meta-analysis of population-based studies. *Soc Sci Med* 2016; **153**: 156–64.

42 Masi CM, Hawkley LC, Piotrowski ZH, Pickett KE. Neighborhood economic disadvantage, violent crime, group density, and pregnancy outcomes in a diverse, urban population. *Soc Sci Med* 2007; **65**: 2440–57.

43 Colley E, Hamilton S, Smith P, Morgan NV, Coomarasamy A, Allen S. Potential genetic causes of miscarriage in euploid pregnancies: a systematic review. *Hum Reprod Update* 2019; **25**: 452–72.

44 Larsen EC, Christiansen OB, Kolte AM, Macklon N. New insights into mechanisms behind miscarriage. *BMC Med* 2013; **11**: 154.

Table 1. Baseline characteristics of all pregnant women in Colombia between January 1, 2013 and December 31, 2017

	Full sample (n=3 254 696)	M-p90 (n=1 288 771)	M-p75 (n=1 732 875)
**Sociodemographic characteristics**			
Age - mean (SD)	24.75 (6.54)	25.17 (6.57)	24.93 (6.59)
Age - category n (%)			
<18 y	413 830 (13.30)	141 721 (11.82)	210 333 (12.90)
18-34	2 418 248 (77.71)	938 329 (78.28)	1 264 884 (77.59)
35-39	228 047 (7.33)	97 253 (8.11)	126 279 (7.75)
>39 y	51 914 (1.67)	21 435 (1.79)	28 785 (1.77)
Health insurance n (%)			
Contributory	1 323 121 (42.52)	644 642 (53.78)	762 135 (46.75)
Subsidized	1 641 702 (52.75)	485 104 (40.47)	782 635 (48.01)
Other	147 185 (4.73)	68 976 (5.75)	85 491 (5.24)
Education n (%)			
Primary or less	446 123 (14.86)	132 959 (11.39)	221 245 (14.06)
Secondary	701 167 (23.36)	235 451 (20.18)	348 051 (22.11)
Higher than secondary	1 854 835 (61.78)	798 473 (68.43)	1 004 791 (63.83)
Married n (%)	494 081 (15.88)	207 068 (17.27)	259 108 (15.89)
Number of children - mean (SD)	1.87 (1.20)	1.79 (1.09)	1.85 (1.18)
Prenatal care - mean (SD)	6.47 (2.53)	6.63 (2.63)	6.45 (2.61)
**Pregnancy Outcomes**			
Rates per 1 000 pregnancies (95% CI)			
Miscarriage	45.65 (45.43 to 45.88)	72.45 (72.00 to 72.89)	61.48 (61.12 to 61.84)
Stillbirth	8.00 (7.90 to 8.10)	10.13 (9.95 to 10.31)	9.45 (9.30 to 9.59)
Perinatal death	11.86 (11.74 to 11.98)	13.70 (13.49 to 13.91)	13.15 (12.97 to 13.32)

Data are means (Standard Deviation - SD). M-p90: pregnant women in municipalities with total number of FARC-related violent events above the 90th percentile of the distribution of these events. M-p75: pregnant women in municipalities with total number of FARC-related violent events above the 75th percentile of the distribution of these events. 95% CI: 95% Confidence Interval.

Table 2. Exposure to conflict violence events during pregnancy

	Full sample (n=3 254 696)	M-p90 (n=1 288 771)	M-p75 (n=1 732 875)
**Total events**	3.764 (8.555)	7.644 (11.930)	6.308 (10.987)
**Total FARC-related events**	0.417 (2.730)	0.922 (4.219)	0.765 (3.702)
**Type of conflict event**			
Terrorist attack	0.030 (0.171)	0.075 (0.264)	0.056 (0.231)
Act of war	0.414 (1.955)	0.877 (2.942)	0.740 (2.624)
Attack on populations	0.000 (0.013)	0.000 (0.020)	0.000 (0.018)
Selective murder	1.535 (3.385)	3.096 (4.742)	2.408 (4.279)
Kidnapping	0.061 (0.369)	0.123 (0.539)	0.102 (0.487)
Child recruitment	0.108 (0.433)	0.249 (0.636)	0.199 (0.574)
Massacre	0.004 (0.065)	0.004 (0.059)	0.007 (0.082)
Forced disappearance	0.226 (1.497)	0.438 (2.316)	0.353 (2.011)
Damage to property	0.293 (1.292)	0.644 (1.946)	0.523 (1.728)
Sexual violence	0.976 (3.807)	1.891 (4.891)	1.712 (5.070)
Landmine	0.116 (0.973)	0.247 (1.499)	0.208 (1.319)

Data are means (Standard Deviation - SD). M-p90: pregnant women in municipalities with total number of FARC-related violent events above the 90th percentile of the distribution of these events. M-p75: pregnant women in municipalities with total number of FARC-related violent events above the 75th percentile of the distribution of these events.

Table 3. Effects of the July 20th, 2015 ceasefire on fetal deaths and perinatal mortality. Placebo tests using M-other and M-zero municipalities

	ITT effect	95% CI	p value	Functional form	Bandwidth	Observations in bandwidth
M-other municipalities						
Stillbirth	0.91	-3.88 to 5.70	0.71	LLR	28	20 833
Stillbirth	0.69	-3.23 to 4.61	0.73	LLR	42	31 701
Stillbirth	0.98	-2.43 to 4.38	0.57	LLR	56	42 706
Perinatal mortality	5.16	-0.89 to 11.21	0.09	LLR	28	20 833
Perinatal mortality	3.72	-1.27 to 8.72	0.14	LLR	42	31 701
Perinatal mortality	3.39	-0.95 to 7.73	0.13	LLR	56	42 706
M-zero municipalities						
Stillbirth	0.06	-0.06 to 0.18	0.32	LLR	28	778
Stillbirth	-2.52	-7.46 to 2.42	0.32	LLR	42	1 163
Stillbirth	-3.09	-9.16 to 2.97	0.32	LLR	56	1 585
Perinatal mortality	13.26	-4.10 to 30.61	0.13	LLR	28	778
Perinatal mortality	9.00	-5.63 to 23.64	0.23	LLR	42	1 163
Perinatal mortality	5.52	-8.91 to 19.94	0.45	LLR	56	1 585

** p<0.01, * p<0.05

Results of the falsification tests using different bandwidths (28, 42 and 56 days) and local linear regressions (LLR). We observe no statistically significant effects of the ceasefire on pregnancy outcomes in M-other and M-zero municipalities (as expected). M-zero: pregnant women in municipalities with no conflict event recorded during the 2000-2017 period (whether involving FARC or not). M-other: pregnant women in municipalities that did not belong to the other three groups ITT: Intention-to-treat effect per 1 000 pregnancies. 95% CI: 95% Confidence Interval

Figure 1. Distribution of categories of municipalities in Colombia, according to FARC presence between 2000 and 2017

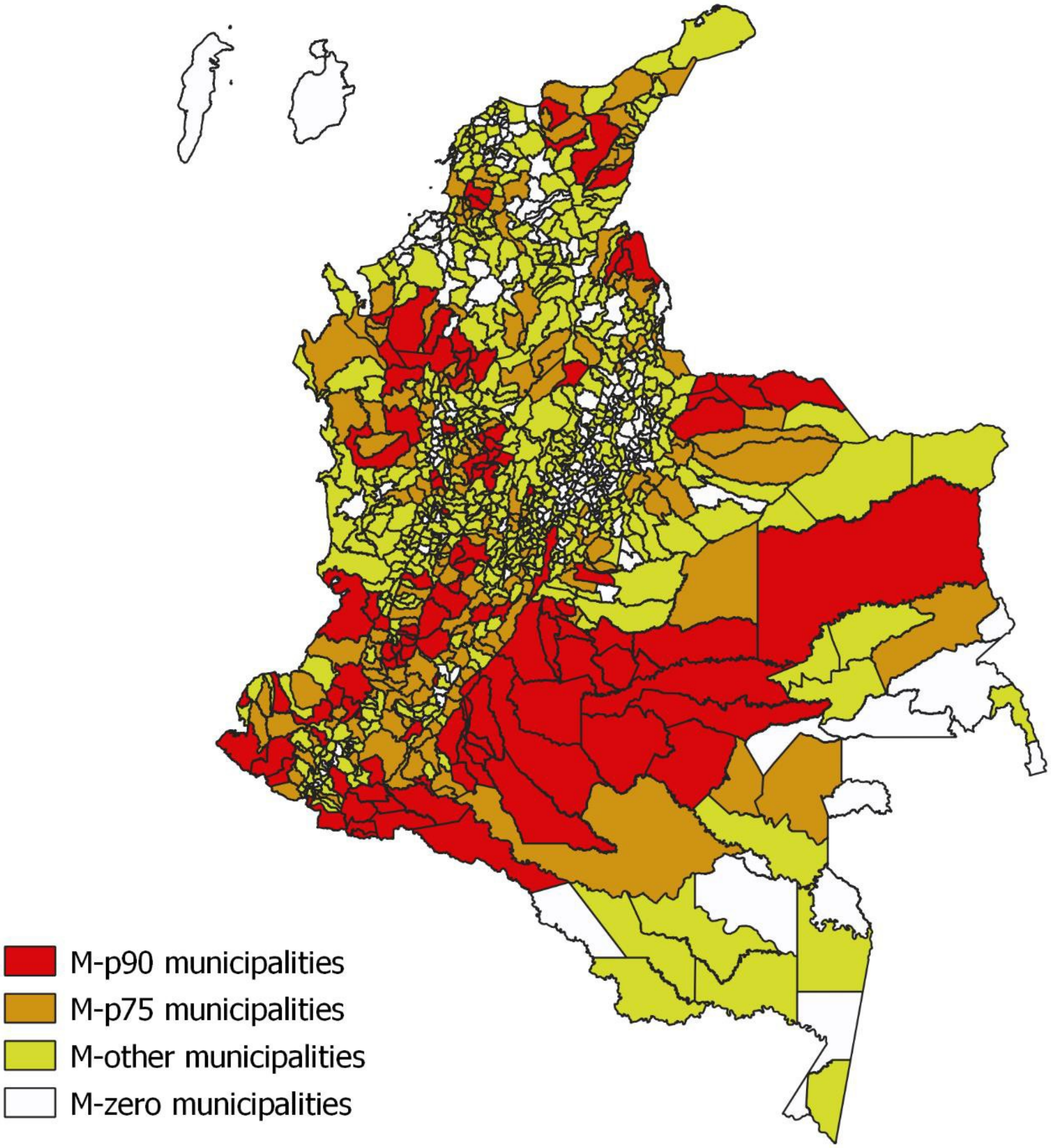

Classification of Colombian municipalities according to the distribution of the total number of conflict events involving FARC for the years 2000 through to 2017. Four categories of municipalities: 1) M-p90: municipalities with total number of FARC-related violent events above the 90th percentile of the distribution of these events (red); 2) M-p75: municipalities with total number of FARC-related events above the 75th percentile (orange); 3) M-zero: municipalities with no conflict event recorded during the entire 2000-2017 period (whether involving FARC or not) (white); and 4) M-other: municipalities that did not belong to any of the other three groups (yellow).

Figure 2. Effects of the July 20th, 2015 and August 28th, 2016 ceasefires on the exposure to total conflict events during pregnancy: Colombia and categories of municipalities (RD plots)

A

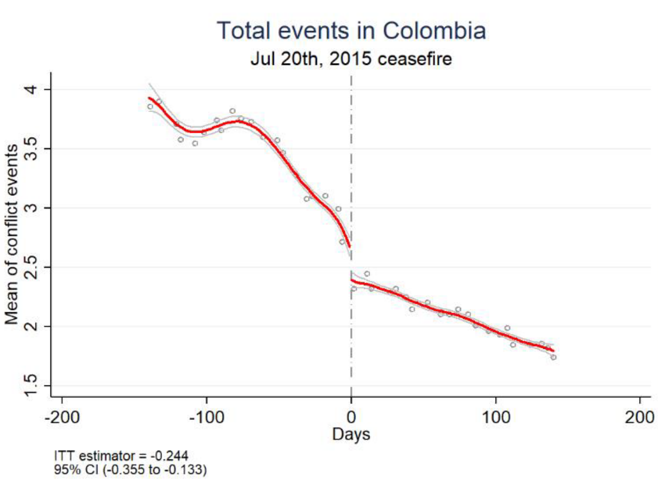

E

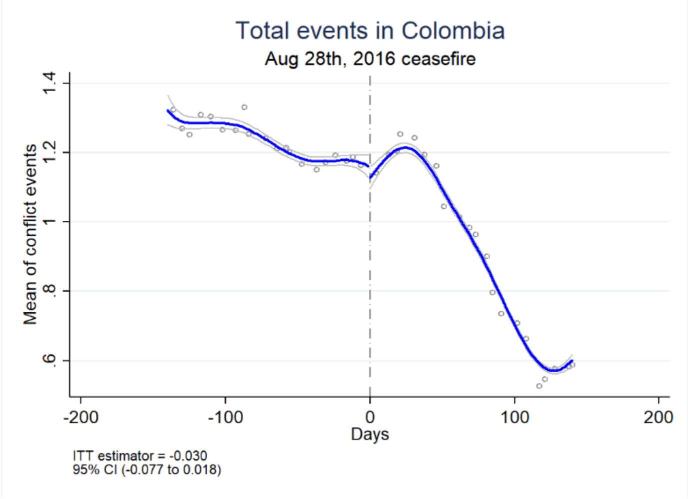

B

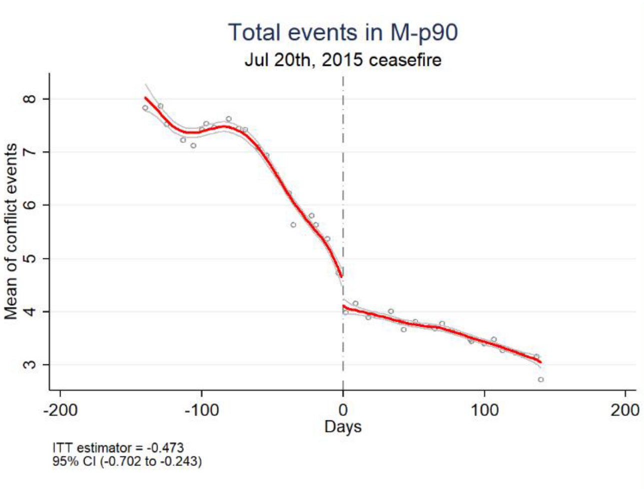

F

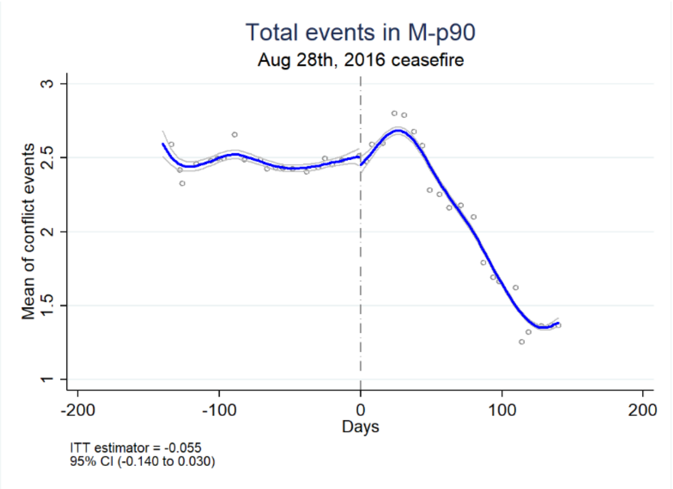

Figure 2 (Continued).

C

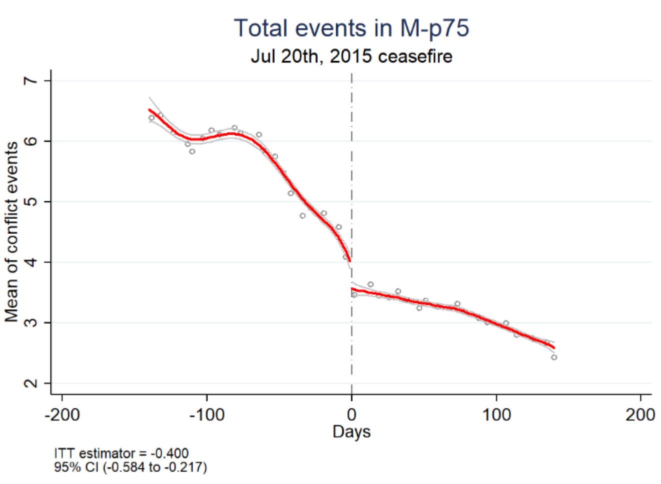

G

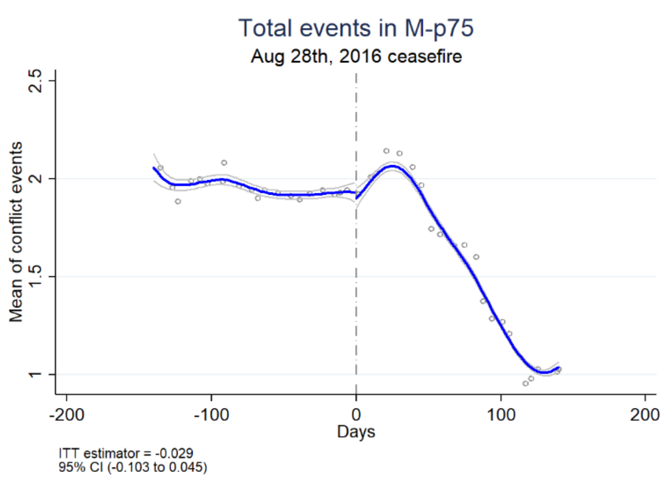

D

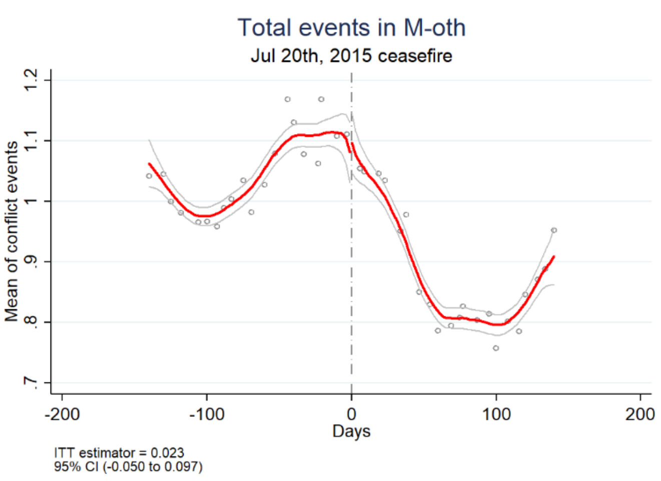

H

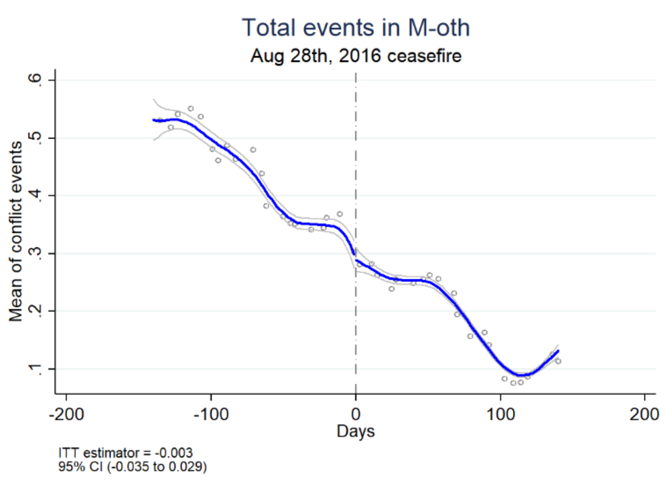

Effects of the July 20th, 2015 and August 28th, 2016 ceasefires on the exposure to total conflict events during pregnancy (i.e. events related to any armed group). Local Linear Regressions and 95% CI. A, B, C and D show the effects of the July 20th, 2015 ceasefire (red lines). E, F, G and H show the effects of the August 28th, 2016 ceasefire (blue lines). A and E include women from all municipalities in Colombia. B and F only include women in M-p90 municipalities. C and G only include women in M-p75 municipalities. D and F only include women in M-other municipalities. We do not show graphs for women in M-zero municipalities because these women were not exposed to conflict events. The results indicate that the July 20th, 2015 ceasefire produced a significant discontinuity in the number of conflict events only for women in M-p90 and M-p75 municipalities, while the August 28th, 2016 ceasefire did not result in any statistically significant changes in exposure to conflict events for pregnant women. We use different scales on the Y-axis for more clear visualization of the estimated effects of the ceasefires, as the number of conflict events close to the August 2016 ceasefire was much lower than for the previous ceasefire. Intention-to-treat (ITT) effect estimated by regression discontinuity analysis using local linear regression and bandwidths of 56 days.

Figure 3. Effects of the July 20th, 2015 ceasefire on pregnancy outcomes

A

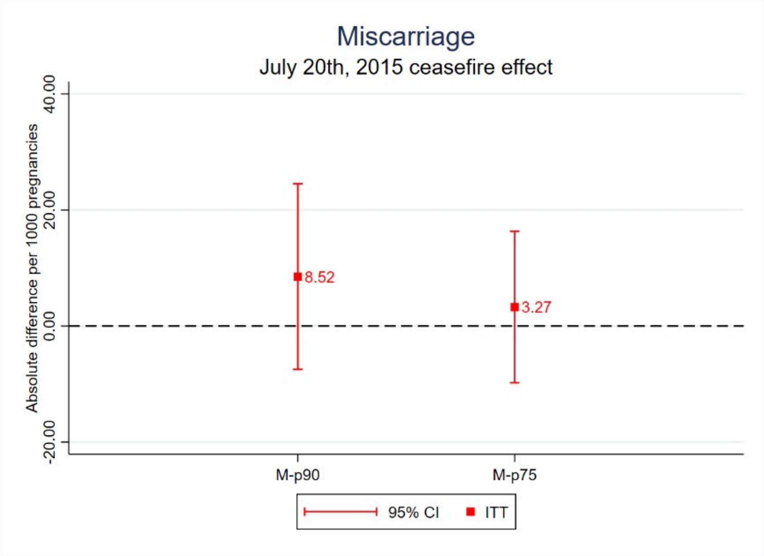

B

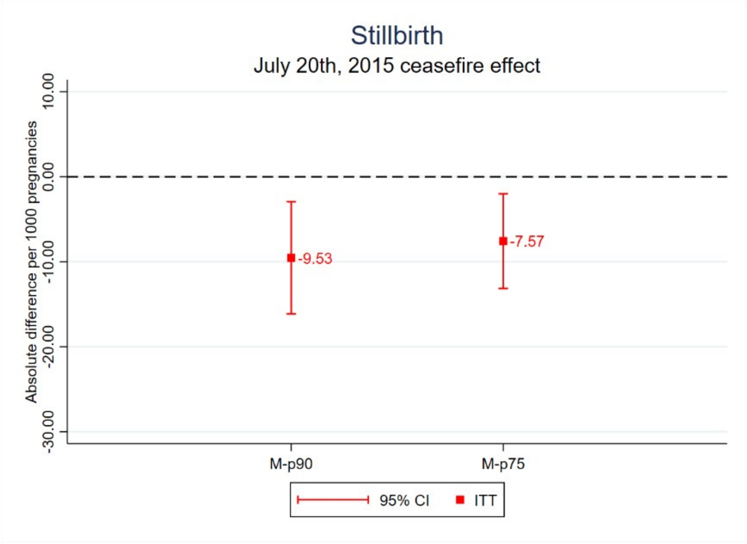

C

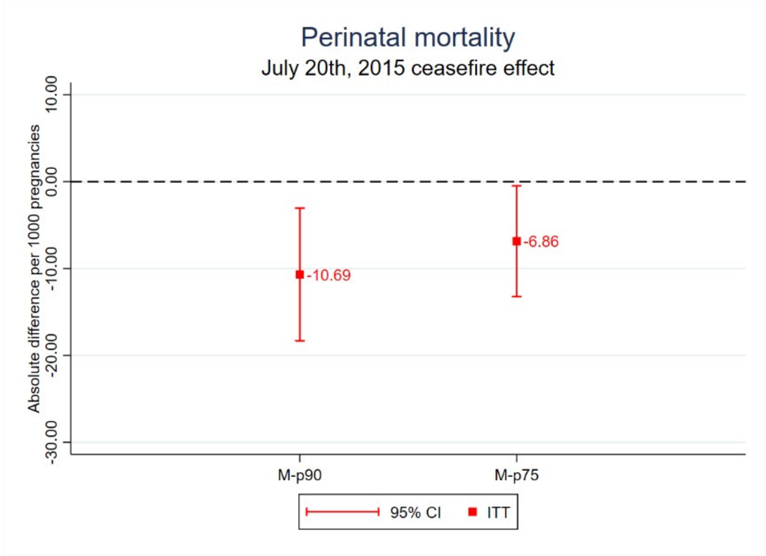

Intention-to-treat effects of the July 20th, 2015 ceasefire on the risks of miscarriage, stillbirth, and perinatal mortality, for pregnant women in M-p90 and M-p75 municipalities. Estimates obtained through regression discontinuity analysis using local linear regression and bandwidth of 28 days (See Supplementary material, section 3). We observe statistically significant effects on the risk of stillbirths and perinatal mortality, but no effects on the risk of miscarriage. The estimated effects are larger for women in M-p90 municipalities than for those in M-p75 municipalities.

Click here to access/download
Supporting Information
Supplementary material new version_final.docx
