# Supplementary material for: Conflict violence reduction and pregnancy outcomes: A regression discontinuity design in Colombia
Source: PLoS Med. 2021 Jul 6;18(7):e1003684. doi: 10.1371/journal.pmed.1003684 (PMC8259980; doi:10.1371/journal.pmed.1003684)
Supplement: Attachment — Submitted filename: Review report_D-21-00816.pdf [file pmed.1003684.s006.pdf]

Review report

PLOS Medicine: The Effects of Conflict Violence Reduction on Pregnancy Outcomes: Evidence from a Regression Discontinuity Design in Colombia

Thank you for the invitation to review the article entitled, *The Effects of Conflict Violence Reduction on Pregnancy Outcomes: Evidence from a Regression Discontinuity Design in Colombia*

General comments

The article describes a fascinating study which sought to establish the effects of conflict violence reduction on pregnancy outcomes using a regression discontinuity design. I have made detailed notes on the annotated version of the article I uploaded as part of this review. What falls is brief comment on each section of the paper.

As a general comment I would like to congratulate the researchers on an excellent article which reports on what sounds like a highly relevant, valuable and desperately under-researched area. Commendations must also be extended for the researchers use of the RD technique to make sense of the data at their disposal. I have raised some minor questions for clarity and identified a few places in the manuscript that would benefit from an edit/review. These are really minor as overall I think this is a publication worthy piece.

Abstract

The abstract provides a comprehensive summary of the paper.

Introduction

The introduction is clear and very concise. I have raised a number of questions in the annotated copy the article that I would like the authors to consider in their final resubmission. These pertain to requiring some more detail and clarity on certain statements made and some suggestions for editorial corrections.

The introduction of the article sets up a good case for the subsequent study.

Methods

The method section is really well done, I found the authors ability to communicate complex methods a real strength of this paper. Again here I have included a few questions for clarity and fuller explanation. A key requirement for revision must be a clearer articulation of what explore to violence means in this study. The study is premised this construct but really little attention is paid to defining

in this paper. In order to full appreciate the results of the study this must be amended before publication.

Some of the background information int his section like exposure to violence and the explanations of the ceasefire could appear in the introduction. It is not clear why this material is located in method section except to explain the parameters for the data coding and analysis.

Results

The results are presented in clear and comprehensive way. The figures were helpful in providing graphic representations of the findings in a digestible manner. The results were fascinating. The opportunities of using the RD method are beautifully displayed in these results. Of course the results require good data.

Please see the annotated text for some questions that I think must be addressed before a final submission.

Discussion/strengths and limitations

Again well done for the most part but I do have some questions requiring attention in these sections too.
