# Supplementary material for: Conflict violence reduction and pregnancy outcomes: A regression discontinuity design in Colombia
Source: PLoS Med. 2021 Jul 6;18(7):e1003684. doi: 10.1371/journal.pmed.1003684 (PMC8259980; doi:10.1371/journal.pmed.1003684)
Supplement: Attachment — Submitted filename: Respuestas revisores_final.docx [file pmed.1003684.s007.docx]

**-----------------------------------------------------------
Requests from the editors:**

**Please address the following editorial comments:**

**Please revise your title according to PLOS Medicine's style. Your title must be nondeclarative and not a question. It should begin with main concept if possible. "Effect of" should be used only if causality can be inferred, i.e., for an RCT. Please place the study design ("A regression Discontinuity Design”) in the subtitle (ie, after a colon).**

Response: Done. We have modified the title. The proposed title is: “Conflict Violence Reduction and Pregnancy Outcomes: A Regression Discontinuity Design in Colombia”.

**Your study is observational and therefore causality cannot be inferred. Please remove language that implies causality such as “causal evidence” or “causal inference” or “causal effect” or “causes.” Please refer to associations instead.**

Response: Done. We have modified the relevant language throughout the document. We have referred to our results as "associations", rather than "effects".

**- The Data Availability Statement (DAS) requires revision. For each data source used in your study:
a) If the data are owned by a third party but freely available upon request, please note this and state the owner of the data set and contact information for data requests (web or email address). Note that a study author cannot be the contact person for the data.
b) If the data are not freely available, please describe briefly the ethical, legal, or contractual restriction that prevents you from sharing it. Please also include an appropriate contact (web or email address) for inquiries (again, this cannot be a study author).
-If you could provide some data to support the validity of their proxy method for disease diagnosis, it would significantly enhance the quality of the manuscript**

Response: We have included the following Data Availability Statement:

*The following information sources: Single Registry of Enrollees, Module ND (Registro Único de Afiliación, or RUAF), Unique Affiliation Database (Base de Datos Única de Afiliación, or BDUA), and Calculation Study of the Capitation Unit Database (Base del Estudio de Suficiencia de la Unidad Por Capitación, or UPC) are administered by the Colombian Ministry of Health and Social Protection. These databases are freely available upon request to the Technology of the Information and Communication Office of the Colombian Ministry of Health and Social Protection through the e-mail:* [*correo@minsalud.gov.co*](mailto:correo@minsalud.gov.co)

*The source of information about armed conflict events was the National Center for Historical Memory (Centro Nacional de Memoria Histórica, or CNMH) dataset, which is publicly available at* [*http://micrositios.centrodememoriahistorica.gov.co/observatorio/portal-de-datos/el-conflicto-en-cifras/#base-de-datos*](http://micrositios.centrodememoriahistorica.gov.co/observatorio/portal-de-datos/el-conflicto-en-cifras/#base-de-datos)*. We downloaded the dataset on December 8th, 2018.*

**Please ensure that the study is reported according to the STROBE guideline, and include the completed [STROBE or other] checklist as Supporting Information. When completing the checklist, please use section and paragraph numbers, rather than page numbers. Please add the following statement, or similar, to the Methods: "This study is reported as per the Strengthening the Reporting of Observational Studies in Epidemiology (STROBE) guideline (S1 Checklist).",Please report your study according to the relevant guideline, which can be found here:** [**http://www.equator-network.org/**](http://www.equator-network.org/)

Response: we have included the S1 Checklist and the following statement (lines 159-161):

*This study is reported as per the Strengthening the Reporting of Observational Studies in Epidemiology (STROBE) guideline (S1 Checklist).*

**In the abstract Methods and Findings:**

**-Please ensure that all numbers presented in the abstract are present and identical to numbers presented in the main manuscript text.**

**-Please include the actual amounts of relevant outcomes**

**- Please quantify the main results (with both 95% CIs and p values).**

**- In the last sentence of the Abstract Methods and Findings section, please describe the main limitation(s) of the study's methodology.**

Response: We have presented all the numbers in the abstract in an identical way to what is described in the text, the graphs, and the tables. We have included the 95% confidence intervals and the p-value. We have included the main limitations of the study in the last sentence of the Abstract Methods and Findings section. (Lines 46-47). We have included the following sentence:

*The main limitations of the study are the retrospective nature of the information sources and the potential for conflict exposure misclassification.*

**Abstract summary - At this stage, we ask that you include a short, non-technical Author Summary of your research to make findings accessible to a wide audience that includes both scientists and non-scientists. The Author Summary should immediately follow the Abstract in your revised manuscript. This text is subject to editorial change and should be distinct from the scientific abstract. Please see our author guidelines for more information:** [**https://journals.plos.org/plosmedicine/s/revising-your-manuscript#loc-author-summary**](https://journals.plos.org/plosmedicine/s/revising-your-manuscript#loc-author-summary).

Response: We have now included an “Abstract summary” section with contents and placement as advised (lines 57-88).

*Abstract summary*

*Why was this study done?*

- *Evidence is lacking about the links between exposure to armed conflict during pregnancy and the risks of miscarriage, stillbirth, and perinatal mortality*
- *Only seven observational studies with a high risk of bias have examined the relationship between conflict exposure and miscarriage, stillbirths, or perinatal mortality. These studies found no evidence of causal effects of conflict exposure on any of these pregnancy outcomes.*
- *There were no published studies examining the relationship between exposure to conflict violence during pregnancy and the risk of miscarriage, stillbirths, or perinatal mortality in the context of a chronic armed conflict.*

*What did the researchers do and find?*

- *We exploited a discontinuity in the intensity of conflict violence across Colombian municipalities, which was generated by a ceasefire during peace talks between the government and FARC, as an exogenous change in conflict exposure for pregnant women. This natural experiment allowed us to implement a regression discontinuity estimation approach to determine the association between reduced exposure to conflict violence and the risks of miscarriage, stillbirth, and perinatal mortality.*
- *We found that the fall in the intensity of conflict violence to which women were exposed at the beginning of their pregnancies is significantly associated with decreases in the stillbirth and perinatal mortality rates. We found no associations with the risk of miscarriage.*
- *The estimated reductions in stillbirth and perinatal death rates associated with reduced conflict exposure were larger for women living in municipalities where the intensity of FARC-related violence was historically greater.*

*What do these findings mean?*

- *Our findings highlight how pregnant women are particularly vulnerable amid armed conflicts and indicate the need for countries to take specific actions to protect these women since the early stages of pregnancy.*
- *For Colombia, our results offer supportive evidence about the potentially large health benefits arising from the peace process, which can be used to inform current discussions around the political sustainability of this process in the next years.*

**Introduction section: please provide a clear statement of hypothesis at the end of the introduction.**

Response: We have included our hypothesis at the end of the introduction (Lines 130-131). We have included the following sentence:

*Our hypothesis is that reductions in the exposure to armed conflict during pregnancy are associated with reductions in the rates of miscarriages, stillbirths, and perinatal mortality.*

**Did your study have a prospective protocol or analysis plan? Please state this (either way) early in the Methods section.**

**a) If a prospective analysis plan (from your funding proposal, IRB or other ethics committee submission, study protocol, or other planning document written before analyzing the data) was used in designing the study, please include the relevant prospectively written document with your revised manuscript as a Supporting Information file to be published alongside your study, and cite it in the Methods section. A legend for this file should be included at the end of your manuscript.
b) If no such document exists, please make sure that the Methods section transparently describes when analyses were planned, and when/why any data-driven changes to analyses took place.
c) In either case, changes in the analysis-- including those made in response to peer review comments-- should be identified as such in the Methods section of the paper, with rationale.**

Response: The study protocol was approval by the Research and Institutional Ethics Committee of the School of Medicine of Pontificia Universidad Javeriana. This protocol is in Spanish, if necessary, we can translate this document into English. We have included the protocol and the IRB letter. We have introduced this paragraph in the Methods section (Lines 134-137):

*Ethics*

*This study was granted IRB ethical approval by the Research and Institutional Ethics Committee of the School of Medicine of Pontificia Universidad Javeriana (Minutes No. 10/2018), Colombia. The study protocol is presented in the Supplementary material.*

**-In statistical methods, please refer to any post-hoc corrections to correct for multiple comparisons during your statistical analyses. If these were not performed please justify the reasons. Please refer to our statistical reporting guidelines for assistance (**[**https://journals.plos.org/plosone/s/submission-guidelines.#loc-statistical-reporting**](https://journals.plos.org/plosone/s/submission-guidelines.#loc-statistical-reporting)**)**

Response: We have not undertaken any post-hoc corrections to correct for multiple comparisons, because we did not perform multiple comparisons in our analyses.

**In the Methods and Results section:**

**- Please provide both adjusted and unadjusted estimates.**

Response: The unadjusted estimates are presented in the three final rows of revised Table 1 (line 301). Adjusted estimates are included in the Results sections, tables, and figures.

**- Please provide 95% CIs and p values for all HRs.**

Response: We have included 95% CIs and p values for all estimates presented in the Results section and tables.

**- When a p value is given, please specify the statistical test used to determine it.**

Response: We have improved the description of regression discontinuity estimates in the Methods section. We included the following statement (Lines 265-268):

*This estimation strategy involves approximating the regression functions above and below the threshold (i.e., Jul/20/2015) by means of weighted polynomial regressions of order 1 (also called local linear regressions), with weights computed by applying a triangular kernel function on the distance in days of each observation to the threshold [32-34].*

We have included the following statement in lines 290-291: *“For all RD estimations we report p-values from local linear regressions with 95% confidence intervals”*

**For your figures and tables, please do the following:**

**- Please define the abbreviations such as FARC, m-P90, m-p75, M-oth, M-zero**

Response: We have now included definitions for all abbreviations in all figures and tables.

**Please include line numbers in your next draft.**

Response: We have included line numbers in the new version.

**Comments from the reviewers:**

**Reviewer #1: This well-written manuscript addresses the important topic of how reductions in conflict violence may affect pregnancy outcomes in low-resource settings with high levels of conflict violence. The authors report on data from over 3 million pregnant women in the country of Columbia between 2013 and 2017. They take advantage of a natural experiment that occurred when ceasefires with FARC occurred in 2015 and 2016 and resulted in dramatic reductions in conflict events. They utilize comprehensive administrative municipality-level data on conflict events that occurred and on adverse pregnancy outcomes (stillbirths, miscarriages, and perinatal mortality) to conduct a rigorous regression discontinuity analysis, and found that decreases in conflict events in municipalities heavily impacted by FARC-related conflict were associated with statistically significant reductions in stillbirths and perinatal mortality (but not miscarriages). The authors have done a good job of describing their methodology, assumptions, sensitivity analyses, and noting the limitations of their research. However, I had a few questions and comments as detailed below. Overall, I thought this was a fascinating analysis and an important contribution to the literature. Specific comments by section are given below.**

Response: Many thanks for your comments about the clarity, novelty, and importance of our analysis.

**Abstract:
1.       Page 2 and throughout: Given that this necessarily an observational study, it seems like the authors should avoid language such as "the reduction in average exposure to conflict violence resulted in…." throughout the paper. There is not definitive evidence that the reduction in violence caused these outcomes. However, I do recognize that this may be the best evidence we can get on this question.**

Response: We greatly appreciate your comment. Although we do believe that the evidence presented in our paper is statistically robust (indeed the best we can get on this topic) and likely to reflect (even if, at instances, partial) causal relationships, we agree with the Reviewer’s and editorial advice about being more cautious with the relevant language and have thus avoided causal language throughout the document in the new version. We have referred to "associations" rather than "causal estimates" or "effects".

**Introduction:
2.      Page 5:  Were there other events or trends going on simultaneously in the country during this period that could have also contributed to improved pregnancy outcomes?  Campaigns to increase birth in a health facility with a skilled attendant?  Increases in maternal education? Changes in the economy?**

Response: We have been very careful to identify relevant events, such as those mentioned by the Reviewer, that could have occurred simultaneously around the implementation of the July 20th, 2015 ceasefire (which was the one we found to be associated with pregnancy outcomes). To our knowledge, there were no other specific events (e.g., maternal health interventions, macroeconomic shocks) taking place that could have been plausibly associated with these outcomes. Moreover, even if we failed to identify such an event, it is very unlikely that this could have influenced our results. To threaten the validity of our estimates, events, or trends such as those described by the Reviewer would need to have been implemented or changed suddenly, discontinuously in the neighborhood of the threshold (i.e., one month around July 20th, 2015) and only in the municipalities historically most affected by the FARC.

In the context of our study, exposure to conflict events during pregnancy is the ‘treatment’ of interest, with such variation in exposure shifting discontinuously at a threshold (July 20th, 2015 ceasefire) of the distribution of the continuous conception dates (measured in days). Because this assignment rule generates an exogenous discontinuity in the probability of exposure among women with essentially identical conception dates on either side of the threshold (identical in the limit, as one approaches the threshold), treatment assignment is ‘as-good-as-random’ for women in the neighborhood of the threshold, enabling robust statistical inference. Importantly, the validity of inference in this framework relies on the assumption of continuity of the conditional expectation functions for each of the potential outcomes at the threshold, which implies no-manipulation of the running variable (i.e., conception dates) and continuity of baseline variables at the threshold. For an event like the implementation of a maternal health campaign to bias our estimations, it would be necessary that the event was to be adopted suddenly so as to discontinuously change the distribution of potential outcomes in the neighborhood of the ceasefire date, i.e. changing the characteristics of women starting pregnancies after the ceasefire date, with these changes happening specifically for the particular groups of women under comparison (e.g. those living in M-p90 municipalities). This would be reflected in observed discontinuities in conception dates and/or baseline characteristics at the ceasefire date threshold. Both the no-manipulation and continuity of baseline variables assumptions can be tested formally with the data at hand, as we do extensively in the paper and provide additional empirical support in the Supplementary Material. In our various tests we find consistent evidence that the distributions of observed conception dates and key observable confounders are continuous at the threshold, thus also supporting the continuity of unobservable and other observable confounders at the threshold (similarly to an RCT setting) [1–3], implying in turn that any unobserved interventions or events that might have taken place around the relevant ceasefire date should not invalidate our results.

We have added a paragraph to clarify this point in a summarized manner in the Discussion (sub-section “Strengths and limitations of this study”, lines 450-462). We have included the following paragraph:

*There could have been, of course, several other changes in socio-economic factors or events (e.g., local health interventions) taking place during the study period, which could influence pregnancy outcomes in general. However, to threaten the validity of our estimates, given the characteristics of our RD estimation design, events or trends such as those described would need to have been implemented or changed suddenly, discontinuously in the neighborhood of the threshold (i.e. one month around the July 20th, 2015 ceasefire), and specifically for the particular groups of women under comparison (e.g. those living in M-p90 municipalities), in such a way to then introduce discontinuities in the observed distribution of conception dates and/or baseline characteristics at the ceasefire threshold. Yet in our extensive statistical testing of both the no-manipulation and baseline variable continuity assumptions, we found consistent evidence that the distributions of observed conception dates and key observable confounders are continuous at the threshold, thus also supporting the continuity of unobservable and other observable confounders at the threshold [28,29].*

**Methods:
3.      Page 10:  Which observable baseline characteristics of women before and after the ceasefire were tested statistically? These should be specified.**

Response: Thank you for this comment. We have included the following sentence in the Methods section (Lines 277-279):

*These key baseline characteristics (extracted from the birth or death certificates) included age, education level, health insurance status, marital status, and number of children.*

We also list and provide descriptive statistics of the baseline characteristics in Table 1.

**Results:
4.      Page 11:  Based on the characteristics in Table 1, I would like to know if any of the differences in other characteristics of the women are statistically significant among the municipality groups. I think it would be more informative to show all four municipality groups in these tables (not just the total, M-p90, and M-p75), so we can understand more about the differences between those municipalities who were most impacted by FARC-related conflict and those who were less impacted.**

Response: As suggested, the revised version of our paper includes an extended Table 1 (Line 301) with information about the baseline characteristics for all four municipality groups.

**Discussion:
5.       Page 15:  The results of the sensitivity test indicating that exposure very early in pregnancy is the key driver are not explained clearly enough. It is not clear to me why increasing the RD bandwidth to sizes up to two months with no changes in results would lead to this conclusion.**

Response: We appreciate the opportunity to clarify. We suggest that decreased exposure to conflict in the very early stages of pregnancy is associated with a greater decrease in stillbirth and perinatal mortality rates, because the sensitivity analyses show that the RD estimates decrease in absolute value as the bandwidth increases from 14 to 56 days (Tables A2 and A3 in Supplementary Material). For example, using a functional form of local linear regression (LLR) for M-p90 municipalities, the ITT estimators for stillbirths are -15.83, -9.53, -6.52, and -4.88, for 14, 28, 42, and 56 days, respectively. This is similar for all functional forms and for the two outcomes (stillbirths and perinatal mortality). We have revised the text in the manuscript accordingly (lines 425-427) and hope this has improved the clarity of our argument.

We included the following statement (lines 470-472):

*Nevertheless, it is noteworthy that the RD point estimates for stillbirths and perinatal mortality tend to decrease in absolute value as the bandwidth increases from 14 to 56 days (see Tables A2 and A3 in the Supplementary Material).*

**6.      Page 16:  The discussion of mechanisms of effect is much appreciated.  However, could another mechanism be related to improvements in economic activity and the socio-economic status of women due to the environment of reduced conflict?  Poverty is associated with adverse health outcomes and perhaps improvements in economic status could be another mechanism.**

Response: We agree that there are many possible channels whereby reductions in exposure to violence could affect pregnancy outcomes, among them, socio-economic changes. However, given the characteristics of the discontinuous regression analysis that we have previously mentioned, these possible mechanisms would need to have changed abruptly in a very short period (around 28 days), which is arguably unlikely for population-level socioeconomic changes. We acknowledge, however, that we are unable to investigate directly with our dataset further potential mechanisms at play, an aspect that we also highlight in the revised text discussing the limitations of our study (lines 475-477).

We included the following statement (lines 475-477):

*One limitation of our study is that, with the data available, we were unable to undertake an in-depth investigation of possible mechanisms linking reductions in conflict exposure to reduced risks of stillbirth and perinatal mortality.*

**7.      Page 17: Another limitation is that we do not know for certain whether women were directly exposed or even aware of the conflict events that occurred in their municipalities.**

Response: We thank the Reviewer for making this point, with which we agree. We have included this possible limitation in the discussion section (Lines 520-522). We included the following statement:

*Similarly, we cannot account in our empirical analysis for potential differences in the intensity of individual conflict exposure, or in the knowledge about conflict events, among pregnant women living in the same municipality*

**Tables and figures:**

**8.       I suggest including all four municipality groups in Tables 1 and 2, for full transparency and information.**

Response: We have modified Tables 1 and 2 accordingly, including information for all four municipality groups.

**9.      Table 2:  Please define "Selective murder".  Overall, it would be helpful to define all the conflict events in footnotes to the table.  What is the difference between a terrorist attack and a massacre?**

Response: We have included a new section (2.1. Definitions of types of armed conflict events) in the Supplementary Material, where we outline the definitions of all types of conflict events in the dataset we used, according to the official data publishing source. We have mentioned it in the footnote of Table 2.

**10.     Figure 2:  The changing of the scales on the Y-axis for July 2015 and August 2016 is confusing and makes it appear that there were dramatic and similar decreases for both ceasefires. Use of the same scales would make clearer the reason for the lack of effects for the August 2016 ceasefire.  This is explained in a footnote, but may be missed by many readers.**

Response: We have changed the scales on the Y-axis of the Figure 2.

**Reviewer #2:**

**This paper uses various sources of data to examine the relationship between violence and pregnancy outcomes in Colombia. I was asked for a statistical report and I interpret that to include all aspects of the design and conduct of the study.**

**Points of detail**

**Page 3 Strictly speaking the percentile is the value at the category boundary but it is hard to find a suitable alternative word here.**

Response: We agree and have modified the relevant text as follows (Lines 35-41):

*The reduction in average exposure to conflict violence was in turn associated with a decrease of 9.53 stillbirths per 1 000 pregnancies (95% CI -16.13 to -2.93; p=0.005) for municipalities with total number of FARC-related violent events above the 90th percentile of the distribution of FARC-related conflict events, and a decrease of 7.57 stillbirths per 1000 pregnancies (95% CI -13.14 to -2.00; p=0.01) for municipalities with total number of FARC-related violent events above the 75th percentile of FARC-related events.*

**Page 8 If I understand this correctly M-p90 contains regions with many events attributed to FARC irrespective of the activities of the other two organisations shown in Figure A3: ELM and AUC. Perhaps that could be clarified? More importantly we do not seem to have a justification for the choice of those particular percentages.**

Response: Theoretically, ceasefires declared with/by the FARC should have a greater impact on armed conflict violence in the municipalities where the FARC had a greater historical presence. Municipalities where the FARC did not have a presence should not have experienced a decrease in armed conflict events after the ceasefire with the FARC. We use the distribution of armed conflict events related to the FARC, by municipality, between the years 2000 and 2017, in order to identify the historical presence of the FARC before the start of the Havana talks. Our intention was to generate a classification of municipalities that would illustrate municipal heterogeneity with respect to FARC-related violence levels, in order to investigate the relationships between said heterogeneity and the local responses of violence levels to the ceasefire. We considered that four categories of municipalities could achieve this objective without compromising sample sizes for estimation purposes.

We did not perform a formal statistical analysis to make the decision about the percentiles used to define the municipality categories. However, we made an attempt to explore an alternative, finer-grained classification of municipalities, which could also in principle enable us to meet our objective of investigating a dose-response relationship. We generated a classification based on deciles of the distribution of FARC-related armed conflict events between 2000 and 2017. Yet this has resulted in small effective sample sizes of pregnancies, in each cell, preventing us from performing reliable RD estimations of decile-specific treatment effects to be compared with those in the main analysis, or indeed of reliably trying to identify a dose-response pattern.

We present in the table below the distribution of the number of pregnant women in each of the deciles. We observe that the effective sample size of pregnancies for the analysis (around the ceasefire threshold) is much smaller for the decile categories (except for the 90th percentile) compared to the sample sizes for the categories currently used (M-p90, M-p75, M-other, and M-zero). Due to the small sample sizes generated by this alternative classification, running our RD models for decile categories leads (as expected) to unreliable and imprecise estimates of standard errors and treatment effects. For example, the total number of stillbirths using the effective sample around July 20th, 2015 ceasefire (28 days) within each category is 20, 15, 20, 34, 29, 36, and 22 for the percentiles 10th/20th, 30th, 40th, 50th, 60th, 70th, and 80th, respectively.

We trust – and hope that the Reviewer agrees – that the analytical reasons described above provide a sound rationale for our choice of municipality categories in the main article.

| Percentile | Percentile value | Number of observations | |
| --- | --- | --- | --- |
|  |  | Full sample  (N= 3,253,656) | Effective sample around July 20th, 2015 ceasefire (BW=28 days)  (N= 48,458) |
| 10^th^ | 0 | 341,973 | 4,937 |
| 20^th^ | 0 |  |  |
| 30^th^ | 1 | 204,771 | 2,939 |
| 40^th^ | 3 | 215,028 | 3,178 |
| 50^th^ | 7 | 209,866 | 3,012 |
| 60^th^ | 12 | 315,734 | 4,698 |
| 70^th^ | 22 | 345,176 | 5,065 |
| 80^th^ | 37 | 332,337 | 4,950 |
| 90^th^ | 83 | 1,288,771 | 19,679 |

Note: 10th and 20th percentile have same value therefore we have combined both cells in the table.

We have included the following statement (lines 217-220):

*We chose these categories to achieve the objective of identifying municipal heterogeneity with respect to FARC-related violence levels, in order to investigate the relationships between said heterogeneity and the local responses of violence levels to the ceasefire, without compromising sample sizes for estimation purposes.*

**Page 10 I think the term local linear may need an extra sentence or two of explanation. The notion here may be more familiar to economists than people in health. People may, for instance, be surprised at seeming a linear model which involves estimating coefficients described as non-parametric. A bit more explanation of the linear, quadratic and cubic models used as sensitivity analyses would not be a bad idea either. I assume they are separate global fits to all the data before the discontinuity and to all the after data**

Response: We thank the Reviewer for this comment and follow their advice to clarify further. We have included further explanatory text in relevant places with accompanying supporting references. In particular, we have included:

In lines 265-268:

*This estimation strategy involves approximating the regression functions above and below the threshold (i.e., Jul/20/2015) by means of weighted polynomial regressions of order 1 (also called local linear regressions), with weights computed by applying a triangular kernel function on the distance (in days) of each observation to the threshold [32-34].*

In lines 283-285:

*The order of the polynomial refers to the highest exponent associated with the running variable of the discontinuous regression (i.e., the distance in days from each observation to the ceasefire date) [27,35].*

**Page 12 The miscarriage results are important so I think the numerical results should be shown here.**

Response: We agree with the Reviewer’s suggestion and have included the following information in the main text (lines 359-362):

*We did not find statistically significant associations with respect to miscarriages: point estimates were -8.52 (95% CI -7.47 to 24.52; p=0.30) in M-p90 municipalities, and -3.27 (95% CI -9.78 to 16.32; p=0.62) in M-p75 municipalities.*

**Page 15 This gives the bandwidth as one month but Figure 2 in the caption states 56 days. I can guess how these could be reconciled but perhaps a clarification might be in order.**

Response: We clarify that the optimal bandwidth calculated and therefore used for the RD estimates of changes in *conflict events* (Figure 2) was 56 days. Yet the optimal bandwidth calculated and used in the case of RD estimates for the *main outcomes* (miscarriage, stillbirths, and perinatal mortality) was 28 days. To be on the safe side, we show and discuss in the main text and Supplementary Material that we also tested various alternative bandwidth sizes of up to 56 days (inclusive) for the RD estimations related to the main outcomes, with no material changes in our conclusions.

To clarify the above, we have included the following information in the text (line 467-470):

*Although our main estimation results for pregnancy outcomes are based on the same optimal RD bandwidth of one month [31], we tested various alternative bandwidth sizes of up to two months, with no material changes in our conclusions.*

Furthermore, we have improved the description of the bandwidth choices in the captions of Figures 2 and 3.

**Page 24, Table 2 I found this hard to grasp. As I understand it FARC was the dominant force during the armed struggle. I do not follow Colombia that closely in our media but it was the only organisation which was reported on here so I accept it was the major one. If the events here are all conflict related then how does the FARC value form such a small part of the total? If the bulk of these relate to the Colombian army then that could be mentioned.**

Response: We appreciate the opportunity to clarify this point. There were various armed groups that were active during the Colombian conflict, with the FARC being historically the group with the greatest presence and involvement in violent actions prior to the Havana talks. The dialogues in Havana were exclusively conducted with the FARC and therefore, during the study period (2013-2017), the total number of conflict events (Figure A4) and the number of events related to the FARC (Figure A5) decreased considerably. Table 2 presents information about all the conflict events in the country *during the study period (2013-2017)*; therefore, given that the Havana talks started in 2012, the events related to the FARC did not have a large participation in the total number of events during most of the study period. As the Reviewer mentions, most of the conflict events taking place after the start of the peace talks with the FARC involved the Colombian armed forces, as the latter continued to fight the other rebel armed groups. We have now included text to clarify this matter in the caption of Table 2.

**Figure A7 and A8 Is there any explanation for the consistent fall in maternal age during the before ceasefire period? Is this a possible confounding variable?**

Response: The fall mentioned by the Reviewer amounts to approximately one year in the average maternal age in the 100 days prior to the ceasefire. We do not have a ready explanation for this average decrease. However, this fall in average maternal age should not confound or otherwise threaten the validity of our results. As we show graphically in the paper, there is no perceived discontinuity in maternal age just around the threshold (ceasefire of July 20, 2015). Additionally, from a more formal statistical point of view, the corresponding RD estimation does not show a statistically significant difference or discontinuity in maternal age around the threshold (0.14, 95% CI -0.15 to 0.42 in M-p90 municipalities; and 0.18, 95% CI -0.06 to 0.43 in M-p75 municipalities).

**Table A2 Although using different bandwidths is a useful supplementary analysis I would question whether fitting a global polynomial model is worth doing. The regression discontinuity design, if I understand it correctly, places most emphasis on the time period closest to the discontinuity. Following the arguments in Magee (1998) a polynomial is not the function which has that characteristic as it is influenced by the observations throughout the range.**

Response: We agree with the Reviewer. A discontinuous regression analysis should give more weight to observations that are very close to the threshold. The most up-to-date theoretical evidence about regression discontinuity design shows that the best RD estimates (i.e., with the lowest probability of bias) are obtained from a first-order polynomial regression with an optimal bandwidth, using a kernel function that generates a weighting of the observations according to their distance from the threshold [1,4–6]. The most commonly used kernel function is a triangular function. As Magee (1998) argues, higher-order polynomials are generally not suitable. This has also been shown by Gelman and Imbens (2014) [7]. We have opted for keeping a battery of tests with different bandwidths and functional forms (including linear models) in our analysis for the sake of completeness and to demonstrate the stability of our results. We have described, in a previous response, the additional explanatory text that we have included in relevant places about this issue, with accompanying supporting references (lines 265-268).

**Summary**

**Mostly for clarification. The justification of the choice of 90 and 75 is important.**

Response: We have explained in a previous response the rationale for the choice of the municipal categories analyzed, as well as the alternative categorization explored. We hope this clarifies the matter raised by the Reviewer.

**References**

**L Magee. Nonlocal behavior in polynomial regressions. American Statistician, 52:20{22, 1998.**

**Reviewer #3:**

**Thank you for the opportunity to read the manuscript, "The Effects of Conflict Violence Reduction on Pregnancy Outcomes: Evidence from a Regression Discontinuity Design in Colombia". This interesting piece of work uses a quasi-experimental design to explore the causal effect of exposure to violence on pregnancy outcomes. The study benefits from a large sample and an interesting use of administrative data. The sensitivity analyses conducted (e.g., bandwidths and a parametric approach with first-, second-, and third-order polynomial specifications, placebo effects) are a valuable strategy to validate the RD design.**

Response: We appreciate the Reviewer’s comments.

**Abstract
-       In the methods and findings paragraph, the authors indicate: "We found that the July 2015 ceasefire reduced the average number of conflict events to which women were exposed during pregnancy in their municipalities of residence (absolute effect -0·20; 95% CI -0·33 to -0·08)". I suggest specifying the effect size index reported here. Is it a difference in means?**

Response: We thank the Reviewer for this suggestion. We have modified the relevant sentence (Lines 31-34):

*“We found that the Jul/20/2015 ceasefire was associated with a reduction of the average number of conflict events (from 2.64 to 2.40) to which women were exposed during pregnancy in their municipalities of residence (mean differences -0.24; 95% CI -0.35 to -0.13; p<0.001).”*

In the sentence above we corrected a typo in the point estimate given in the original manuscript: the correct value is indeed -0.24. We now have the correct point estimate in the summary, main text, tables, and graphs.

**Introduction
-       Page 4, end of the second paragraph. The authors mention previous research testing the associations between conflict exposure and miscarriages, stillbirth and perinatal mortality. It is stated that all previous evidence is based on observational data. Did any of the studies involve a quasi-experimental design (propensity score matching, interrupted series analysis?  If so, I would discuss the limitations of previous quasi-experimental evaluations.**

Response: Yes, a systematic review identified seven different previous studies. Due to the point raised by the Reviewer, we have now included the explanation in the footnote 1 (line 111):

*“Five studies are before-and-after analyses without an adequate statistical control for observable and unobservable confounders [14–17,19]. A further study is a retrospective cohort analysis that includes a multivariate regression model to identify associations [20], whereas another study relies on before-and-after comparisons within a simple fixed-effects panel analysis [18]*.”

**Methods and results**

**-       Please add some details on how the "probable conception date" was calculated.**

Response: The probable date of conception was estimated from the difference between the date of delivery (or death in the case of stillbirths) and the gestational age reported in the birth or death certificates.

We have included the following sentence (Line 147-149):

*The probable date of conception was estimated using the difference between the date of delivery (or death in the case of stillbirths) and the gestational age reported in the birth or death certificates.*

**-       The authors assert that data were extracted from birth/death certificates. However, Table 1 in the findings section describes demographic characteristics of participants such as age, education, marital status, health insurance, number of children, among others. It is not clear if all this information was extracted from the birth/death certificates or from a different source of administrative data. If a different source of data was included in the study, was that data affected by missingness? Did you explore patterns of potential missing data? Please clarify.**

Response: All baseline data, including about demographic characteristics, was obtained from the birth/death certificates, exclusively. We have clarified this on the main paper (Lines 277-279). Additionally, we have included in the footnote to revised Table 1 the information about missingness for all variables. As can be seen, the proportion of missing values is very low.

We included the following text in the footnote to revised Table 1:

*The number (percentage (%)) of missing values for each variable is: Age = 0 (0.00%), health insurance = 43 (0.00%), education = 125 334 (3.85%), married = 0 (0.00%), number of children = 29 (0.00%), prenatal care utilization = 174 147 (5.35%), pregnancy outcomes = 0 (0.00%).*

**-       The description of the RD design and the sensitivity analyses are well developed and offer to the reader enough elements to evaluate the quality of the outcomes.**

Response: We thank the Reviewer for their comment.

**-       It might help to describe in more detail the variables analysed in the study, for example, exposure to conflict events such as selective murder or sexual violence. How are these concepts operationalised?**

Response: We have included a new section (2.1. Definitions of types of armed conflict events) in the Supplementary Material, where we outline the definitions of all types of conflict events in the dataset we used, according to the official data publishing source. We have mentioned it in the footnote of Table 2.

**Discussion
-       The discussion on the role of stress as a potential mechanism linking exposure to violence and stillbirth and perinatal mortality deserves more elaboration. Given the aims of the Journal, it would be useful to further elaborate the biological mechanisms underpinning the effects of violence and adversity exposure on maternal and birth outcomes.**

Response: This is a great suggestion. We have now fleshed out this aspect of the discussion in the main text (lines 478-480), focusing on evidence pointing to a dysfunction of the hypothalamic-pituitary-adrenal axis as the main mechanism by which higher stress levels influence pregnancy outcomes [8–11].

**Reviewer #4: Congratulations on both an expertly implemented study using a novel analytical tool (RD) and for writing up your work in an accessible and enjoyable paper. I think this work is publication worthy but there are some minor errors I think need to be attended to. These I have captured on my annotated document attached. Good luck with your publication and follow up studies in this under-researched field.**

Response: We really appreciate the Reviewer’s very positive assessment of our paper’s novelty and rigor.

For our revised manuscript, we have sought to take into account all the editorial/rewriting and other suggestions made by the Reviewer in their annotated PDF. Our resulting (tracked) changes are located in the corresponding places of the original text where the Reviewer has indicated them.

**Review report**

**PLOS Medicine: The Effects of Conflict Violence Reduction on Pregnancy Outcomes: Evidence from a Regression Discontinuity Design in Colombia**

**Thank you for the invitation to review the article entitled, The Effects of Conflict Violence Reduction on Pregnancy Outcomes: Evidence from a Regression Discontinuity Design in Colombia**

**General comments**

**The article describes a fascinating study which sought to establish the effects of conflict violence reduction on pregnancy outcomes using a regression discontinuity design. I have made detailed notes on the annotated version of the article I uploaded as part of this review. What falls is brief comment on each section of the paper.**

**As a general comment I would like to congratulate the researchers on an excellent article which reports on what sounds like a highly relevant, valuable and desperately under-researched area. Commendations must also be extended for the researchers use of the RD technique to make sense of the data at their disposal. I have raised some minor questions for clarity and identified a few places in the manuscript that would benefit from an edit/review. These are really minor as overall I think this is a publication worthy piece.**

Response: We very much appreciate the Reviewers’ highly positive comments.

**Abstract**

**The abstract provides a comprehensive summary of the paper.**

**Introduction**

**The introduction is clear and very concise. I have raised a number of questions in the annotated copy the article that I would like the authors to consider in their final resubmission. These pertain to requiring some more detail and clarity on certain statements made and some suggestions for editorial corrections.**

**The introduction of the article sets up a good case for the subsequent study.**

Response: As mentioned above, we have sought to take into account all the editorial/rewriting and other suggestions made by the Reviewer in their annotated PDF. Our resulting (tracked) changes are located in the corresponding places of the original text where the Reviewer has indicated them. We also appreciate the Reviewer’s comments about the clarity and conciseness of the Introduction section.

**Methods**

**The method section is really well done, I found the authors ability to communicate complex methods a real strength of this paper. Again here I have included a few questions for clarity and fuller explanation. A key requirement for revision must be a clearer articulation of what explore to violence means in this study. The study is premised this construct but really little attention is paid to defining in this paper. In order to full appreciate the results of the study this must be amended before publication.**

**Some of the background information in this section like exposure to violence and the explanations of the ceasefire could appear in the introduction. It is not clear why this material is located in method section except to explain the parameters for the data coding and analysis.**

Response: We have revised parts of the Methods sub-section “Exposure to conflict violence” (pages 8-9) to make it clearer how “exposure” is measured in our setting and what the concept entails in terms of direct and indirect effects on women (lines 178-181). We note that, as detailed in a previous response, we have included a new section in the Supplementary Material (section 2.1) with the definitions of all types of conflict events in our dataset, which further complements the clarifications about exposure to conflict alluded to by the Reviewer.

We have also followed the Reviewer’s suggestion of giving further information about the ceasefires earlier in the paper, as background information, and have placed this in the last paragraph of the revised Introduction section (lines 120-122). This sets the scene for the subsequent discussion on the ceasefires exploited for our analysis, located in the Methods section (“Ceasefires during the Havana talks”). However, for the sake of brevity of the Introduction and Methods sections in the main paper, and to focus on the main message, we keep the remaining details about the peace process/ceasefires in the Supplementary Material. We hope this is acceptable.

We clarify, in response to the Reviewer’s question about where the data on fetal deaths were obtained from, that all fetal deaths (including miscarriages) and deaths before the 7th day of life were identified from death certificates. See lines 145-147.

Finally, in response to the Reviewer’s comment about possible errors in determining women’s municipality of residence during the study period, we mention in the paper how we conducted cross-checks with different databases to minimize errors in this respect (lines 185-188 main document and subsection *1.3 Construction of the database* in the Supplementary Material) but discuss this as one potential limitation of our study (lines 515-524).

**Results**

**The results are presented in clear and comprehensive way. The figures were helpful in providing graphic representations of the findings in a digestible manner. The results were fascinating. The opportunities of using the RD method are beautifully displayed in these results. Of course the results require good data.**

**Please see the annotated text for some questions that I think must be addressed before a final submission.**

Response: We thank the Reviewer for the editing suggestions made in the annotated text, all of which we have tried to address in the revised text. Please see our first response above for the location of those changes.

**Discussion/strengths and limitations**

**Again well done for the most part but I do have some questions requiring attention in these sections too.**

Response: We thank the Reviewer for the corresponding suggestions made in the annotated text, all of which we have tried to address in the revised text. Please see our first response above for the location of those changes.

With respect to the specific comment recommending the addition of further information on how the conflict could be expected to impact on pregnant women (related also to the final comment by this Reviewer in the Methods section), we have addressed this by fleshing out the sub-section “Exposure to conflict violence” (see previous response under Methods) and also by elaborating further on the possible biological mechanisms related to stress (see our response to the last comment by Reviewer #3). Finally, we have highlighted, in the discussion section, how the lack of data about further indicators of access to (pre- and post-natal) care poses a limitation to our investigation of other potential mechanisms mediating the relationship between conflict violence exposure and pregnancy outcomes (lines 524-530). We hope our revisions and additions are able to respond to the Reviewer’s suggestions, while complemented by the information given in the Supplementary Material so as to maintain the main text as brief as possible and focused on the main messages.

**References**

1.	Imbens GW, Lemieux T. Regression discontinuity designs: A guide to practice. Journal of Econometrics. 2008;142: 615–635. doi:10.1016/j.jeconom.2007.05.001

2.	Bor J, Moscoe E, Mutevedzi P, Newell M-L, Bärnighausen T. Regression discontinuity designs in epidemiology: causal inference without randomized trials. Epidemiology. 2014;25: 729–737. doi:10.1097/EDE.0000000000000138

3.	Lee DS, Lemieux T. Regression Discontinuity Designs in Economics. Journal of Economic Literature. 2010;48: 281–355. doi:10.1257/jel.48.2.281

4.	Calonico S, Cattaneo MD, Farrell MH. Optimal bandwidth choice for robust bias-corrected inference in regression discontinuity designs. The Econometrics Journal. 2020;23: 192–210. doi:10.1093/ectj/utz022

5.	Calonico S, Cattaneo MD, Titiunik R. Robust Nonparametric Confidence Intervals for Regression-Discontinuity Designs. Econometrica. 2014;82: 2295–2326. doi:https://doi.org/10.3982/ECTA11757

6.	Imbens G, Kalyanaraman K. Optimal Bandwidth Choice for the Regression Discontinuity Estimator. Rev Econ Stud. 2012;79: 933–959. doi:10.1093/restud/rdr043

7.	Gelman A, Imbens G. Why High-Order Polynomials Should Not Be Used in Regression Discontinuity Designs. Journal of Business & Economic Statistics. 2019;37: 447–456. doi:10.1080/07350015.2017.1366909

8.	Hobel CJ, Goldstein A, Barrett ES. Psychosocial stress and pregnancy outcome. Clin Obstet Gynecol. 2008;51: 333–348. doi:10.1097/GRF.0b013e31816f2709

9.	Nepomnaschy PA, Welch KB, McConnell DS, Low BS, Strassmann BI, England BG. Cortisol levels and very early pregnancy loss in humans. Proc Natl Acad Sci U S A. 2006;103: 3938–3942. doi:10.1073/pnas.0511183103

10.	Valsamakis G, Chrousos G, Mastorakos G. Stress, female reproduction and pregnancy. Psychoneuroendocrinology. 2019;100: 48–57. doi:10.1016/j.psyneuen.2018.09.031

11.	Hobel CJ, Dunkel-Schetter C, Roesch SC, Castro LC, Arora CP. Maternal plasma corticotropin-releasing hormone associated with stress at 20 weeks’ gestation in pregnancies ending in preterm delivery. Am J Obstet Gynecol. 1999;180: S257-263. doi:10.1016/s0002-9378(99)70712-x
