# Supplementary material for: Conflict violence reduction and pregnancy outcomes: A regression discontinuity design in Colombia
Source: PLoS Med. 2021 Jul 6;18(7):e1003684. doi: 10.1371/journal.pmed.1003684 (PMC8259980; doi:10.1371/journal.pmed.1003684)
Supplement: Attachment — Submitted filename: Respuestas revisores_final.docx [file pmed.1003684.s008.docx]

**-----------------------------------------------------------**

**Requests from Editors:**

1) **Thank you for providing your STROBE checklist. Please replace the line numbers with paragraph numbers per section (e.g. "Methods, paragraph 1"), since there will be no line numbers in the final published paper.**

Response: Done. We have modified the STROBE checklist including paragraph numbers per section.

2) **Please provide the English translation of the Spanish protocol and include it as a Supplementary Information file**

Response: Done. We have included the protocol in English.

3) **Please provide p-values for estimates in lines 308-310**

Response: We have included a test of mean differences in order to show the difference between the means of the conflict events between the two populations (Women in M-p90 municipalities and women in M-p75 municipalities). We have included this information (with p value) in line 310. The estimates of the conflict event mean for each population should not include p-values because there is no a priori hypothesis about this estimator. The new text is:

*Women in M-p90 municipalities were exposed to a higher number of total conflict events on average during pregnancy (7.64; 95% CI 7.62 to 7.66) than women in M-p75 municipalities (6.31; 95% CI 6.29 to 6.32) (Mean difference 1.34 (95% CI 1.31 to 1.36; p<0.001)).*

4) **Please use the "Vancouver" style for reference formatting, and see our website for other reference guidelines** [**https://journals.plos.org/plosmedicine/s/submission-guidelines#loc-references**](https://journals.plos.org/plosmedicine/s/submission-guidelines#loc-references)**.**

Response: We have verified and modified all references style according to guidelines.

a) **Please ensure that weblinks are accessible and dates of access have been updated. For example, the weblink for reference #1 is no longer accessible.**

Response: We have verified all weblinks and updated dates of access.

b) **Please provide a weblink for reference #3**

Response: We have included a weblink for reference #3.

**Comments from Reviewers:**

**Reviewer #1: The authors have done an excellent job of responding to all the concerns of the editors and the multiple reviewers. The paper has been considerably strengthened and makes an excellent contribution to the literature. I have no further comments.**

Response: We really appreciate the Reviewer’s very positive assessment of our paper.

**Reviewer #2: The authors have addressed my points.**

**Michael Dewey**

Response: We really appreciate the Reviewer’s assessment of our paper.
